# Trajectories of clinical characteristics, complications and treatment choices in data-driven subgroups of type 2 diabetes

**DOI:** 10.1007/s00125-024-06147-y

**Published:** 2024-04-16

**Authors:** Xinyu Li, Louise A. Donnelly, Roderick C. Slieker, Joline W. J. Beulens, Leen M. ‘t Hart, Petra J. M. Elders, Ewan R. Pearson, Anoukh van Giessen, Jose Leal, Talitha Feenstra

**Affiliations:** 1 Groningen Research Institute of Pharmacy, Faculty of Science and Engineering, University of Groningen, Groningen, the Netherlands; 2 Division of Population Health and Genomics, Ninewells Hospital and School of Medicine, University of Dundee, Dundee, UK; 3 Department of Cell and Chemical Biology, Leiden University Medical Center, Leiden, the Netherlands; 4 Department of Epidemiology and Data Science, Amsterdam UMC, Location Vrije Universiteit Amsterdam, Amsterdam, the Netherlands; 5 Amsterdam Public Health, Amsterdam Cardiovascular Sciences, Amsterdam, the Netherlands; 6 Julius Center for Health Sciences and Primary Care, University Medical Center Utrecht, Utrecht University, Utrecht, Netherlands; 7 Department of Biomedical Data Sciences, Section Molecular Epidemiology, Leiden University Medical Center, Leiden, the Netherlands; 8 Department of General Practice, Amsterdam University Medical Center, Location Vrije Universiteit Amsterdam, Amsterdam, the Netherlands; 9 National Institute of Public Health and the Environment, Bilthoven, the Netherlands; 10 Health Economics Research Centre, Nuffield Department of Population Health, University of Oxford, Oxford, UK

**Keywords:** Data-driven subgroups, Longitudinal analysis, Real-world data, Routine care, Stratification of diabetes

## Abstract

**Aims/hypothesis:**

This study aimed to explore the added value of subgroups that categorise individuals with type 2 diabetes by *k*-means clustering for two primary care registries (the Netherlands and Scotland), inspired by Ahlqvist’s novel diabetes subgroups and previously analysed by Slieker et al.

**Methods:**

We used two Dutch and Scottish diabetes cohorts (*N*=3054 and 6145; median follow-up=11.2 and 12.3 years, respectively) and defined five subgroups by *k*-means clustering with age at baseline, BMI, HbA_1c_, HDL-cholesterol and C-peptide. We investigated differences between subgroups by trajectories of risk factor values (random intercept models), time to diabetes-related complications (logrank tests and Cox models) and medication patterns (multinomial logistic models). We also compared directly using the clustering indicators as predictors of progression vs the *k*-means discrete subgroups. Cluster consistency over follow-up was assessed.

**Results:**

Subgroups’ risk factors were significantly different, and these differences remained generally consistent over follow-up. Among all subgroups, individuals with severe insulin resistance faced a significantly higher risk of myocardial infarction both before (HR 1.65; 95% CI 1.40, 1.94) and after adjusting for age effect (HR 1.72; 95% CI 1.46, 2.02) compared with mild diabetes with high HDL-cholesterol. Individuals with severe insulin-deficient diabetes were most intensively treated, with more than 25% prescribed insulin at 10 years of diagnosis. For severe insulin-deficient diabetes relative to mild diabetes, the relative risks for using insulin relative to no common treatment would be expected to increase by a factor of 3.07 (95% CI 2.73, 3.44), holding other factors constant. Clustering indicators were better predictors of progression variation relative to subgroups, but prediction accuracy may improve after combining both. Clusters were consistent over 8 years with an accuracy ranging from 59% to 72%.

**Conclusions/interpretation:**

Data-driven subgroup allocations were generally consistent over follow-up and captured significant differences in risk factor trajectories, medication patterns and complication risks. Subgroups serve better as a complement rather than as a basis for compressing clustering indicators.

**Graphical Abstract:**

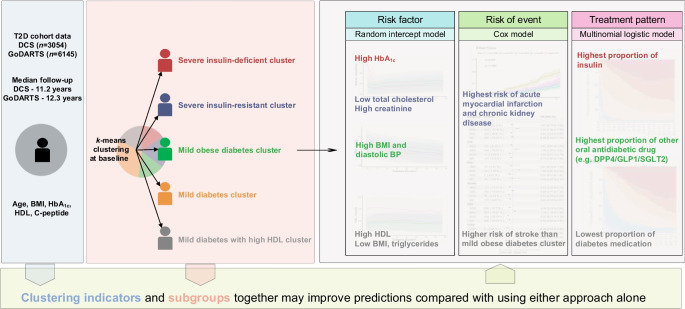

**Supplementary Information:**

The online version of this article (10.1007/s00125-024-06147-y) contains peer-reviewed but unedited supplementary material.


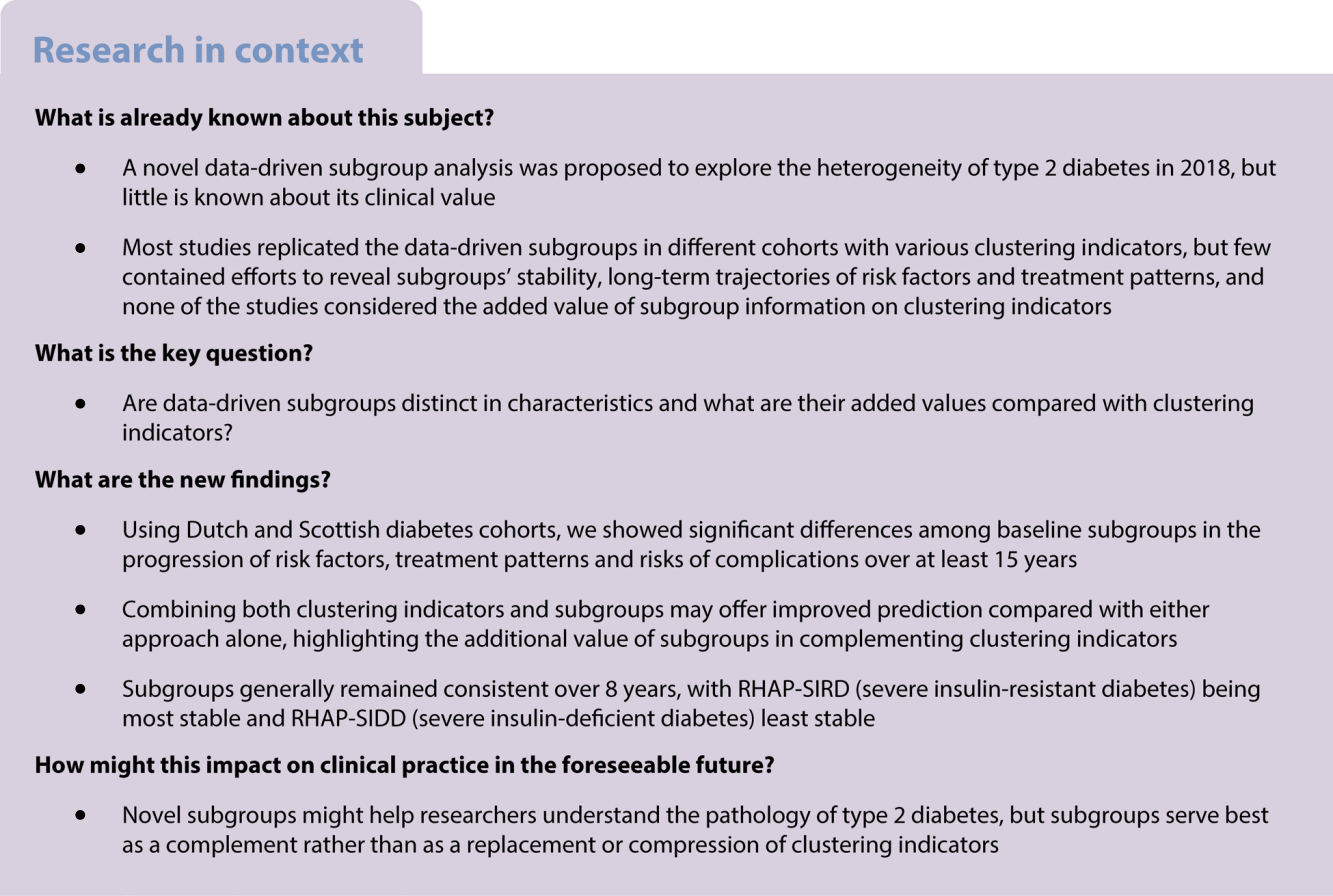


## Introduction

Data-driven clustering analysis has been proposed for categorising type 2 diabetes based on six clinical parameters: age, BMI, HbA_1c_, GAD antibodies and HOMA-2 estimates of beta cell function and insulin resistance [1]. In the study by Ahlqvist et al [1], Swedish individuals with diabetes were stratified into five subgroups, including severe autoimmune diabetes, severe insulin-deficient diabetes (SIDD), severe insulin-resistant diabetes (SIRD), mild obesity-related diabetes (MOD) and mild age-related diabetes (MARD) [1]. These subgroups were reproduced in other countries and cohorts, and their risk profiles studied in both the short and medium term (5 to 15 years) [2–7]. The findings suggest distinct risks of complications and molecular profiles across the subgroups [1–6, 8]. For example, SIRD had a higher frequency of non-alcoholic fatty liver disease and higher risk of developing chronic kidney disease (CKD) [1], and subgroups may help to identify underlying molecular mechanisms related to liver [8], which may provide insights into the diverse aetiology of diabetes.

As part of the Risk Assessment and ProgreSsiOn of Diabetes project (RHAPSODY, https://imi-rhapsody.eu), a new set of risk subgroups clustered based on clinical parameters were defined using Dutch and Scottish diabetes registry data and the original Swedish cohort of individuals with type 2 diabetes [9]. Given that the data originated from routine care, some clinical parameters were slightly modified due to their availability [9]. Replication analyses showed good resemblance between cohorts and also compared with the original Swedish subgroups (developed by Ahlqvist et al [1]) [9, 10], except for the refinement of the original MARD cluster into two new clusters, the mild diabetes subgroup developed by RHAPSODY (RHAP-MD) and the mild diabetes with high HDL-cholesterol subgroup developed by RHAPSODY (RHAP-MDH), following the addition of HDL-cholesterol. Both RHAP-MD and RHAP-MDH exhibited slow glycaemic deterioration, but they showed significantly different molecular signatures [8].

Hence, following up on prior RHAPSODY subgroup research, the current study aims to gain more insight into the clinical relevance of subgroups by studying up to 23 years of follow-up data in two of the original RHAPSODY cohorts. Using contemporary cohorts and a significantly longer follow-up than previous studies, we wanted to: (1) estimate risk factor progression, time to macrovascular complications and treatment patterns by baseline subgroup over at least 15 years; (2) explore the added value of using data-driven subgroups compared with clustering indicators in predicting the progression of risk factors, risk of complications or treatment patterns; and (3) examine the consistency of membership to the data-driven diabetes subgroups over time. Using two distinct cohorts allowed us to validate our findings.

## Methods

### Study design and participants

This retrospective study investigated 9199 individuals with type 2 diabetes in two distinct cohorts: the Hoorn Diabetes Care System (DCS, the Netherlands) and the Genetics of Diabetes Audit and Research in Tayside Scotland (GoDARTS, Scotland). The reporting of study findings followed the STrengthening the Reporting of OBservational studies in Epidemiology (STROBE) guidelines [11], as listed in the electronic supplementary material (ESM) Appendix 1.

Our study’s inclusion criteria consisted of a diagnosis age ≥35, GAD negativity and the availability of complete data for each of the five clustering indicators within 2 years of diagnosis. By omitting the data availability requirement for genome-wide association used in the previous RHAPSODY clustering study [9], we employed more lenient criteria, yielding a slightly larger sample size compared with Slieker et al [9].

The DCS cohort consisted of 3054 individuals (median follow-up=11.2 years) observed over the period 1998–2019 and the GoDARTS cohort consisted of 6145 individuals (median follow-up=12.3 years) over the period 2003–2018 that matched the inclusion criteria (ESM Fig. 2.1). All results were produced for both cohorts, separately.

DCS is a comprehensive dynamic prospective cohort of the natural course of type 2 diabetes from 103 general practitioners (GPs) in the West-Friesland region of the Netherlands, with over 90% of its participants being of European ancestry [12]. At baseline, 52.3% of the participants were men, with a mean age of 63 years. Educational levels varied among participants: 43.3% had a low educational level, 42.1% had a middle educational level and 14.6% had a high educational level [12]. DCS generally represents a Western European, semi-urban population [12]. GoDARTS is a longitudinal cohort that includes individuals with diabetes from the Tayside region of Scotland, with more than 99% of its participants being white [13]. At baseline, 53.3% of the participants were men, with a mean age of 64 years [13]. GoDARTS generally represents a predominantly white population with diabetes in the East of Scotland [13]. Pseudonymised data were collected through electronic record linkage from primary and secondary care data sources [13]. Laboratory measurements of both cohorts have been described in detail in previous studies [9, 12–14] (ESM Appendix 2).

### Outcomes and medications

Macrovascular and microvascular outcomes, including acute myocardial infarction (AMI), congestive heart failure (CHF), peripheral vascular disease (PVD), stroke, CKD and end-stage renal disease (ESRD), were included in this study (ESM Table 2.1).

Medication use was categorised into treatment steps (ESM Table 2.2). These were defined according to the management steps described in the Dutch GP primary care guideline [15], as the relevant guidance for DCS practitioners at the time of data collection, adding information regarding the use of statins and other medication for CVD prevention.

### Clustering

Clustering was done on scaled clustering indicators at baseline, including age at baseline, BMI, HbA_1c_, C-peptide (as a proxy of HOMA-2 estimates of beta cell function and insulin resistance in the absence of fasting glucose in GoDARTS [9]) and HDL-cholesterol (as a risk factor for time to insulin requirement [16]). The baseline for each individual was defined as the observation nearest to diabetes diagnosis. Therefore, it should largely reflect individuals who were either untreated or who only received first line treatment for a brief period (details in Table 1). Men and women were clustered separately and then pooled to avoid sex-dependent differences. Cluster centres were defined as the arithmetic mean of all the values belonging to the cluster. Once clusters were defined, we assigned the same cluster names as those in the original study [1, 9], based on the distribution of cluster characteristics and the lowest Euclidean distance from the previous study [9], including severe insulin-deficient diabetes developed by RHAPSODY (RHAP-SIDD; characterised by high HbA_1c_), severe insulin-resistant diabetes developed by RHAPSODY (RHAP-SIRD; characterised by high C-peptide and age at baseline), mild obesity-related diabetes developed by RHAPSODY (RHAP-MOD; characterised by high BMI), RHAP-MD (characterised by moderate risk factors) and RHAP-MDH (characterised by high HDL-cholesterol) [9].

**Table 1:** Baseline characteristics

Characteristic	DCS (***N***=3054)					GoDARTS (***N***=6145)				
	RHAP-SIDD	RHAP-SIRD	RHAP-MOD	RHAP-MD	RHAP-MDH	RHAP-SIDD	RHAP-SIRD	RHAP-MOD	RHAP-MD	RHAP-MDH
Count (%)	364 (11.92)	655 (21.45)	501 (16.40)	903 (29.57)	631 (20.66)	1094 (17.80)	1061 (17.27)	1141 (18.57)	1722 (28.02)	1127 (18.34)
Male, count (%)	214 (58.8)	420 (64.1)	249 (49.7)	494 (54.7)	335 (53.1)	652 (59.6)	535 (50.4)	693 (60.7)	1048 (60.9)	495 (43.9)
Diabetes duration, years	0.29 ± 0.41	0.43 ± 0.48	0.46 ± 0.53	0.45 ± 0.50	0.47 ± 0.51	0.02 ± 0.10	0.01 ± 0.11	0.02 ± 0.11	0.02 ± 0.10	0.01 ± 0.11
Age at baseline, years	57.32 ± 9.21	67.65 ± 7.37	51.57 ± 7.88	55.24 ± 8.24	65.44 ± 8.21	59.07 ± 9.43	68.15 ± 8.00	51.20 ± 8.09	60.91 ± 9.20	69.93 ± 8.21
BMI, kg/m^**2**^	29.10 ± 4.42	30.36 ± 3.99	38.30 ± 4.85	28.85 ± 3.17	27.20 ± 3.53	29.61 ± 4.75	31.73 ± 4.36	40.17 ± 6.22	30.00 ± 3.95	28.36 ± 4.51
SBP, mmHg	138.73 ± 20.28	146.57 ± 19.54	140.26 ± 16.64	136.66 ± 18.61	145.09 ± 19.68	145.04 ± 19.95	143.56 ± 20.08	144.56 ± 18.58	142.27 ± 19.58	145.91 ± 19.26
DBP, mmHg	82.26 ± 11.12	80.07 ± 9.50	83.30 ± 10.21	80.41 ± 10.08	79.70 ± 9.17	85.68 ± 10.31	80.43 ± 10.69	87.45 ± 10.51	82.56 ± 10.69	79.90 ± 10.60
HbA_1c_, mmol/mol	89.94 ± 12.66	49.19 ± 9.53	53.93 ± 11.83	49.24 ± 8.82	47.74 ± 10.06	99.90 ± 14.81	57.30 ± 14.59	63.03 ± 18.23	52.96 ± 11.10	54.29 ± 13.44
HbA_1c_, %	10.38 ± 1.16	6.64 ± 0.84	7.06 ± 1.08	6.65 ± 0.78	6.50 ± 0.85	11.24 ± 1.38	7.24 ± 1.33	7.79 ± 1.55	6.95 ± 1.01	7.09 ± 1.25
Blood creatinine, μmol/l	77.40 ± 18.17	87.15 ± 18.85	77.19 ± 16.94	78.18 ± 14.64	80.17 ± 14.90	74.95 ± 16.97	85.80 ± 24.13	75.18 ± 19.73	77.61 ± 20.12	79.43 ± 22.54
Triglycerides, mmol/l	2.02 ± 1.02	1.98 ± 0.91	2.15 ± 1.01	1.98 ± 1.01	1.34 ± 0.58	2.78 ± 1.35	2.66 ± 1.42	2.93 ± 1.43	2.65 ± 1.34	1.76 ± 0.81
C-peptide, nmol/l	0.87 ± 0.43	1.49 ± 0.46	1.38 ± 0.47	0.89 ± 0.29	0.83 ± 0.29	1.71 ± 0.77	3.46 ± 0.88	2.02 ± 0.80	1.56 ± 0.57	1.65 ± 0.70
Total cholesterol, mmol/l	5.29 ± 1.18	4.81 ± 1.12	4.97 ± 1.02	5.10 ± 1.21	5.23 ± 1.08	5.71 ± 1.23	5.22 ± 1.16	5.52 ± 1.13	5.32 ± 1.17	5.47 ± 1.19
HDL-cholesterol, mmol/l	1.15 ± 0.27	1.08 ± 0.21	1.07 ± 0.25	1.09 ± 0.20	1.60 ± 0.30	1.14 ± 0.27	1.12 ± 0.23	1.07 ± 0.24	1.09 ± 0.21	1.64 ± 0.33
LDL-cholesterol, mmol/l	3.22 ± 1.03	2.82 ± 0.96	2.94 ± 0.92	3.10 ± 1.04	3.03 ± 0.99	3.40 ± 1.08	2.88 ± 0.93	3.14 ± 0.98	3.15 ± 1.06	2.86 ± 1.04
Treatment steps, count (%)										
No treatment	16 (4.4)	62 (9.5)	69 (13.8)	165 (18.3)	110 (17.4)	356 (32.5)	116 (10.9)	277 (24.3)	372 (21.6)	189 (16.8)
Only CVD	10 (2.7)	208 (31.8)	92 (18.4)	202 (22.4)	177 (28.1)	426 (38.9)	683 (64.4)	565 (49.5)	937 (54.4)	644 (57.1)
Step 1	197 (54.1)	267 (40.8)	246 (49.1)	333 (36.9)	240 (38.0)	129 (11.8)	93 (8.8)	122 (10.7)	132 (7.7)	92 (8.2)
Step 2	109 (29.9)	105 (16.0)	87 (17.4)	166 (18.4)	84 (13.3)	93 (8.5)	52 (4.9)	67 (5.9)	100 (5.8)	57 (5.1)
Step 3	32 (8.8)	13 (2.0)	6 (1.2)	37 (4.1)	18 (2.9)	37 (3.4)	51 (4.8)	40 (3.5)	67 (3.9)	63 (5.6)
Other OAD	0 (0)	0 (0)	1 (0.2)	0 (0)	2 (0.3)	53 (4.8)	66 (6.2)	70 (6.1)	114 (6.6)	82 (7.3)

Data are shown as mean±SD unless otherwise stated

Treatment steps are defined as no treatment (diet and exercise), only CVD treatment (Anatomical Therapeutic Chemical Classification System: C01–C10), step 1 (adding metformin [A10BA02] or repaglinide and nateglinide [A10BX]), step 2 (adding sulfonylurea [A10BB]), step 3 (adding insulin [A10A]) and other OAD (dipeptidyl peptidase-4 inhibitors [A10BH], glucagon-like peptide-1 [A10BJ], α-glucosidase inhibitors [A10BF], sodium–glucose cotransporter 2 inhibitors [A10BK], thiazolidinediones [A10BG], liraglutide [A10BX07], dapagliflozin [A10BX09])

### Statistical analysis

Subgroups identified at baseline were compared with the previously published RHAPSODY subgroups [9], considering the latter as the reference. The agreement was assessed based on sensitivity, specificity, specific agreement [17], overall accuracy rate along with a 95% CI and overall κ indices of agreement [18].

Missing data (mean of 0.6% in DCS and 8.1% in GoDARTS; ESM Table 2.3) were omitted in their respective analyses to avoid excessive use of imputed data as observational evidence.

We reported baseline characteristics for each subgroup using frequencies (%) for categorical variables or mean (SD) for continuous variables. Trajectories of related clinical parameters (BMI, HbA_1c_, HDL-cholesterol, systolic BP [SBP], diastolic BP [DBP], total cholesterol, LDL-cholesterol, blood creatinine and triglycerides) were visualised by plotting subgroup annual means, along with 1 SD boundaries based on observed variance within subgroups. The random intercept model was used to analyse longitudinal trajectory data with discrete subgroup membership, sex and diabetes duration as covariates.

Kaplan–Meier methods were applied to plot cumulative incidence for first events of each outcome since diagnosis of diabetes by subgroups. Group comparisons and pairwise comparisons were conducted by logrank tests, applying Benjamini–Hochberg correction [19] to adjust for multiple comparisons. A Cox regression model with diabetes duration as the time scale, left truncated at each individual’s diagnosis of diabetes, was conducted to calculate the HR (95% CI). The Cox model was also adjusted for age at baseline and sex. Schoenfeld tests were applied to evaluate the proportional hazard assumption, and violation was indicated by *p*<0.05 [20].

We visualised medication patterns reflecting the proportion of individuals within each subgroup in each treatment step over the follow-up period by area graphs. Multinomial logistic regression, in which treatment steps were dependent variables, with discrete subgroup membership, diabetes duration and sex as covariates, was conducted to compare the proportion in each treatment step between subgroups.

The models described above were re-estimated using clustering indicators at baseline (HbA_1c_, C-peptide, HDL-cholesterol, age and BMI), with and without discrete subgroup membership data, to analyse the longitudinal risk factor trajectories, risk of complications and medication patterns. Akaike’s information criterion (AIC) and relative likelihood (RL) were applied to compare the information loss and fitting of models [21]. Smaller AIC values indicate better goodness of fit. The *p* value for the comparison of AIC differences was then indicated by $RL={\text{exp}}(\frac{{AIC}_{min}-AIC}{2})$. We visualised the results on a heatmap, using colours to indicate scaled AIC and text to indicate RL.

Two clustering algorithms were repeated with durations of 2–4, 4–6 and 4–8 years from diagnosis to assess the cluster consistency over time as follows: (1) de novo clustering (i.e. repeating *k*-means clustering); or (2) centre-based reallocation (i.e. assigning individuals to the subgroup with the lowest Euclidean distance to cluster centres identified at baseline). The agreement between estimated subgroups over time and subgroups identified at baseline was assessed, and the cluster migration pattern was presented graphically for individuals with available clustering indicators in all four 2 year intervals (GoDARTS *n*=4914; DCS *n*=2756), along with the top ten transition trajectories. An analysis of the associated risk factors and treatment patterns was visualised in the same manner for the most representative movements. We used the Cox regression model to compare the risk of complications for those who moved between severe subgroups (including RHAP-SIRD and RHAP-SIDD) and mild subgroups (including RHAP-MD, RHAP-MOD and RHAP-MDH).

All analyses were performed in R [22] (version 4.1.0: https://www.r-project.org/) and R studio (version 1.4.1717: https://www.rstudio.com/) (ESM Appendix 3).

## Results

### Baseline characteristics and the progression of clinical parameters over time

Our current subgroups identified at baseline, which were based on a larger sample size of individuals than previously published RHAPSODY subgroups [9] (2953 individuals in DCS), showed a good resemblance with an accuracy of 0.92 (95% CI 0.91, 0.93) (ESM Table 4.1), despite a slight change in clustering centroids (ESM Table 4.2).

Significant differences in baseline clustering indicators, treatment patterns and other clinical parameters were observed among subgroups identified at baseline (Table 1, ESM Figs 4.1–4.4).

**Fig. 1:**
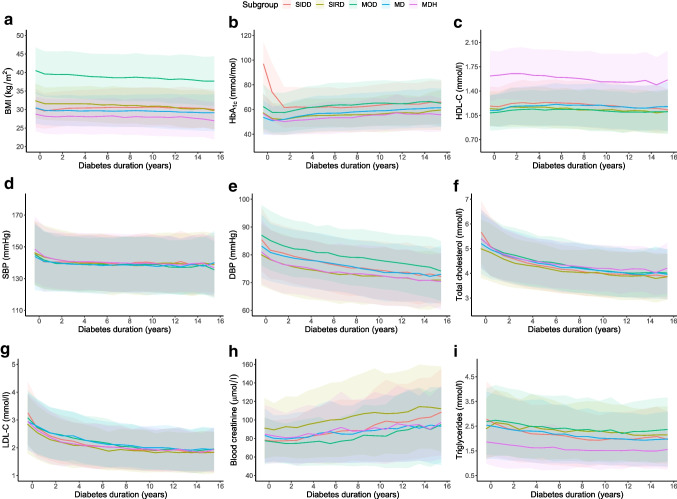
Progression of clinical parameters over time based on subgroups identified at diagnosis in the GoDARTS cohort. Here, SIDD, SIRD, MOD, MD and MDH refer to RHAP-SIDD, RHAP-SIRD, RHAP-MOD, RHAP-MD and RHAP-MDH, respectively. HDL-C and LDL-C refer to HDL-cholesterol and LDL-cholesterol, respectively. Values of selected parameters over time in each cluster are shown. The data are represented as mean values (solid line) ±SD (shaded areas). Missing values were removed

Figure 1 and ESM Fig. 4.5 show that the ranking of risk factors across baseline subgroups remained relatively unchanged throughout follow-up for those risk factors used to characterise specific subgroups (e.g. the subgroup characterised by high HDL-cholesterol at baseline recorded the highest mean HDL-cholesterol during follow-up). The exception was for the trajectory of HbA_1c_ as observed in GoDARTS (Fig. 1b), where the RHAP-MOD subgroup crossed with the RHAP-SIDD subgroup after 4 years from diagnosis and became the subgroup with the highest mean HbA_1c_. Random intercept models (ESM Table 4.3) indicated that subgroups’ properties over time are not only visually distinct but also statistically significantly different. Specifically, compared with RHAP-SIDD, RHAP-SIRD had significantly higher creatinine (an average difference of 12.65 μmol/l across the two cohorts) and RHAP-MDH had significantly lower triglyceride (an average difference of 0.48 mmol/l).

### Diabetes-related complications by subgroup

The risks of developing AMI, CHF, stroke, CKD and ESRD are significantly different across all subgroups (ESM Figs 5.1, 5.2). At 10 years after diagnosis, the RHAP-SIRD subgroup had the highest incidence of AMI (28.43%; 5.12%), CHF (10.66%; 7.87%), PVD (1.78%; 1.48%) and CKD (75.32%; 57.08%) and RHAP-MDH had the highest incidence of stroke (9.45%; 5.32%) in both GoDARTS and DCS.

**Fig. 2:**
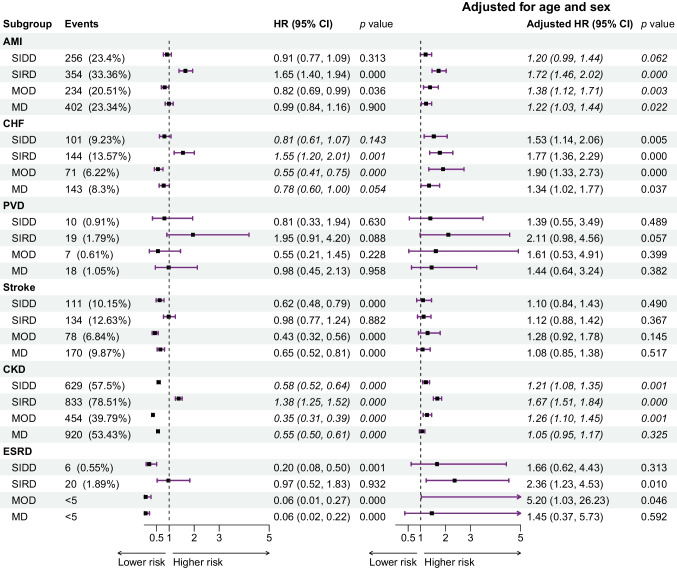
The results of Cox regression analysis of GoDARTS. Here, SIDD, SIRD, MOD, MD and MDH refer to RHAP-SIDD, RHAP-SIRD, RHAP-MOD, RHAP-MD and RHAP-MDH, respectively. MDH is the reference group. Italics indicate that the proportional hazard assumption has not been fulfilled

Proportional hazard assumptions are fulfilled except for CHF and CKD in GoDARTS (ESM Table 5.1). In both GoDARTS and DCS, Cox models (Fig. 2, ESM Fig. 5.3) indicated that compared with RHAP-MDH, HRs for RHAP-SIRD were significantly different (*p*<0.05) and the highest among all other subgroups for AMI. Although RHAP-MDH is the so-called mild subgroup, HRs for RHAP-MDH were significantly (*p*<0.05) higher than RHAP-MOD for stroke in both GoDARTS (HR 2.35 [95% CI 1.78, 3.1]) and DCS (HR 2.31 [1.22, 4.38]) (ESM Tables 5.2, 5.3).

Multiple comparisons of survival rate curves indicated that CHF and CKD incidence was significantly higher in the RHAP-SIRD subgroup than in RHAP-MOD and RHAP-MD (ESM Tables 5.4, 5.5) in both cohorts. Although these higher risks of complication might be driven mainly by the higher age of the RHAP-SIRD and RHAP-MDH subgroups, Cox models adjusted for age and sex still indicated significantly higher HRs of AMI and CKD in RHAP-SIRD compared with RHAP-MDH for both GoDARTS (AMI HR 1.72 [1.46, 2.02] and CKD HR 1.67 [1.51, 1.84]; unadjusted AMI HR 1.65 [1.40, 1.94] and unadjusted CKD HR 1.38 [1.25, 1.52]) and DCS (AMI HR 2.86 [1.35, 6.04] and CKD HR 1.77 [1.51, 2.08]; unadjusted AMI HR 3.53 [1.68, 7.41] and unadjusted CKD HR 2.02 [1.72, 2.36]) (Fig. 2, ESM Fig. 5.3).

### Treatment patterns

**Fig. 3:**
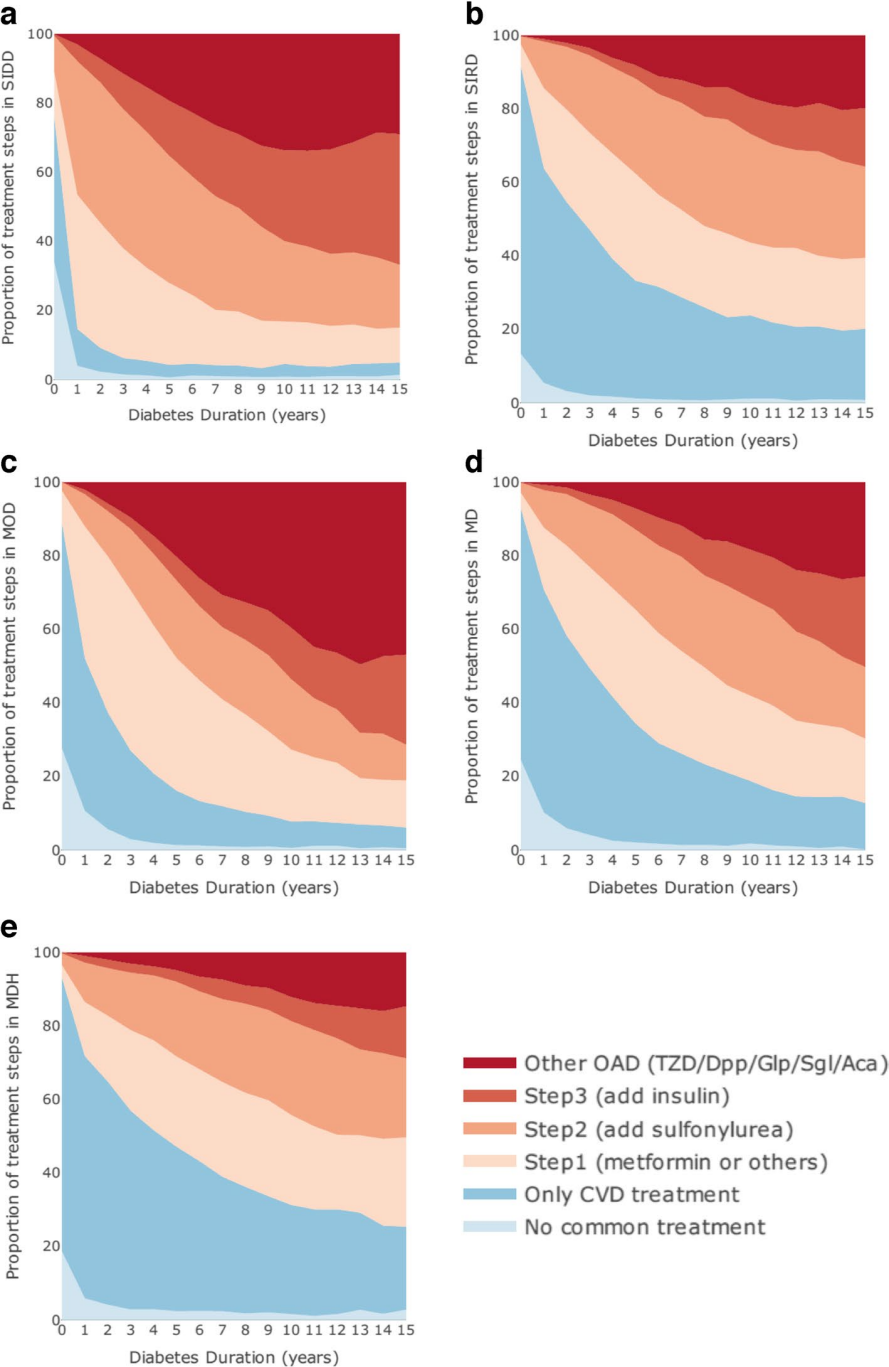
Area graph of treatment steps per individual over time for each subgroup in the GoDARTS cohort. Here, SIDD, SIRD, MOD, MD and MDH refer to RHAP-SIDD, RHAP-SIRD, RHAP-MOD, RHAP-MD and RHAP-MDH, respectively. On the *x*-axes, 0 indicates the period from diagnosis to less than 1 year after diagnosis; similarly, 1 indicates the period from 1 year after diagnosis to less than 2 years after diagnosis, etc. The treatment step was defined by an individual’s first available observation in each diabetes duration interval. Treatment steps are defined as no common treatment (diet and exercise), only CVD treatment (Anatomical Therapeutic Chemical Classification System: C01–C10), step 1 (adding metformin [A10BA02] or repaglinide and nateglinide [A10BX]), step 2 (adding sulfonylurea [A10BB]), step 3 (adding insulin [A10A]) and other OAD (dipeptidyl peptidase-4 inhibitors [Dpp; A10BH], glucagon-like peptide-1 [Glp; A10BJ], α-glucosidase inhibitors [Aca; A10BF], sodium–glucose cotransporter 2 inhibitors [Sgl; A10BK], thiazolidinediones [TZD; A10BG], liraglutide [A10BX07], dapagliflozin [A10BX09])

Clear variations in treatment patterns across subgroups over diabetes duration were seen (Fig. 3, ESM Fig. 6.1). In both cohorts, RHAP-MOD had the highest proportion of prescribing other oral antidiabetic drugs (OADs; otherwise known as oral glucose-lowering drugs), including dipeptidyl peptidase-4 inhibitors, glucagon-like peptide-1 analogues, α-glucosidase inhibitors, sodium–glucose cotransporter 2 inhibitors and thiazolidinediones (on average 23.83% in GoDARTS and 3.55% in DCS). Among these, the prescriptions for thiazolidinediones (6.16%) and dipeptidyl peptidase-4 inhibitors (5.15%) were the highest in GoDARTS, whereas both were less than 1% in DCS, reflecting differences in prescribing practices between the two countries. A substantially higher proportion of individuals with RHAP-SIDD received diabetes medication than other subgroups in both cohorts. More than half of individuals with RHAP-SIDD were prescribed insulin or other OADs at 10 years of diagnosis in GoDARTS (55.15%; 27.56% insulin and 27.58% other OADs) and DCS (52.48%; 49.65% insulin and 2.84% other OADs). In both GoDARTS and DCS, this proportion was the lowest in RHAP-MDH (17.34%; 14.91%), followed by RHAP-SIRD (24.58%; 17.63%), RHAP-MD (31.14%; 26.88%) and RHAP-MOD (51.16%; 30.56%), indicating that individuals with RHAP-SIDD received the most intensive glucose control treatment, followed by RHAP-MOD. Multinomial logistic regression results (ESM Table 6.1) indicated that treatment patterns were not only visually distinct but also statistically different among all subgroups. For example, for RHAP-SIDD relative to RHAP-MD, the relative risk for using insulin (step 3) to no common treatment would be expected to increase by a factor of 3.07 (95% CI 2.73, 3.44) in GoDARTS and 11.80 (95% CI 8.98, 15.50) in DCS, given the other variables in the model are held constant.

### Comparison between subgroups and clinical features to predict outcomes

Using only clustering indicators compared with discrete cluster memberships resulted in better fitting models in both cohorts (ESM Fig. 7.1; details in ESM Appendix 7), except for the Cox model for stroke, in which discrete subgroup membership performed slightly better than clustering indicators, though not significantly, as reflected by RL>0.1. Yet adding discrete cluster memberships to clustering indicators achieved significantly lower AIC and thus a significantly better fit for the trajectories of BMI, HbA_1c_, SBP, blood creatinine and treatment patterns in both cohorts. A detailed example can be found in ESM Appendix 8.

### Consistency of subgroups classification over time

In general, clusters were consistent over 8 years with an accuracy ranging from 59% to 72% (ESM Table 9.1). By de novo clustering over 8 years since diagnosis, on average, 53% of individuals migrated to other subgroups with shifted cluster centres (ESM Tables 9.1–9.3). The accuracy of allocation decreased by 4.52% from 0.70 in 2–4 years to 0.67 in 6–8 years in DCS, and by 7.11% from 0.64 in 2–4 years to 0.59 in 6–8 years in GoDARTS. The κ (0.49–0.62) indicated a moderate to substantial agreement over time compared with subgroups identified at baseline. The specificity (ESM Table 9.3) was 0.91 on average, while the sensitivity and specific agreement were around 0.65 and 0.64 (lowest for RHAP-SIDD with average values of 0.25 and 0.28, respectively). By the centre-based reallocation method, accuracy (0.61–0.72), κ (0.51–0.64) and the proportion of individuals staying in the same cluster (0.46–0.72) improved by an average of 4.22%, 6.39% and 5.73% compared with the de novo clustering method.

**Fig. 4:**
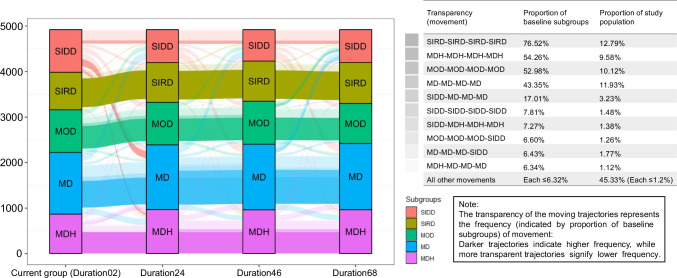
Subgroups’ redistribution using the centre-based reallocation method over time and characteristics of common trajectories in the GoDARTS cohort (*N*=4919). Here, SIDD, SIRD, MOD, MD and MDH refer to RHAP-SIDD, RHAP-SIRD, RHAP-MOD, RHAP-MD and RHAP-MDH, respectively. The figure shows the subgroups identified based on clinical characteristics within the first 2, 2–4, 4–6 and 6–8 years of diagnosis of type 2 diabetes, represented as Duration02, Duration24, Duration46 and Duration68, respectively, along with the top ten most frequent moving trajectories using the centre-based reallocation approach. Only individuals with information available for all four periods were included in the redistribution graph

By the centre-based reallocation method, in GoDARTS, the RHAP-SIRD subgroup displayed the highest stability, with 77% of individuals remaining in the same cluster for 8 years. In contrast, the RHAP-SIDD subgroup was the least stable, with only 8% of individuals staying in the same cluster (Fig. 4). The most common transitions for RHAP-SIDD were to RHAP-MD (17%) and RHAP-MDH (7%) within the initial 2 years, with individuals maintaining their position in that subgroup for the subsequent 6 years. These individuals had a higher proportion receiving insulin-based control treatment and a greater decrease in HbA_1c_ levels than individuals who were assigned to RHAP-MD or RHAP-MDH initially and stayed for the next 8 years (ESM Fig 9.1). Similar results could be found in DCS (ESM Figs 9.2, 9.3)

No significant difference in macrovascular disease risk was found between individuals transitioning from mild to severe subgroups and those remaining in the severe subgroup over 2 years (ESM Figs 9.4, 9.5). For those in severe subgroups initially (ESM Figs 9.6, 9.7), those who stayed in severe subgroups for 2 years had a significantly higher risk of CKD (GoDARTS: HR 1.67 [1.48, 1.89]; DCS: HR 2.4 [1.92, 3.01]) than those who moved to mild subgroups. For individuals in mild subgroups initially, those who shifted to severe subgroups in 2 years had a higher risk of AMI (GoDARTS: HR 1.5 [1.25, 1.8]; DCS: HR 2.88 [1.39, 5.95]) and CHF (GoDARTS: HR 1.38 [1.02, 1.88]; DCS: HR 3.28 [1.99, 5.42]) than those who stayed in mild subgroups. Standardisation for age and sex did not change these findings (ESM Figs 9.6, 9.7).

## Discussion

Using a much longer follow-up, we confirm previous findings [2] that data-driven subgroups effectively recognised individual phenotype heterogeneity, as reflected by significant differences in risk factor progression, complication risks and treatment patterns. Integrating subgroup information with clustering indicators may offer improved prediction of progression variation compared with either approach alone, emphasising the complementary role of subgroups rather than replacing continuous indicators. While most subgroups remain generally consistent over time, the RHAP-SIDD subgroup is notably volatile, indicating the necessity to expand insights from baseline subgroups to longitudinal status.

Significant differences in clinical parameters were observed not only at baseline but also over time among the subgroups, such as high BMI and DBP in RHAP-MOD, high HDL-cholesterol and low triglycerides in RHAP-MDH, and high blood creatinine and low total cholesterol in RHAP-SIRD. The only exception was that the trajectory of HbA_1c_ in individuals with RHAP-MOD was poorly controlled for longer diabetes duration and worse than for RHAP-SIDD individuals.

Prior research has demonstrated that the SIRD subgroup exhibited higher risks of liver disease, macroalbuminuria, nephropathy, CKD and ESRD [1, 2, 6, 23]. Our analysis also revealed that RHAP-SIRD presented a higher risk of AMI, CHF, PVD, CKD and ESRD compared with other subgroups. By definition, subgroups varied in clustering indicators, such as age at baseline, which are among the risk factors for these complications. Upon adjusting for age, RHAP-SIRD maintained a significantly higher risk of AMI and CKD compared with other subgroups.

Treatment patterns varied significantly among subgroups, with the highest proportions of other OADs and overall glucose control treatment observed in RHAP-MOD and RHAP-SIDD subgroups, respectively. This suggests that physicians’ treatment decisions for individuals within these subgroups differed, likely due to variations in age and other clustering indicators, as they were unaware of the individuals’ subgroup membership.

The significant differences in disease progression, complication risks and treatment patterns among subgroups highlight their utility in understanding the underlying pathways of disease progression. Slieker et al [8] demonstrated that diabetes subgroups reveal distinct molecular mechanisms in key metabolic tissues, uncovering varied causes of the disease that are not apparent when it is viewed uniformly. Beyond aiding in aetiological understanding, subgroups may also be useful for predictive purposes. However, data-driven subgroups have been criticised for their unsuitability in predicting outcomes, such as drug response or complications [4, 24]. Our study partially supports this critique, as we found that using the clustering indicators may perform better than solely using subgroups for prediction. This is due to subgroups compressing data from several individual indicators, leading to information loss. However, we found that combining subgroup membership (e.g. SIDD) with the clustering indicators (e.g. age, BMI, etc.) often enhanced the performance of the progression models, indicating a potential predictive benefit from including subgroup information.

As expected, allocating individuals with long diabetes duration based on the lowest distance to baseline centroid leads to higher consistency of baseline subgroups. Practically, using cluster centres enables easy assignment of individuals to subgroups without requiring information about other individuals. To enhance accuracy, cluster centres can be periodically updated according to the latest cohort characteristics, similar to routine updates in risk prediction models. Furthermore, our study revealed RHAP-SIRD to be the most consistent subgroup over time, with over 70% of individuals remaining for over 8 years, signifying its distinct, partially divergent aetiology. This aligns with prior research identifying SIRD as the most genetically unique subgroup [25], exhibiting an insulin resistance molecular signature [8] and lacking associations with the type 2 diabetes locus in the *TCF7L2* gene or insulin secretion risk scores, contrary to SIDD and MOD [1, 25–27].

Ahlqvist’s original study was designed to deepen the understanding of diabetes heterogeneity and enhance individualised treatment by identifying baseline phenotypes [1]. To fully benefit from the long follow-up information available, we expanded this concept to include more than just baseline subgroups, attempting to explore the dynamics of disease. As expected, we observed changes in subgroup memberships over time, reflecting the combination of treatment effects and underlying phenotypes. For example, we found that more than 28% of individuals transitioned to other subgroups after 2 years. These temporal dynamics might be shaped by interactions between disease heterogeneity, adherence to treatment and treatment efficacy. Diabetes heterogeneity, such as distinct molecular signatures and genetic characteristics [1, 8], may result in individuals consistently belonging to specific subgroups with unique phenotypes. However, the treatment meanwhile aims to shift individuals toward milder subgroups. For example, newly diagnosed individuals who subsequently meet guideline-based treatment targets (53 mmol/mol (7%) HbA_1c_ [28, 29], 0.9 mmol/l HDL-cholesterol [30], 25 kg/m^2^ BMI [31]) will either remain or progress to the RHAP-MD subgroup over time, whereas insufficient risk factor control could result in increased progression to severe subgroups.

The longitudinal nature of our data allowed us to estimate the impact of changes in subgroup membership over time. We found that complication risks were more closely associated with individuals’ current subgroups rather than the initial subgroups they were assigned at baseline. The risks of complications for individuals progressing from mild to severe subgroups were similar to those for individuals initially allocated to and remaining in severe subgroups. Also, individuals progressing from severe to mild subgroups showed complication risks lower than for those who remained in severe subgroups. Thus, an initial allocation to a mild subgroup did not necessarily translate into mild progression, and efforts should aim at achieving or maintaining mild subgroup status. This might suggest the importance of periodically re-clustering with changing risk factors as the disease progresses to capture the evolving dynamics and guide more informed decision-making.

Our study is not without limitations. First, C-peptide, one of the five clustering indicators, was assumed to be constant, due to the lack of follow-up data. This might overestimate subgroup consistency, but its impact is likely limited due to C-peptide’s stability [1]. Second, we estimated the treatment pattern from observed data and ignored censoring (ESM Figs 10.1, 10.2), which might underestimate the proportion of individuals taking the most intensive treatment steps. Third, due to the unavailability of fasting glucose data in GoDARTS, we were unable to replicate Ahlqvist’s subgroups within this registry. Ahlqvist’s method captures two key pathogenic mechanisms: insulin deficiency and resistance, indicated by HOMA-IR and HOMA-B. We used C-peptide instead, which may obscure the pathology link with type 2 diabetes. Nevertheless, considering the high sensitivity and specificity of RHAP-SIDD (72% and 100%) and RHAP-SIRD (67% and 89%) in relation to Ahlqvist’s subgroups (ESM Fig. 11.1), our findings for RHAP-SIDD and RHAP-SIRD may offer insights for Ahlqvist’s subgroups. Of note, SIDD had worse beta cell function than other subgroups described by Ahlqvist et al [1], and this was partially conveyed by the lower C-peptide of RHAP-SIDD among the RHAPSODY subgroups. Since C-peptide is generally stable over time [9], but beta cell function progressively declines [32], we might expect even worse stability for SIDD in Ahlqvist’s subgroups. Fourth, DCS registered events were based on self-report, which could lead to an underestimation of events. However, a validation study found events to be well reported, with 86% sensitivity and 90% specificity [12]. Finally, our cohorts, predominantly consisting of white individuals, may limit the generalisability of findings to other settings.

In conclusion, the significant differences observed in subgroups’ trajectories raise the possibility of identifying and understanding different phenotypes of type 2 diabetes. Also, subgroup information may improve prediction when added as a predictor. This lays the foundation for considering diabetes subgroups as complementary to, rather than replacements for, individual indicators.

### Supplementary Information

Below is the link to the electronic supplementary material.

Supplementary file1 (PDF 4751 KB)

## Data Availability

The data that support the findings of this study are available from Amsterdam University Medical Center and the University of Dundee, and were accessed by the authors via a formal data request procedure and as a part of the RHAPSODY project. Therefore, the data are not publicly available. Steering committees of the individual cohorts will consider reasonable requests for sharing of de-identified individual-level data.

## Abbreviations

AIC: Akaike’s information criterion
AMI: Acute myocardial infarction
CHF: Congestive heart failure
CKD: Chronic kidney disease
DBP: Diastolic BP
DCS: Hoorn Diabetes Care System
ESRD: End-stage renal disease
GoDARTS: Genetics of Diabetes Audit and Research in Tayside Scotland
GP: General practitioner
MARD: Mild age-related diabetes
MOD: Mild obesity-related diabetes
OAD: Oral antidiabetic drug
PVD: Peripheral vascular disease
RHAPSODY: Risk Assessment and ProgreSsiOn of Diabetes project
RHAP-MD: Mild diabetes subgroup developed by RHAPSODY
RHAP-MDH: Mild diabetes with high HDL-cholesterol subgroup developed by RHAPSODY
RHAP-MOD: Mild obesity-related diabetes developed by RHAPSODY
RHAP-SIDD: Severe insulin-deficient diabetes developed by RHAPSODY
RHAP-SIRD: Severe insulin-resistant diabetes developed by RHAPSODY
RL: Relative likelihood
SBP: Systolic BP
SIDD: Severe insulin-deficient diabetes
SIRD: Severe insulin-resistant diabetes
