# Supplementary material for: Trajectories of clinical characteristics, complications and treatment choices in data-driven subgroups of type 2 diabetes
Source: Diabetologia. 2024 Apr 16;67(7):1343–55. doi: 10.1007/s00125-024-06147-y (PMC11153261; doi:10.1007/s00125-024-06147-y)
Supplement: Supplementary file 1 — Supplementary file1 (PDF 4751 KB) [file 125_2024_6147_MOESM1_ESM.pdf]

Supplementary materials for

Trajectories of clinical characteristics, complications and treatment choices in data-driven subgroups of type 2 diabetes

Diabetologia

Xinyu Li[1] (ORCID: 0000-0002-0225-6937), Louise A. Donnelly[2], Roderick C. Slieker (0000-0003-0961-9152)[3,4,5], Joline W.J. Beulens (0000-0002-4181-0937)[4,5,6], Leen M. 't Hart (0000-0003-4401-2938)[3,4,5,7], Petra J. M. Elders (0000-0002-5907-7219)[5,8], Ewan R. Pearson (0000-0001-9237-8585)[2], Anoukh van Giessen (0000-0003-4521-9500)[9], Jose Leal (0000-0001-7870-6730)[10*] & Talitha Feenstra (0000-0002-5788-0454)[1,9*]

[1]Groningen Research Institute of Pharmacy, Faculty of Science and Engineering, University of Groningen, Groningen, the Netherlands

[2] Division of Population Health and Genomics, Ninewells Hospital and School of Medicine, University of Dundee, Dundee, UK

[3]Department of Cell and Chemical Biology, Leiden University Medical Center, Leiden, the Netherlands

[4]Department of Epidemiology and Data Science, Amsterdam UMC, location Vrije Universiteit Amsterdam, Amsterdam, the Netherlands

[5]Amsterdam Public Health, Amsterdam Cardiovascular Sciences, Amsterdam, the Netherlands

[6]Julius Center for Health Sciences and Primary Care, University Medical Center Utrecht, Utrecht University, Utrecht, Netherlands

[7]Department of Biomedical Data Sciences, Section Molecular Epidemiology, Leiden University Medical Center, Leiden, the Netherlands

[8]Department of General Practice, Amsterdam University Medical Center, Location VUMC, Amsterdam, the Netherlands

[9]National Institute of Public Health and the Environment, Bilthoven, the Netherlands

[10]Health Economics Research Centre, Nuffield Department of Population Health, University of Oxford, Oxford, UK

*Jose Leal and Talitha Feenstra share last authorship.

List of Contents

ESM Appendix 1 STROBE Statement 4
ESM Appendix 2 Clinical data measurement 6
ESM Appendix 3 R packages applied in the analysis 13
ESM Appendix 4 Baseline characteristics and the progression of clinical parameters over time 14
ESM Appendix 5 Diabetes-related complications by subgroups 22
ESM Appendix 6 Treatment patterns 30
ESM Appendix 7 Comparison between subgroups and clinical features to predict outcomes 32
ESM Appendix 8 Example of the added value of subgroups on top of clustering indicators 43
ESM Appendix 9 Consistency of subgroups classification 45
ESM Appendix 10 The number of observed individuals over time 58
ESM Appendix 11 Consistency of subgroups classification compared to Ahlqvist et al.'s subgroups [3] . 60

List of Tables

ESM Table 1.1 STROBE Statement—checklist of items that should be included in reports of observational studies 4
ESM Table 2.1 Definitions of outcomes 7
ESM Table 2.2 Treatment steps used for classification of medication use 8
ESM Table 2.3 The number and proportion of missing data 9
ESM Table 4.1 The overall statistics of comparing subgroups in this study to subgroups identified in a previous study [1] 14
ESM Table 4.2 Scaled clustering centers of subgroups at baseline 15
ESM Table 4.3 Random intercept models of clinical parameters 16
ESM Table 5.1 Results of Schoenfeld tests checking the proportional hazards assumption 22
ESM Table 5.2 Cox regression analysis comparing event rates between subgroups in GoDARTS 23
ESM Table 5.3 Cox regression analysis comparing event rates between subgroups in DCS 24
ESM Table 5.4 Pairwise comparisons comparing event rates between subgroups using Log-Rank test in GoDARTS 25
ESM Table 5.5 Pairwise comparisons comparing event rates between subgroups using Log-Rank test in DCS . 26
ESM Table 6.1 The results of multinomial logistic regressions (relative risk ratios) for comparing treatment steps 30
ESM Table 7.1 The result of all random intercept models of DCS for the comparison between subgroups and clinical features to predict outcomes 33
ESM Table 7.2 The result of all Cox models of DCS for the comparison between subgroups and clinical features to predict outcomes 35
ESM Table 7.3 The results of all multinomial models of DCS for the comparison between subgroups and clinical features to predict outcomes 37
ESM Table 7.4 The result of all random intercept models of GoDARTS for the comparison between subgroups and clinical features to predict outcomes 38

ESM Table 7.5 The result of all Cox models of GoDARTS for the comparison between subgroups and clinical features to predict outcomes 40
ESM Table 7.6 The results of all multinomial models of GoDARTS for the comparison between subgroups and clinical features to predict outcomes 42
ESM Table 9.1 Subgroup redistributions' overall statistics over time 45
ESM Table 9.2 Scaled clustering centers 47
ESM Table 9.3 Summary of stability performance for subgroups, derived from the confusion matrix 49

List of Figures

ESM Figure 2.1 Data selection process flowchart 10
ESM Figure 2.2 The process chart of adjusting diabetes duration of DCS 12
ESM Figure 4.1 Violin plots and pairwise comparisons of clustering indicators at baseline between subgroups of DCS 17
ESM Figure 4.2 Violin plots and pairwise comparisons of clustering indicators at baseline between subgroups of GoDARTS 18
ESM Figure 4.3 Violin plots and pairwise comparisons of other risk factors at baseline between subgroups of DCS 19
ESM Figure 4.4 Violin plots and pairwise comparisons of other risk factors at baseline between subgroups of GoDARTS 20
ESM Figure 4.5 Progression of clinical parameters over time based on subgroups identified at diagnosis in the DCS cohort 21
ESM Figure 5.1 Kaplan Meier plots of GoDARTS 27
ESM Figure 5.2 Kaplan Meier plots of DCS 28
ESM Figure 5.3 The results of Cox regression analysis of DCS 29
ESM Figure 6.1 Area graph of treatment steps per individual over time for each subgroup in the DCS cohort .. 31
ESM Figure 7.1 Comparisons between model fits with different formats of covariates to indicate subgroups 32
ESM Figure 9.1 Characteristics for subgroups considering moving over time in GoDARTS 51
ESM Figure 9.2 Subgroups' redistribution using the center-based reallocation method over time and characteristics of common trajectories in the DCS cohort (N=2,756) 52
ESM Figure 9.3 Characteristics for subgroups considering moving over time in DCS 53
ESM Figure 9.4 Cox regression analysis comparing event rates between mild and severe subgroups in GoDARTS 54
ESM Figure 9.5 Cox regression analysis comparing event rates between mild and severe subgroups in DCS 55
ESM Figure 9.6 Cox regression analysis comparing event rates between mild and severe subgroups with known baseline subgroups in GoDARTS in 2 years 56
ESM Figure 9.7 Cox regression analysis comparing event rates between mild and severe subgroups with known baseline subgroups in DCS in 2 years 57
ESM Figure 10.1 The number of observed individuals over time in DCS 58
ESM Figure 10.2 The number of observed individuals over time in GoDARTS 59
ESM Figure 11.1 Subgroups' consistency compared to Ahlqvist et al.'s subgroups [3] in DCS 60

ESM Appendix 1 STROBE Statement

ESM Table 1.1 STROBE Statement—checklist of items that should be included in reports of observational studies

	Item No	Recommendation	Page number
**Title and abstract**	1	(*a*) Indicate the study's design with a commonly used term in the title or the abstract	1
		(*b*) Provide in the abstract an informative and balanced summary of what was done and what was found	3
**Introduction**			
Background/rationale	2	Explain the scientific background and rationale for the investigation being reported	6-7
Objectives	3	State specific objectives, including any prespecified hypotheses	6-9
**Methods**			
Study design	4	Present key elements of study design early in the paper	8-10
Setting	5	Describe the setting, locations, and relevant dates, including periods of recruitment, exposure, follow-up, and data collection	8
Participants	6	(*a*) *Cohort study*—Give the eligibility criteria, and the sources and methods of selection of participants. Describe methods of follow-up<br>*Case-control study*—Give the eligibility criteria, and the sources and methods of case ascertainment and control selection. Give the rationale for the choice of cases and controls<br>*Cross-sectional study*—Give the eligibility criteria, and the sources and methods of selection of participants	8
		(*b*) *Cohort study*—For matched studies, give matching criteria and number of exposed and unexposed<br>*Case-control study*—For matched studies, give matching criteria and the number of controls per case	NA
Variables	7	Clearly define all outcomes, exposures, predictors, potential confounders, and effect modifiers. Give diagnostic criteria, if applicable	8-10
Data sources/ measurement	8*	For each variable of interest, give sources of data and details of methods of assessment (measurement). Describe comparability of assessment methods if there is more than one group	8-9
Bias	9	Describe any efforts to address potential sources of bias	9-10
Study size	10	Explain how the study size was arrived at	8
Quantitative variables	11	Explain how quantitative variables were handled in the analyses. If applicable, describe which groupings were chosen and why	8-10
Statistical methods	12	(*a*) Describe all statistical methods, including those used to control for confounding	8-11
		(*b*) Describe any methods used to examine subgroups and interactions	8-11
		(*c*) Explain how missing data were addressed	9
		(*d*) *Cohort study*—If applicable, explain how loss to follow-up was addressed<br>*Case-control study*—If applicable, explain how matching of cases and controls was addressed	8-10

		Cross-sectional study—If applicable, describe analytical methods taking account of sampling strategy	
		(*e*) Describe any sensitivity analyses	NA
**Results**			
Participants	13*	(a) Report numbers of individuals at each stage of study—eg numbers potentially eligible, examined for eligibility, confirmed eligible, included in the study, completing follow-up, and analysed	8 (ESM Fig 2.1)
		(b) Give reasons for non-participation at each stage	ESM Fig 2.1
		(c) Consider use of a flow diagram	ESM Fig 2.1
Descriptive data	14*	(a) Give characteristics of study participants (eg demographic, clinical, social) and information on exposures and potential confounders	12 (Table 1 and ESM Fig 4.1–4.4)
		(b) Indicate number of participants with missing data for each variable of interest	9 (ESM Table 2.3)
		(c) *Cohort study*—Summarise follow-up time (eg, average and total amount)	8
Outcome data	15*	*Cohort study*—Report numbers of outcome events or summary measures over time	12-15
		Case-control study—Report numbers in each exposure category, or summary measures of exposure	NA
		Cross-sectional study—Report numbers of outcome events or summary measures	NA
Main results	16	(*a*) Give unadjusted estimates and, if applicable, confounder-adjusted estimates and their precision (eg, 95% confidence interval). Make clear which confounders were adjusted for and why they were included	12-15
		(*b*) Report category boundaries when continuous variables were categorized	NA
		(*c*) If relevant, consider translating estimates of relative risk into absolute risk for a meaningful time period	NA
Other analyses	17	Report other analyses done—eg analyses of subgroups and interactions, and sensitivity analyses	12-15
**Discussion**			
Key results	18	Summarise key results with reference to study objectives	16-19
Limitations	19	Discuss limitations of the study, taking into account sources of potential bias or imprecision. Discuss both direction and magnitude of any potential bias	18
Interpretation	20	Give a cautious overall interpretation of results considering objectives, limitations, multiplicity of analyses, results from similar studies, and other relevant evidence	16-19
Generalisability	21	Discuss the generalisability (external validity) of the study results	16-19
**Other information**			
Funding	22	Give the source of funding and the role of the funders for the present study and, if applicable, for the original study on which the present article is based	20

NA, not applicable

*Give information separately for cases and controls in case-control studies and, if applicable, for exposed and unexposed groups in cohort and cross-sectional studies.

Note: An Explanation and Elaboration article discusses each checklist item and gives methodological background and published examples of transparent reporting. The STROBE checklist is best used in conjunction with this article (freely available on the Web sites of PLoS Medicine at http://www.plosmedicine.org/, Annals of Internal Medicine at http://www.annals.org/, and Epidemiology at http://www.epidem.com/). Information on the STROBE Initiative is available at www.strobe-statement.org.

ESM Appendix 2 Clinical data measurement

Measurements in Hoorn Diabetes Care System (DCS) were measured after the individual had fasted in DCS [1]. HbA_{1c} was measured by turbidimetric inhibition immunoassay of hemolyzed whole EDTA blood [1]. Total cholesterol and HDL cholesterol levels were measured enzymatically (Cobas c501; Roche Diagnostics, Mannheim, Germany), and LDL cholesterol levels were calculated [1].

Measurements in Genetics of Diabetes Audit and Research in Tayside Scotland (GoDARTS) were measured in a non-fasted state [1]. HbA_{1c} was measured in Clinical Pathology Accreditation-accredited National Health Service (UK) laboratories by the International Federation of Clinical Chemistry and Laboratory Medicine standard [2].

In both DCS and GoDARTS, C-peptide was measured on a DiaSorin Liaison [1]. In the current study it was assumed to be constant over time, given its stability and that both cohorts only measured C-peptide once [3].

Cardiovascular diseases were coded based on the International Classification of Diseases, Injuries and Causes of Death, ninth revision (ICD9) in the DCS by self-report (which was partly verified by the electronic individual records from the regional hospital and GP [4]), and tenth revision (ICD10) in the GoDARTS by electronic record linkage to individuals' medical records [5].

Information on treatment was classified using the Anatomical Therapeutic Chemical (ATC) classification codes. This was obtained in the DCS cohort by annually registered treatment information in Hoorn from the dispensing labels of medication [4], and in the GoDARTS cohort by the electronic record linkage to individuals' prescription information [5].

ESM Table 2.1 Definitions of outcomes

Abbreviation	Full version	Definition
**CHF**	Congestive heart failure	ICD 9-428 or ICD 10-I50
**Stroke**	Hemorrhagic stroke and ischemic stroke	ICD 9-430-438 or ICD 10-I61, I63
**AMI**	Acute myocardial infarction	ICD 9-410 or ICD 10-I21
**PVD**	Peripheral vascular disease	ICD 9-443 or ICD 10-I73
**CKD**	Chronic kidney disease	eGFR < 60 L/min/1.73 m2 (eGFR is computed by CKD-EPI equation [6, 7])
**ESRD**	End-stage renal disease	eGFR < 15 L/min /1.73 m^2 (eGFR is computed by CKD-EPI equation [6, 7])

ESM Table 2.2 Treatment steps used for classification of medication use

	**CVD treatment (ATC: C01–C10)**	**Metformin or glinides (ATC: A10BA02 [metformin], A10BX [repaglinid and nateglinide])**	**Sulfonylurea (ATC: A10BB)**	**Insulin (ATC: A10A)**	**Other oral antidiabetic drugs (OAD) (ATC: A10BH [dipeptidyl peptidase-4 inhibitors], A10BJ [glucagon-like peptide-1], A10BX07 [liraglutide], A10BF [alpha glucosidase inhibitors], A10BK [sodium-glucose cotransporter-2 inhibitors], A10BX09 [dapagliflozin], A10BG [thiazolidinediones])**
**Step 0a: No common treatment (diet and exercise)**	No	No	No	No	No
**Step 0b: Only CVD treatment**	Yes	No	No	No	No
**Step 1**	Yes or No	Yes	No	No	No
**Step 2**	Yes or No	Yes or No	Yes	No	No
**Step 3**	Yes or No	Yes or No	Yes or No	Yes	No
**Other OAD**	Yes or No	Yes or No	Yes or No	Yes or No	Yes

ESM Table 2.3 The number and proportion of missing data

	**DCS**		**Individual-level annual average measures of GoDARTS[1]**		**Original data of GoDARTS**	
	Number of missing	Proportion of missing[2]	Number of missing	Proportion of missing[2]	Number of missing	Proportion of missing[2]
**Diabetes duration**	0	0.00%	0	0.00%	0	0.00%
**Cardiovascular disease treatment (e.g. lipid and blood pressure control treatment)**	31	0.08%	0	0.00%	0	0.00%
**Metformin**	79	0.21%	0	0.00%	0	0.00%
**Sulfonylureas**	79	0.21%	0	0.00%	0	0.00%
**Insulin**	79	0.21%	0	0.00%	0	0.00%
**Dipeptidyl peptidase-4 inhibitors**	79	0.21%	0	0.00%	0	0.00%
**Glucagon-like peptide-1 analogues**	79	0.21%	0	0.00%	0	0.00%
**Alpha glucosidase inhibitors**	79	0.21%	0	0.00%	0	0.00%
**Sodium-glucose cotransporter-2 inhibitors**	79	0.21%	0	0.00%	0	0.00%
**Thiazolidinediones**	79	0.21%	0	0.00%	0	0.00%
**Other diabetes treatment (e.g. meglitinides)**	79	0.21%	0	0.00%	0	0.00%
**BMI**	257	0.70%	7138	8.75%	342876	66.04%
**Systolic BP**	310	0.84%	12933	15.85%	298176	57.43%
**Diastolic BP**	313	0.85%	12937	15.86%	298118	57.42%
**Total cholesterol**	375	1.02%	7391	9.06%	373575	71.95%
**Triglyceride**	555	1.51%	35320	43.29%	450645	86.80%
**Blood Creatinine**	527	1.43%	3760	4.61%	208056	40.07%
**HDL-cholesterol**	404	1.10%	7734	9.48%	374914	72.21%
**HbA_{1c}, %**	439	1.19%	6803	8.34%	347307	66.89%
**HbA_{1c}, mmol**	435	1.18%	6812	8.35%	347385	66.91%
**LDL-cholesterol**	604	1.64%	37935	46.50%	457040	88.03%

DCS, Hoorn Diabetes Care System (Netherlands); GoDARTS, Genetics of Diabetes Audit and Research in Tayside Scotland (Scotland)

[1] DCS was measured annually, but GoDARTS was linked to electronic heath records and thus not on a regular annual basis, we therefore took annual average for each measurement for each individual.

[2] Proportion of missing data to all observations

ESM Figure 2.1 Data selection process flowchart

DCS

Raw data (DCS_1998_2019_28042020e)
15392 patients
118705 observations
2583643 records

↓

Merge with C-peptide data
4442 patients
54029 observations
1234938 records

↓

Exclude observations other than Type 2 Diabetes
4390 patients
53403 observations
1220643 records

↓

Remove outliers+ in clustering indicators
4390 patients
53403 observations
1219938 records

↓

Exclude observations with more than 3 years' deviations in age at diagnosis and (current age-diabetes duration)*
4384 patients
53284 observations
1217224 records

↓

Exclude observations with missing diabetes duration and age at diagnosis at the same time
4376 patients
53226 observations
1216063 records

↓

Exclude observations age of diagnosis less than 35
4268 patients
51822 observations
1184370 records

↓

Exclude observations which are not complete in clustering indicators within 2 years of diagnosis
3054 patients
36821 observations
841922 records

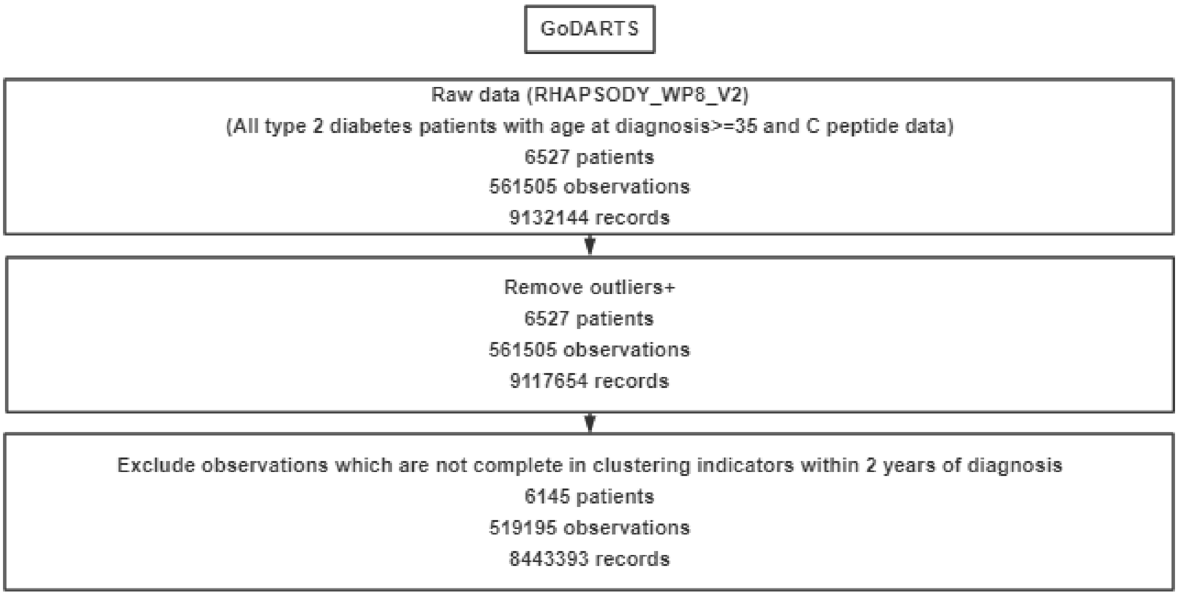

DCS, Hoorn Diabetes Care System (Netherlands); GoDARTS, Genetics of Diabetes Audit and Research in Tayside Scotland (Scotland)

* The adjusting method is listed in **ESM Fig.2.2.**

+ Extreme outliers are defined as values above the upper quantile + 4.5*interquartile range or the lower quantile – 4.5*interquartile range.

For each individual, we had multiple observations over time and each observation had risk factor values and medication use, including diabetes duration, cardiovascular disease treatment (e.g. lipid and blood pressure control treatment), metformin, sulfonylureas, insulin, dipeptidyl peptidase-4 inhibitors, glucagon-like peptide-1, alpha glucosidase inhibitors, sodium-glucose cotransporter-2 inhibitors, thiazolidinediones, other diabetes treatment (e.g. meglitinides), BMI, Systolic BP, Diastolic BP, total cholesterol, triglyceride, blood creatinine, HDL-cholesterol, HbA_{1c} in %, HbA_{1c} in mmol/mol, LDL-cholesterol, C peptide and age at diagnosis, and each of them accounts for a record.

ESM Figure 2.2 The process chart of adjusting diabetes duration of DCS

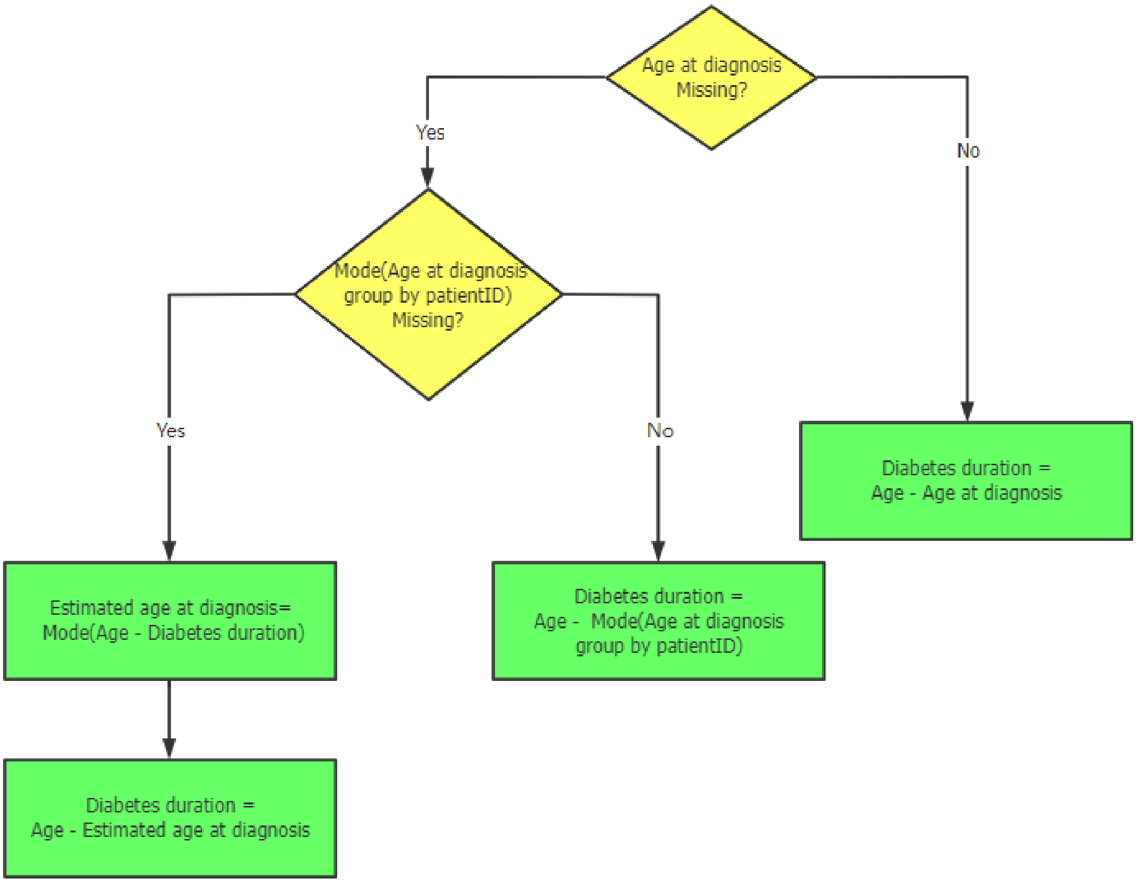

For individuals with missing diabetes durations, we imputed their diabetes durations by their current age of each observation and age at diagnosis of diabetes.

ESM Appendix 3 R packages applied in the analysis

Figures were produced using the R packages *ggplot2 3.3.5*, *plotly 4.10.0* and *ggstatsplot 0.9.0* [8-10]. Clusters were defined by *scale* and *kmeans* functions [11]. The stability of the clusters was assessed using the package *caret 6.0.88* [12]. Differences among subgroups were investigated by the package *lme4 1.1.27.1* for random intercept models [13], *survminer 0.4.9* and *survival 3.2.13* for complications analysis [14, 15], and *VGAM 1.1.5* for multinomial logistic regressions of treatment patterns [16].

ESM Appendix 4 Baseline characteristics and the progression of clinical parameters over time

ESM Table 4.1 The overall statistics of comparing subgroups in this study to subgroups identified in a previous study [1]

Overall statistics	
**Accuracy (95% CI)**	0.92 (0.91-0.93)
**P-Value [Acc > NIR]**	<0.0001
**Kappa**	0.90
**Average sensitivity**	0.92
**Average specificity**	0.98
**Average specific agreement**	0.92

ESM Table 4.2 Scaled clustering centers of subgroups at baseline

DCS										
	**Female**					**Male**				
Subgroup	HDL-cholesterol	HbA_{1c}	BMI	C-peptide	Age at diagnosis	HDL-cholesterol	HbA_{1c}	BMI	C-peptide	Age at diagnosis
RHAP-SIDD	-0.2	2.27	-0.3	-0.51	-0.22	-0.12	2.07	-0.19	-0.42	-0.23
RHAP-SIRD	-0.42	-0.28	0.24	1.39	0.66	-0.28	-0.36	-0.15	0.57	0.89
RHAP-MOD	-0.38	-0.13	1.33	0.25	-0.95	-0.56	0.05	1.62	0.99	-0.64
RHAP-MD	-0.36	-0.3	-0.44	-0.4	-0.13	-0.32	-0.33	-0.15	-0.41	-0.71
RHAP-MDH	1.27	-0.4	-0.57	-0.51	0.58	1.31	-0.42	-0.67	-0.58	0.55
GoDARTS										
	**Female**					**Male**				
Subgroup	HDL-cholesterol	HbA_{1c}	BMI	C-peptide	Age at diagnosis	HDL-cholesterol	HbA_{1c}	BMI	C-peptide	Age at diagnosis
RHAP-SIDD	-0.16	1.77	-0.31	-0.25	-0.07	-0.16	1.53	-0.39	-0.34	-0.36
RHAP-SIRD	-0.32	-0.4	-0.12	1.29	0.59	-0.25	-0.3	0.09	1.6	0.6
RHAP-MOD	-0.4	-0.05	1.57	0.13	-0.99	-0.38	-0.09	1.25	-0.08	-0.95
RHAP-MD	-0.39	-0.39	-0.19	-0.53	-0.53	-0.29	-0.56	-0.37	-0.4	0.24
RHAP-MDH	1.07	-0.45	-0.59	-0.42	0.83	1.62	-0.38	-0.56	-0.31	0.65

DCS, Hoorn Diabetes Care System (Netherlands); GoDARTS, Genetics of Diabetes Audit and Research in Tayside Scotland (Scotland)

ESM Table 4.3 Random intercept models of clinical parameters

		**BMI**	**HbA_{1c}**	**HDL-cholesterol**	**Systolic BP**	**Diastolic BP**	**Total cholesterol**	**LDL-cholesterol**	**Blood creatinine**	**Triglycerides**
**DCS**	Intercept	30.518 ***	56.155 ***	3.015 ***	1.327 ***	141.349 ***	79.535 ***	5.162 ***	67.896 ***	1.799 ***
	Diabetes duration	-0.047 ***	0.268 ***	-0.059 ***	0.003 ***	0.164 ***	-0.289 ***	-0.063 ***	0.624 ***	-0.013 ***
	Male	-1.320 ***	0.922 ***	-0.222 ***	-0.208 ***	-2.198 ***	1.608 ***	-0.432 ***	15.847 ***	0.005
	RHAP-SIRD	0.520 *	-9.649 ***	-0.185 ***	-0.084 ***	3.642 ***	-1.651 ***	-0.203 ***	10.763 ***	0.163 ***
	RHAP-MOD	7.917 ***	-6.324 ***	-0.079 .	-0.110 ***	-3.122 **	2.535 ***	-0.052	-0.648	0.333 ***
	RHAP-MD	-0.779 **	-7.736 ***	0.039	-0.069 ***	-3.196 ***	0.398	0.018	-1.264	0.108 *
	RHAP-MDH	-2.334 ***	-10.562 ***	-0.003	0.361 ***	3.354 ***	-1.370 ***	0.180 ***	2.451 *	-0.391 ***
**GoDARTS**	Intercept	31.770 ***	62.811 ***	2.660 ***	1.313 ***	142.607 ***	81.254 ***	5.090 ***	63.684 ***	2.441 ***
	Diabetes duration	-0.066 ***	0.304 ***	-0.058 ***	-0.005 ***	-0.439 ***	-0.702 ***	-0.086 ***	1.486 ***	-0.043 ***
	Male	-1.936 ***	-0.686 **	-0.253 ***	-0.133 ***	-1.831 ***	0.025	-0.412 ***	15.803 ***	-0.070 **
	RHAP-SIRD	0.637 **	-9.846 ***	-0.154 ***	-0.082 ***	-0.484	-3.439 ***	-0.182 ***	14.535 ***	0.168 ***
	RHAP-MOD	8.598 ***	-2.550 ***	0.088 **	-0.087 ***	-0.811 .	3.019 ***	0.124 ***	-6.050 ***	0.280 ***
	RHAP-MD	-0.657 ***	-8.934 ***	0.087 ***	-0.036 ***	-1.450 ***	-0.682 **	0.025	-0.643	0.003
	RHAP-MDH	-2.716 ***	-11.878 ***	-0.065 *	0.372 ***	-0.050	-3.166 ***	0.032	3.897 ***	-0.576 ***

DCS, Hoorn Diabetes Care System (Netherlands); GoDARTS, Genetics of Diabetes Audit and Research in Tayside Scotland (Scotland); SIRD, Severe Insulin Resistance Diabetes; MOD, Mild Obesity-related Diabetes; MD, Mild Diabetes; MDH, Mild Diabetes with high HDL-cholesterol
Significance stars are denoted as 0-0.001, “***”, 0.001-0.01, “**”, 0.01-0.05, “*”, 0.05-0.1, “.”

ESM Figure 4.1 Violin plots and pairwise comparisons of clustering indicators at baseline between subgroups of DCS

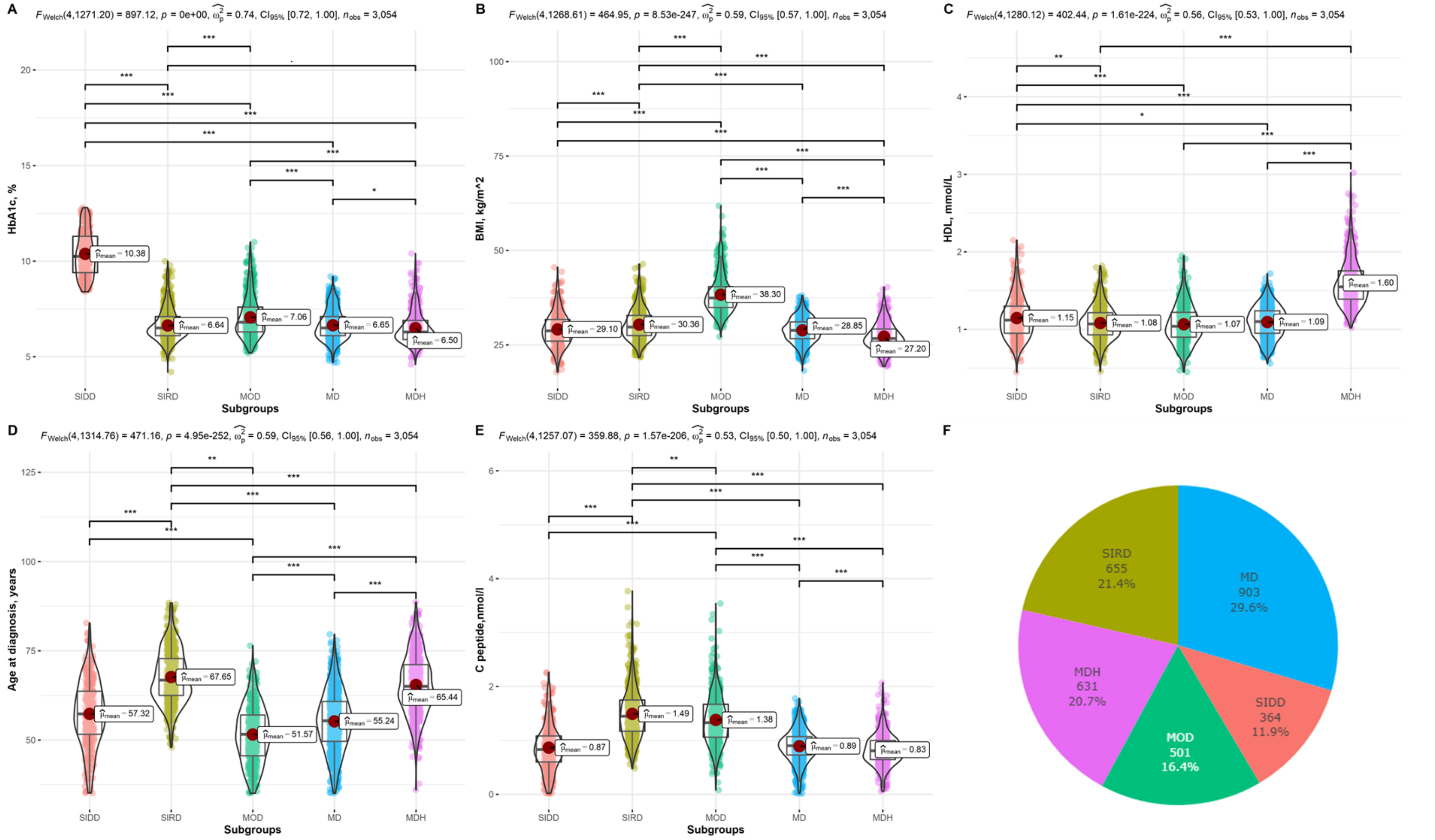

Here, SIDD, SIRD, MOD, MD and MDH refer to RHAP-SIDD, RHAP-SIRD, RHAP-MOD, RHAP-MD and RHAP-MDH, respectively.
DCS, Hoorn Diabetes Care System (Netherlands); SIDD, Severe Insulin Deficiency Diabetes; SIRD, Severe Insulin Resistance Diabetes; MOD, Mild Obesity-related Diabetes; MD, Mild Diabetes; MDH, Mild Diabetes with high HDL-cholesterol; HDL, HDL-cholesterol
Significance stars are denoted as 0-0.001, "***", 0.001-0.01, "**", 0.01-0.05, "*", 0.05-0.1, "."

ESM Figure 4.2 Violin plots and pairwise comparisons of clustering indicators at baseline between subgroups of GoDARTS

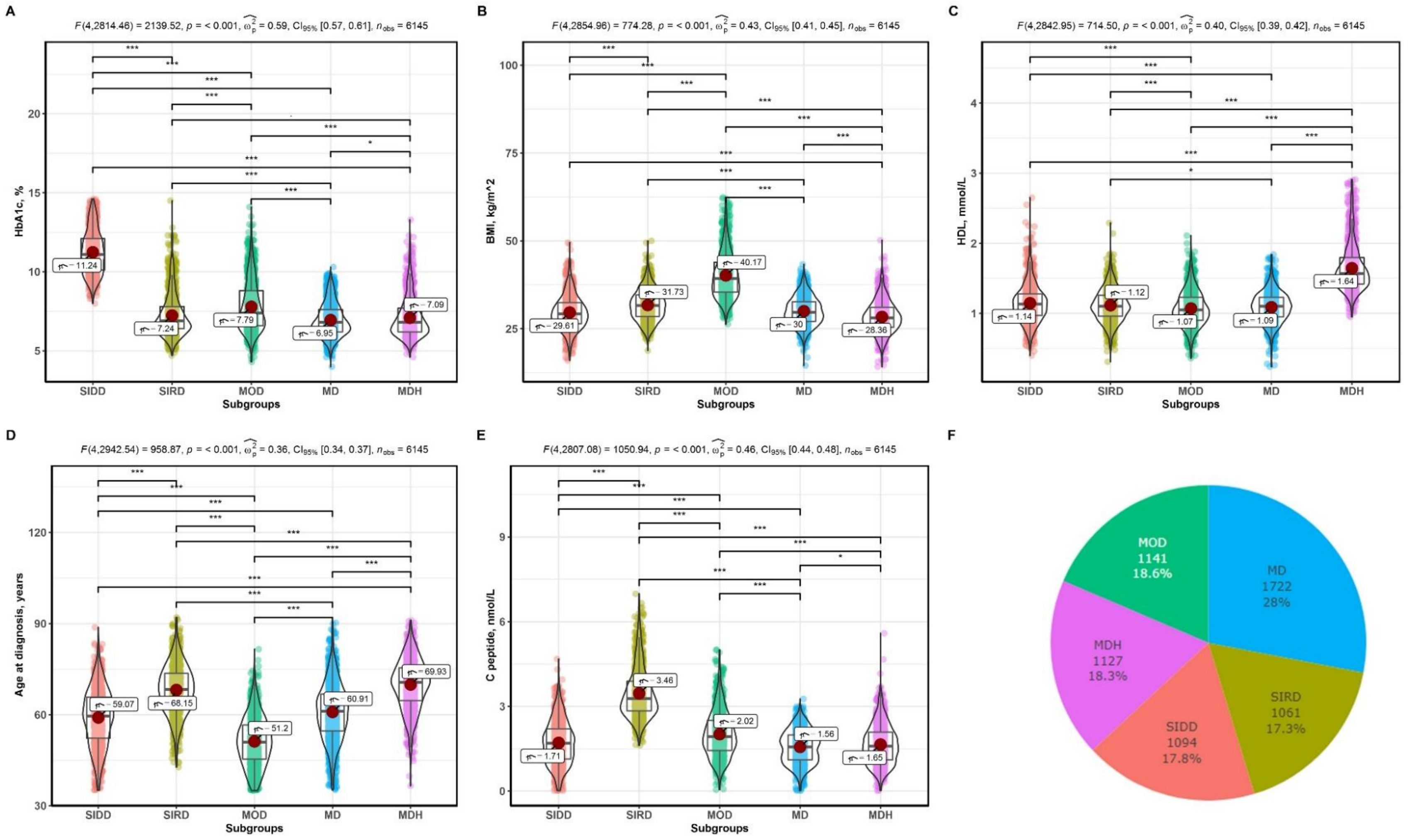

Here, SIDD, SIRD, MOD, MD and MDH refer to RHAP-SIDD, RHAP-SIRD, RHAP-MOD, RHAP-MD and RHAP-MDH, respectively.
GoDARTS, Genetics of Diabetes Audit and Research in Tayside Scotland (Scotland); SIDD, Severe Insulin Deficiency Diabetes; SIRD, Severe Insulin Resistance Diabetes; MOD, Mild Obesity-related Diabetes; MD, Mild Diabetes; MDH, Mild Diabetes with high HDL-cholesterol; HDL, HDL-cholesterol
Significance stars are denoted as 0-0.001, "***", 0.001-0.01, "**", 0.01-0.05, "*", 0.05-0.1, "."

ESM Figure 4.3 Violin plots and pairwise comparisons of other risk factors at baseline between subgroups of DCS

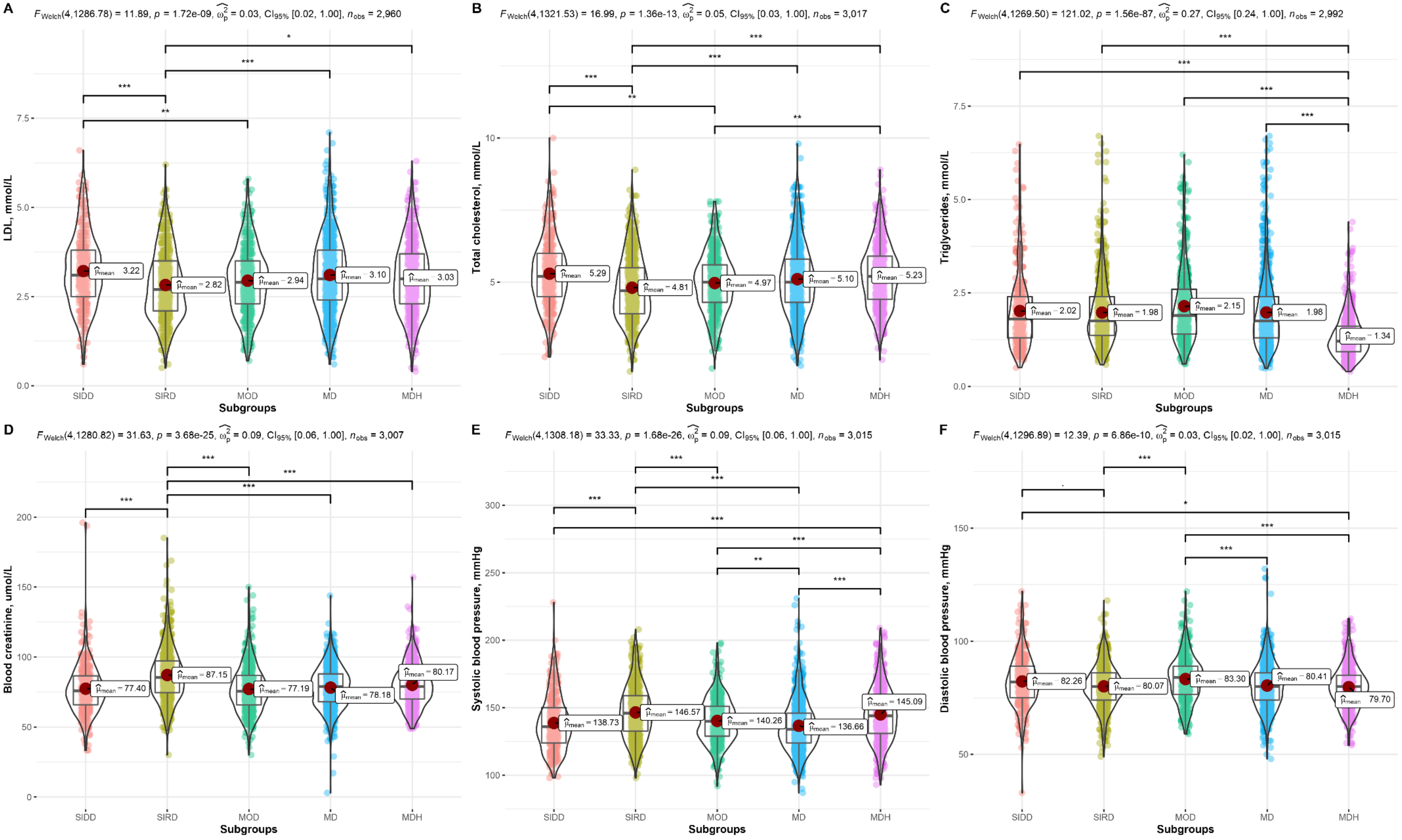

Here, SIDD, SIRD, MOD, MD and MDH refer to RHAP-SIDD, RHAP-SIRD, RHAP-MOD, RHAP-MD and RHAP-MDH, respectively.

DCS, Hoorn Diabetes Care System (Netherlands); SIDD, Severe Insulin Deficiency Diabetes; SIRD, Severe Insulin Resistance Diabetes; MOD, Mild Obesity-related Diabetes; MD, Mild Diabetes; MDH, Mild Diabetes with high HDL-cholesterol; LDL, LDL-cholesterol

Significance stars are denoted as 0-0.001, "***", 0.001-0.01, "**", 0.01-0.05, "*", 0.05-0.1, "."

ESM Figure 4.4 Violin plots and pairwise comparisons of other risk factors at baseline between subgroups of GoDARTS

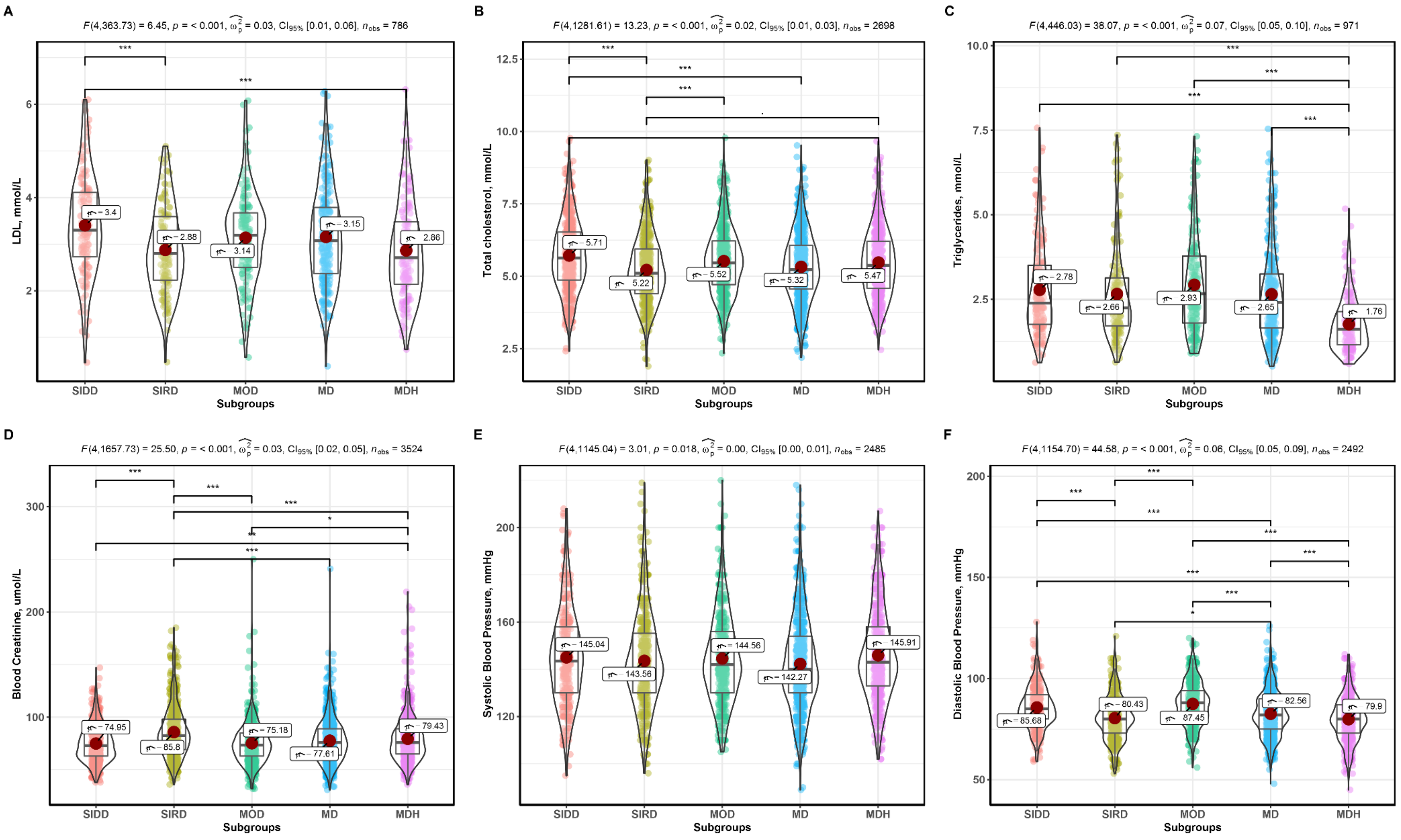

Here, SIDD, SIRD, MOD, MD and MDH refer to RHAP-SIDD, RHAP-SIRD, RHAP-MOD, RHAP-MD and RHAP-MDH, respectively.
GoDARTS, Genetics of Diabetes Audit and Research in Tayside Scotland (Scotland); SIDD, Severe Insulin Deficiency Diabetes; SIRD, Severe Insulin Resistance Diabetes; MOD, Mild Obesity-related Diabetes; MD, Mild Diabetes; MDH, Mild Diabetes with high HDL-cholesterol; LDL, LDL-cholesterol
Significance stars are denoted as 0-0.001, "***", 0.001-0.01, "**", 0.01-0.05, "*", 0.05-0.1, "."

ESM Figure 4.5 Progression of clinical parameters over time based on subgroups identified at diagnosis in the DCS cohort

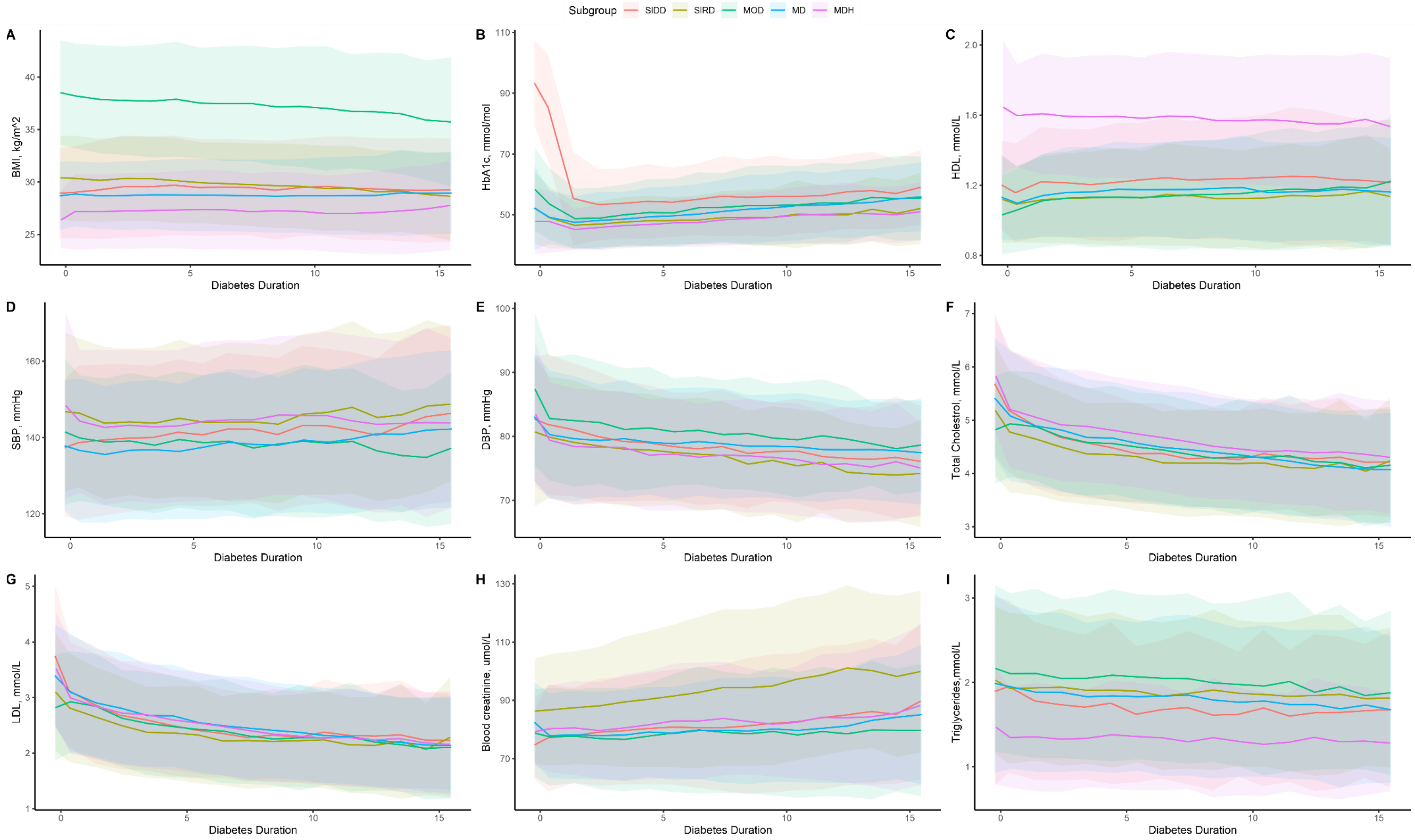

Here, SIDD, SIRD, MOD, MD and MDH refer to RHAP-SIDD, RHAP-SIRD, RHAP-MOD, RHAP-MD and RHAP-MDH, respectively.

DCS, Hoorn Diabetes Care System (Netherlands); SIDD, Severe Insulin Deficiency Diabetes; SIRD, Severe Insulin Resistance Diabetes; MOD, Mild Obesity-related Diabetes; MD, Mild Diabetes; MDH, Mild Diabetes with high HDL-cholesterol; HDL, HDL-cholesterol; SBP, Systolic Blood Pressure; DBP, Diastolic Blood Pressure; LDL, LDL-cholesterol

Values of selected parameters over time in each cluster are shown. The data are represented as mean values (solid line) ±SD (shaded areas). Missing values were removed

ESM Appendix 5 Diabetes-related complications by subgroups

ESM Table 5.1 Results of Schoenfeld tests checking the proportional hazards assumption

DCS																		
	**AMI**			**CHF**			**PVD**			**Stroke**			**CKD**			**ESRD**		
	**chisq**	**df**	**p**	**chisq**	**df**	**p**	**chisq**	**df**	**p**	**chisq**	**df**	**P**	**chisq**	**df**	**p**	**chisq**	**df**	**p**
**Model with subgroups**	2.572	4	0.63	8.962	4	0.062	3.608	4	0.46	5.841	4	0.21	9.407	4	0.052	3.234	4	0.52
**Model with subgroups, age and sex**	6.44	6	0.38	17.948	6	0.006	5.216	6	0.52	6.293	6	0.39	17.745	6	0.007	4.894	6	0.56
**Model with clustering indicators and sex**	7.174	6	0.31	25.863	6	<0.0001	7.017	6	0.32	6.981	6	0.32	22.275	6	0.0001	4.675	6	0.59
**Model with subgroups, clustering indicators, and sex**	8.94	10	0.54	34.484	10	<0.0001	10.654	10	0.39	9.542	10	0.48	27.978	10	0.0002	6.378	10	0.78
**GoDARTS**																		
	**AMI**			**CHF**			**PVD**			**Stroke**			**CKD**			**ESRD**		
	**chisq**	**df**	**p**	**chisq**	**df**	**p**	**chisq**	**df**	**p**	**chisq**	**df**	**P**	**chisq**	**df**	**p**	**chisq**	**df**	**p**
**Model with subgroups**	6.859	4	0.14	10.271	4	0.036	6.736	4	0.15	3.672	4	0.45	47.751	4	<0.0001	1.816	4	0.769
**Model with subgroups, age and sex**	20.621	6	0.0021	11.12	6	0.085	10.659	6	0.1	2.47	6	0.87	68.195	6	<0.0001	5.966	6	0.427
**Model with clustering indicators and sex**	22.592	6	0.00095	7.646	6	0.27	9.06	6	0.17	2.285	6	0.89	75.322	6	<0.0001	5.652	6	0.463
**Model with subgroups, clustering indicators, and sex**	29.41	10	0.0011	11.173	10	0.34	12.739	10	0.24	5.03	10	0.89	78.029	10	<0.0001	10.437	10	0.403

DCS, Hoorn Diabetes Care System (Netherlands); GoDARTS, Genetics of Diabetes Audit and Research in Tayside Scotland (Scotland); AMI, Acute Myocardial Infarction; CHF, Congestive Heart Failure; PVD, Peripheral Vascular Diseases; CKD, Chronic Kidney Disease; ESRD, End Stage Renal Disease; chisq, Chi-squared; df, degree of freedom; p, P value

P values less than 0.05 (indicating the violation of the proportional hazard assumption) are highlighted in red.

ESM Table 5.2 Cox regression analysis comparing event rates between subgroups in GoDARTS

	Events	Censored	% events	Hazard ratio (CI95%)	P value	Hazard ratio (CI95%) [1]	P value	Hazard ratio (CI95%) [2]	P value
**Acute Myocardial Infarction (ICD 410)**									
RHAP-SIDD	256	838	23.40%	0.91 (0.77 - 1.09)	0.31	*1.20 (0.99 - 1.44)*	*0.062 .*	1.11 (0.93 - 1.32)	0.26
RHAP-SIRD	354	707	33.36%	1.65 (1.40 - 1.94)	<0.0001 ***	*1.72 (1.46 - 2.02)*	*<0.0001 ****	2.00 (1.69 - 2.35)	<0.0001 ***
RHAP-MOD	234	907	20.51%	0.82 (0.69 - 0.99)	0.036 *	*1.38 (1.12 - 1.71)*	*0.0030 ***	Reference	
RHAP-MD	402	1320	23.34%	0.99 (0.84 - 1.16)	0.90	*1.22 (1.03 - 1.44)*	*0.022 **	1.20 (1.02 - 1.41)	0.026 *
RHAP-MDH	240	887	21.30%	Reference		*Reference*		1.21 (1.01 - 1.45)	0.036 *
**Congestive Heart Failure (ICD 428)**									
RHAP-SIDD	101	993	9.23%	*0.81 (0.61 - 1.07)*	*0.14*	1.53 (1.14 - 2.06)	0.0046 **	*1.46 (1.08 - 1.98)*	*0.014 **
RHAP-SIRD	144	917	13.57%	*1.55 (1.20 - 2.01)*	*0.00085 ****	1.77 (1.36 - 2.29)	<0.0001 ***	*2.79 (2.10 - 3.71)*	*<0.0001 ****
RHAP-MOD	71	1070	6.22%	*0.55 (0.41 - 0.75)*	*0.00017 ****	1.90 (1.33 - 2.73)	0.00048 ***	*Reference*	
RHAP-MD	143	1579	8.30%	*0.78 (0.60 - 1.00)*	*0.054 .*	1.34 (1.02 - 1.77)	0.037 *	*1.40 (1.05 - 1.86)*	*0.021 **
RHAP-MDH	97	1030	8.61%	*Reference*		Reference		*1.80 (1.33 - 2.45)*	*0.00017 ****
**Peripheral Vascular Disease (ICD 443)**									
RHAP-SIDD	10	1084	0.91%	0.81 (0.33 - 1.94)	0.63	1.39 (0.55 - 3.49)	0.49	1.46 (0.56 - 3.84)	0.44
RHAP-SIRD	19	1042	1.79%	1.95 (0.91 - 4.20)	0.088 .	2.11 (0.98 - 4.56)	0.057 .	3.54 (1.48 - 8.43)	0.0043 **
RHAP-MOD	7	1134	0.61%	0.55 (0.21 - 1.45)	0.23	1.61 (0.53 - 4.91)	0.40	Reference	
RHAP-MD	18	1704	1.05%	0.98 (0.45 - 2.13)	0.96	1.44 (0.64 - 3.24)	0.38	1.78 (0.74 - 4.25)	0.20
RHAP-MDH	10	1117	0.89%	Reference		Reference		1.81 (0.69 - 4.78)	0.23
**Stroke (ICD 430-438)**									
RHAP-SIDD	111	983	10.15%	0.62 (0.48 - 0.79)	0.00016 ***	1.10 (0.84 - 1.43)	0.49	1.45 (1.09 - 1.94)	0.011 *
RHAP-SIRD	134	927	12.63%	0.98 (0.77 - 1.24)	0.88	1.12 (0.88 - 1.42)	0.37	2.31 (1.75 - 3.05)	<0.0001 ***
RHAP-MOD	78	1063	6.84%	0.43 (0.32 - 0.56)	<0.0001 ***	1.28 (0.92 - 1.78)	0.15	Reference	
RHAP-MD	170	1552	9.87%	0.65 (0.52 - 0.81)	0.00016 ***	1.08 (0.85 - 1.38)	0.52	1.53 (1.17 - 1.99)	0.0020 **
RHAP-MDH	140	987	12.42%	Reference		Reference		2.35 (1.78 - 3.10)	<0.0001 ***

GoDARTS, Genetics of Diabetes Audit and Research in Tayside Scotland (Scotland); SIDD, Severe Insulin Deficiency Diabetes; SIRD, Severe Insulin Resistance Diabetes; MOD, Mild Obesity-related Diabetes; MD, Mild Diabetes; MDH, Mild Diabetes with high HDL-cholesterol
Significance stars are denoted as 0-0.001, "***", 0.001-0.01, "**", 0.01-0.05, "*", 0.05-0.1, "."
Italic content indicated models did not fulfil proportional hazard assumptions (P<0.05).
[1] Cox regression adjusted for age and sex
[2] Cox regression used the subgroup with lowest hazard ratio as the reference group to capture the difference to the greatest extent

ESM Table 5.3 Cox regression analysis comparing event rates between subgroups in DCS

	Events	Censored	% events	Hazard ratio (CI95%)	P value	Hazard ratio (CI95%) [1]	P value	Hazard ratio (CI95%) [2]	P value
**Acute Myocardial Infarction (ICD 410)**									
RHAP-SIDD	10	354	2.75%	1.70 (0.69 - 4.18)	0.25	2.25 (0.89 - 5.66)	0.085 .	1.70 (0.69 - 4.18)	0.25
RHAP-SIRD	31	624	4.73%	3.53 (1.68 - 7.41)	0.00087 ***	2.86 (1.35 - 6.04)	0.0059 **	3.53 (1.68 - 7.41)	0.00087 ***
RHAP-MOD	8	493	1.60%	1.06 (0.41 - 2.76)	0.90	2.04 (0.74 - 5.65)	0.17	1.06 (0.41 - 2.76)	0.90
RHAP-MD	26	877	2.88%	1.89 (0.89 - 4.04)	0.10 .	3.09 (1.36 - 7.06)	0.0073 **	1.89 (0.89 - 4.04)	0.10 .
RHAP-MDH	9	622	1.43%	Reference		Reference		Reference	
**Congestive Heart Failure (ICD 428)**									
RHAP-SIDD	17	347	5.19%	0.72 (0.40 - 1.30)	0.28	*1.23 (0.68 - 2.25)*	0.49	1.50 (0.74 - 3.04)	0.26
RHAP-SIRD	49	606	6.98%	1.50 (0.97 - 2.33)	0.068 .	*1.24 (0.80 - 1.93)*	0.34	3.12 (1.72 - 5.65)	0.00018 ***
RHAP-MOD	14	487	2.75%	0.48 (0.26 - 0.90)	0.022 *	*1.40 (0.71 - 2.78)*	0.33	Reference	
RHAP-MD	33	870	3.69%	0.62 (0.38 - 1.00)	0.049 *	*1.35 (0.80 - 2.29)*	0.27	1.28 (0.69 - 2.39)	0.44
RHAP-MDH	34	597	5.30%	Reference		*Reference*		2.07 (1.11 - 3.86)	0.022 *
**Peripheral Vascular Disease (ICD 443)**									
RHAP-SIDD	<5	361	0.99%	0.64 (0.17 - 2.48)	0.52	0.78 (0.19 - 3.12)	0.722	1.86 (0.31 - 11.14)	0.50
RHAP-SIRD	8	647	1.43%	1.18 (0.43 - 3.27)	0.75	1.03 (0.37 - 2.88)	0.952	3.44 (0.73 - 16.23)	0.12
RHAP-MOD	<5	499	0.21%	0.34 (0.07 - 1.65)	0.18	0.53 (0.1 - 2.85)	0.459	Reference	
RHAP-MD	12	891	1.15%	1.11 (0.44 - 2.83)	0.82	1.51 (0.53 - 4.32)	0.445	3.24 (0.72 - 14.47)	0.12
RHAP-MDH	7	624	1.25%	Reference		Reference		2.91 (0.6 - 14.01)	0.18
**Stroke (ICD 430-438)**									
RHAP-SIDD	23	341	6.32%	0.96 (0.57 - 1.63)	0.88	1.56 (0.9 - 2.69)	0.11	2.22 (1.13 - 4.39)	0.021 *
RHAP-SIRD	32	623	4.89%	0.93 (0.58 - 1.51)	0.78	0.82 (0.5 - 1.32)	0.41	2.16 (1.13 - 4.12)	0.019 *
RHAP-MOD	13	488	2.59%	0.43 (0.23 - 0.82)	0.01 **	1.08 (0.53 - 2.17)	0.84	Reference	
RHAP-MD	37	866	4.10%	0.67 (0.42 - 1.07)	0.091 .	1.29 (0.78 - 2.14)	0.33	1.55 (0.83 - 2.92)	0.17
RHAP-MDH	35	596	5.55%	Reference		Reference		2.31 (1.22 - 4.38)	0.0098 **

DCS, Hoorn Diabetes Care System (Netherlands); SIDD, Severe Insulin Deficiency Diabetes; SIRD, Severe Insulin Resistance Diabetes; MOD, Mild Obesity-related Diabetes; MD, Mild Diabetes; MDH, Mild Diabetes with high HDL-cholesterol

Significance stars are denoted as 0-0.001, "***", 0.001-0.01, "**", 0.01-0.05, "*", 0.05-0.1, "."

Italic content indicated models did not fulfil proportional hazard assumptions (P<0.05).

[1] Cox regression adjusted for age and sex

[2] Cox regression used the subgroup with lowest hazard ratio as the reference group to capture the difference to the greatest extent

ESM Table 5.4 Pairwise comparisons comparing event rates between subgroups using Log-Rank test in GoDARTS

		**RHAP-SIDD &RHAP-SIRD**	**RHAP-SIDD &RHAP-MOD**	**RHAP-SIDD &RHAP-MD**	**RHAP-SIDD &RHAP-MDH**	**RHAP-SIRD &RHAP-MOD**	**RHAP-SIRD &RHAP-MD**	**RHAP-SIRD& RHAP-MDH**	**RHAP-MOD &RHAP-MD**	**RHAP-MOD &RHAP-MDH**	**RHAP-MD &RHAP-MDH**
**All**	AMI	<0.0001 ***	0.34	0.35	0.34	<0.0001 ***	<0.0001 ***	<0.0001 ***	0.058 .	0.070 .	0.89
	CHF	<0.0001 ***	0.024 *	0.71	0.13	<0.0001 ***	<0.0001 ***	0.0011 **	0.029 *	0.00047 ***	0.039 *
	PVD	0.13	0.61	0.76	0.76	0.029 *	0.13	0.18	0.32	0.32	0.8
	Stroke	0.0006 ***	0.012 *	0.78	0.00031 ***	<0.0001 ***	0.00078 ***	0.92	0.0025 **	<0.0001 ***	0.00063 ***
	CKD	<0.0001 ***	<0.0001 ***	0.42	<0.0001 ***	<0.0001 ***	<0.0001 ***	<0.0001 ***	<0.0001 ***	<0.0001 ***	<0.0001 ***
	ESRD	0.00032 ***	0.21	0.14	0.00041 ***	<0.0001 ***	<0.0001 ***	1	1		<0.0001 ***
**Males**	AMI	<0.0001 ***	0.11	0.11	0.19	<0.0001 ***	<0.0001 ***	<0.0001 ***	0.00081 ***	0.0099 **	0.91
	CHF	0.00035 ***	0.013 *	0.53	0.17	<0.0001 ***	0.00035 ***	0.037 *	0.00091 ***	0.00035 ***	0.25
	PVD	0.63	0.63	0.67	0.65	0.44	0.63	0.44	0.44	0.78	0.63
	Stroke	0.012 *	0.11	0.4	0.0089 **	<0.0001 ***	0.047 *	0.9	0.013 *	<0.0001 ***	0.039 *
	CKD	<0.0001 ***	0.47	0.0025 **	<0.0001 ***	<0.0001 ***	<0.0001 ***	0.45	0.00031 ***	<0.0001 ***	<0.0001 ***
	ESRD	0.00024 ***	0.73	0.59	0.46	0.0012 **	<0.0001 ***	0.0024 **	0.46	0.59	0.16
**Females**	AMI	0.00024 ***	0.73	0.59	0.46	0.0012 **	<0.0001 ***	0.0024 **	0.46	0.59	0.16
	CHF	0.0033 **	0.56	0.25	0.38	0.0013 **	<0.0001 ***	0.023 *	0.56	0.25	0.023 *
	PVD	0.046 *	0.98	0.98	0.11	0.046 *	0.046 *	0.46	0.98	0.11	0.097 .
	Stroke	0.03 *	0.057 .	0.68	0.022 *	0.00023 ***	0.0062 **	0.96	0.093 .	0.00023 ***	0.0062 **
	CKD	<0.0001 ***	<0.0001 ***	<0.0001 ***	<0.0001 ***	<0.0001 ***	<0.0001 ***	<0.0001 ***	0.37	<0.0001 ***	<0.0001 ***
	ESRD	0.0046 **	0.24	0.083 .	0.0091 **	0.0004 ***	<0.0001 ***	0.86	0.86	0.00038 ***	<0.0001 ***

GoDARTS, Genetics of Diabetes Audit and Research in Tayside Scotland (Scotland); SIDD, Severe Insulin Deficiency Diabetes; SIRD, Severe Insulin Resistance Diabetes; MOD, Mild Obesity-related Diabetes; MD, Mild Diabetes; MDH, Mild Diabetes with high HDL-cholesterol; AMI, Acute Myocardial Infarction; CHF, Congestive Heart Failure; PVD, Peripheral Vascular Diseases; CKD, Chronic Kidney Disease; ESRD, End Stage Renal Disease
Significance stars are denoted as 0-0.001, "***", 0.001-0.01, "**", 0.01-0.05, "*", 0.05-0.1, "."

ESM Table 5.5 Pairwise comparisons comparing event rates between subgroups using Log-Rank test in DCS

		**RHAP-SIDD &RHAP-SIRD**	**RHAP-SIDD &RHAP-MOD**	**RHAP-SIDD &RHAP-MD**	**RHAP-SIDD &RHAP-MDH**	**RHAP-SIRD &RHAP-MOD**	**RHAP-SIRD &RHAP-MD**	**RHAP-SIRD& RHAP-MDH**	**RHAP-MOD &RHAP-MD**	**RHAP-MOD &RHAP-MDH**	**RHAP-MD &RHAP-MDH**
**All**	AMI	0.075 .	0.44	0.82	0.44	0.0092 **	0.074 .	0.0039 **	0.24	0.89	0.18
	CHF	0.056 .	0.35	0.55	0.3	0.00072 ***	0.00072 ***	0.11	0.48	0.045 *	0.084 .
	PVD	0.72	0.74	0.72	0.74	0.57	0.9	0.9	0.57	0.57	0.9
	Stroke	0.075 .	0.44	0.82	0.44	0.0092 **	0.074 .	0.0039 **	0.24	0.89	0.18
	CKD	<0.0001 ***	0.13	0.0025 **	0.0033 **	<0.0001 ***	<0.0001 ***	<0.0001 ***	0.13	<0.0001 ***	<0.0001 ***
	ESRD	0.36	0.99	0.36	0.99	0.36	0.032 *	0.36	0.36	0.99	0.36
**Males**	AMI	0.35	0.85	0.83	0.85	0.35	0.35	0.15	0.83	0.83	0.48
	CHF	0.071 .	0.53	0.9	0.23	0.12	0.014 *	0.23	0.56	0.53	0.23
	PVD	0.86	0.27	0.86	0.86	0.2	0.86	0.86	0.2	0.2	0.99
	Stroke	0.79	0.52	0.52	0.84	0.48	0.36	0.75	0.96	0.52	0.52
	CKD	0.79	0.52	0.52	0.84	0.48	0.36	0.75	0.96	0.52	0.52
	ESRD	<0.0001 ***	0.65	<0.0001 ***	0.067 .	<0.0001 ***	<0.0001 ***	<0.0001 ***	<0.0001 ***	0.017 *	<0.0001 ***
**Females**	AMI	0.33	0.11	0.92	0.26	0.028 *	0.26	0.064 .	0.11	0.39	0.26
	CHF	0.66	0.033 *	0.43	0.86	0.012 *	0.12	0.48	0.12	0.021 *	0.43
	PVD	0.85	0.85	0.85	0.85	0.85	0.85	0.85	0.85	0.89	0.85
	Stroke	0.59	0.14	0.59	0.74	0.38	0.8	0.38	0.27	0.056 .	0.38
	CKD	<0.0001 ***	0.071 .	0.77	0.036 *	<0.0001 ***	<0.0001 ***	<0.0001 ***	0.06 .	<0.0001 ***	0.0026 **
	ESRD	0.6	0.6	0.6	0.6	0.6	0.6	0.6	0.94	0.94	0.87

DCS, Hoorn Diabetes Care System (Netherlands); SIDD, Severe Insulin Deficiency Diabetes; SIRD, Severe Insulin Resistance Diabetes; MOD, Mild Obesity-related Diabetes; MD, Mild Diabetes; MDH, Mild Diabetes with high HDL-cholesterol; AMI, Acute Myocardial Infarction; CHF, Congestive Heart Failure; PVD, Peripheral Vascular Diseases; CKD, Chronic Kidney Disease; ESRD, End Stage Renal Disease

Significance stars are denoted as 0-0.001, "***", 0.001-0.01, "**", 0.01-0.05, "*", 0.05-0.1, "."

ESM Figure 5.1 Kaplan Meier plots of GoDARTS

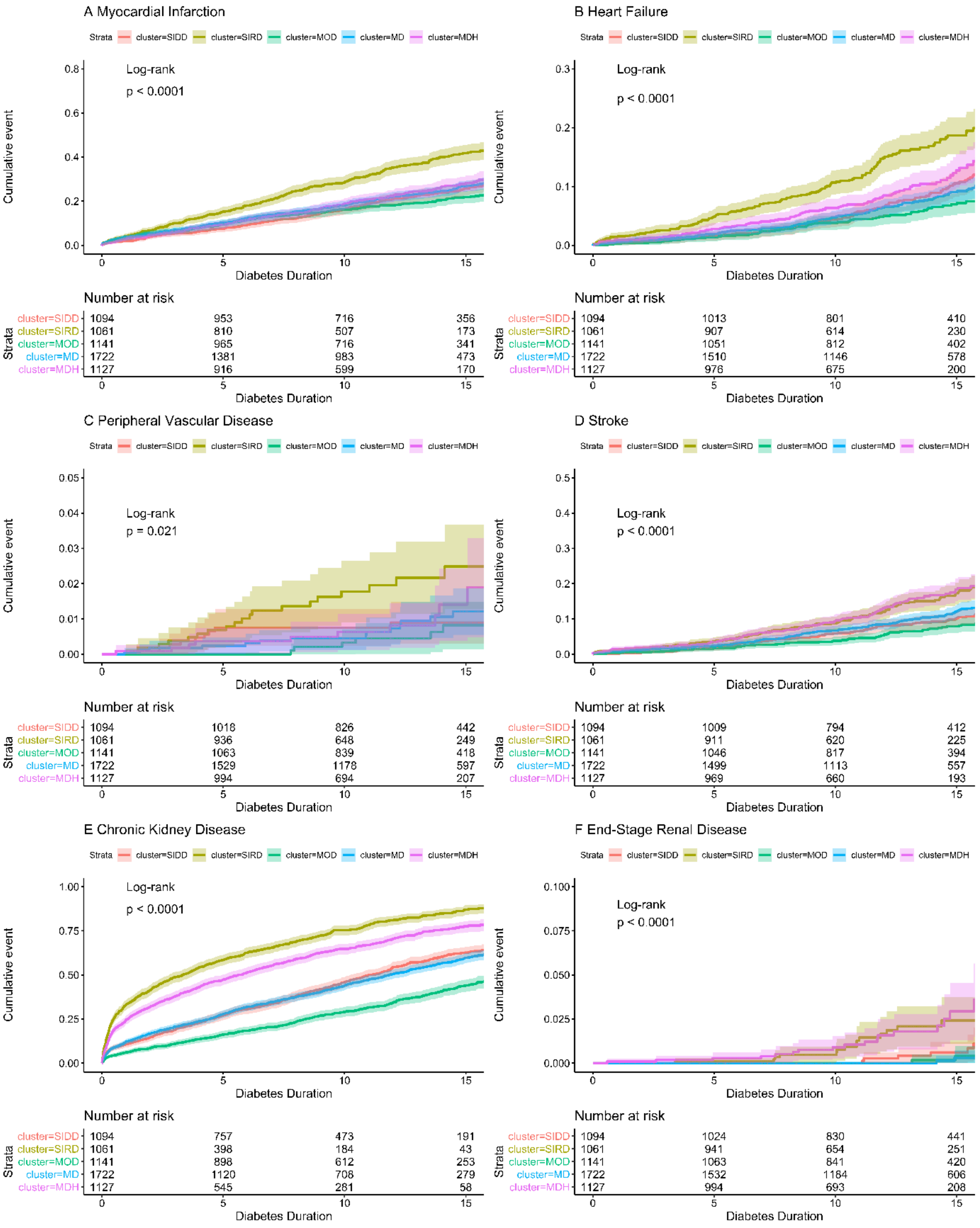

Here, SIDD, SIRD, MOD, MD and MDH refer to RHAP-SIDD, RHAP-SIRD, RHAP-MOD, RHAP-MD and RHAP-MDH, respectively.
GoDARTS, Genetics of Diabetes Audit and Research in Tayside Scotland (Scotland); SIDD, Severe Insulin Deficiency Diabetes; SIRD, Severe Insulin Resistance Diabetes; MOD, Mild Obesity-related Diabetes; MD, Mild Diabetes; MDH, Mild Diabetes with high HDL-cholesterol

ESM Figure 5.2 Kaplan Meier plots of DCS

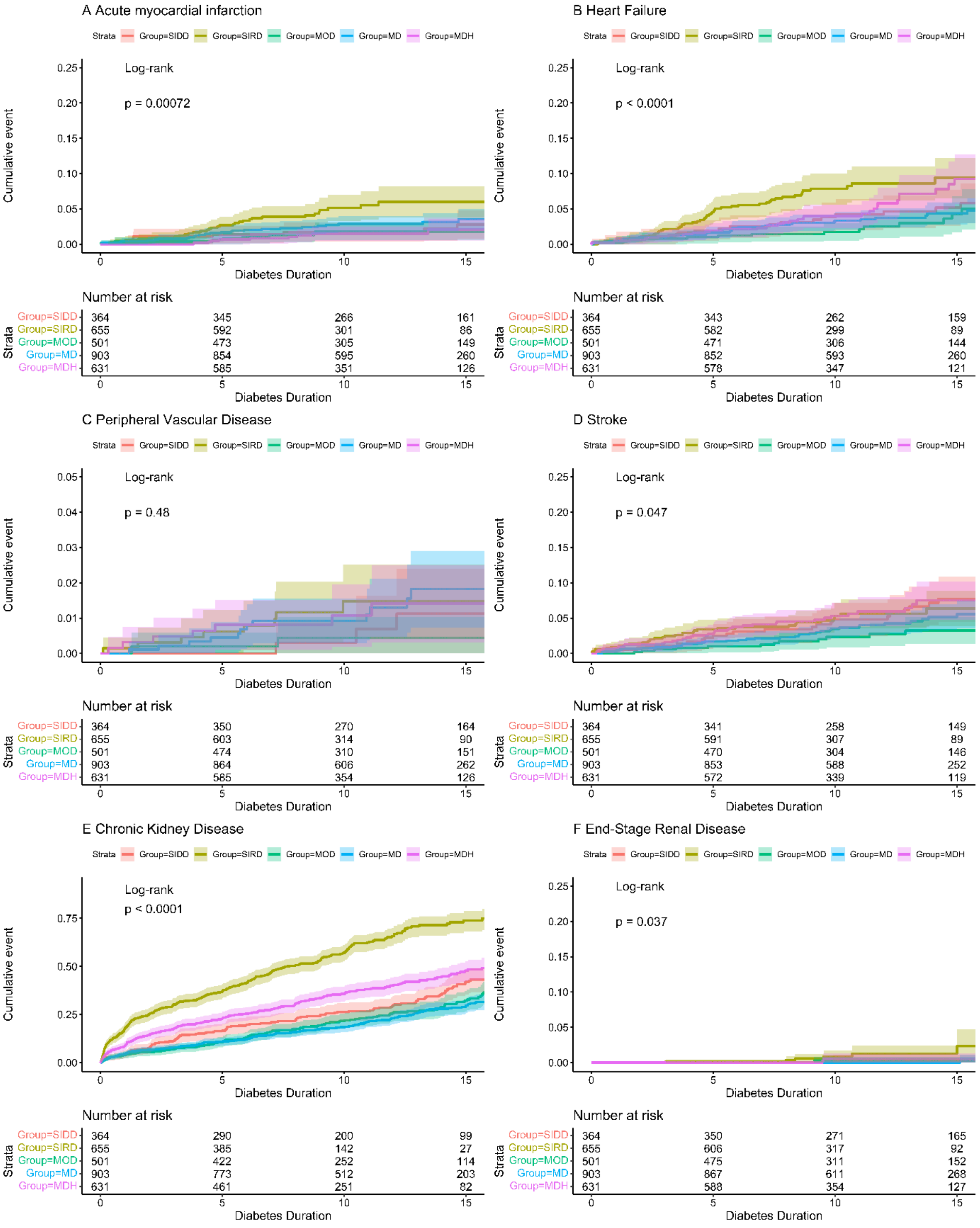

Here, SIDD, SIRD, MOD, MD and MDH refer to RHAP-SIDD, RHAP-SIRD, RHAP-MOD, RHAP-MD and RHAP-MDH, respectively.

DCS, Hoorn Diabetes Care System (Netherlands); SIDD, Severe Insulin Deficiency Diabetes; SIRD, Severe Insulin Resistance Diabetes; MOD, Mild Obesity-related Diabetes; MD, Mild Diabetes; MDH, Mild Diabetes with high HDL-cholesterol

ESM Figure 5.3 The results of Cox regression analysis of DCS

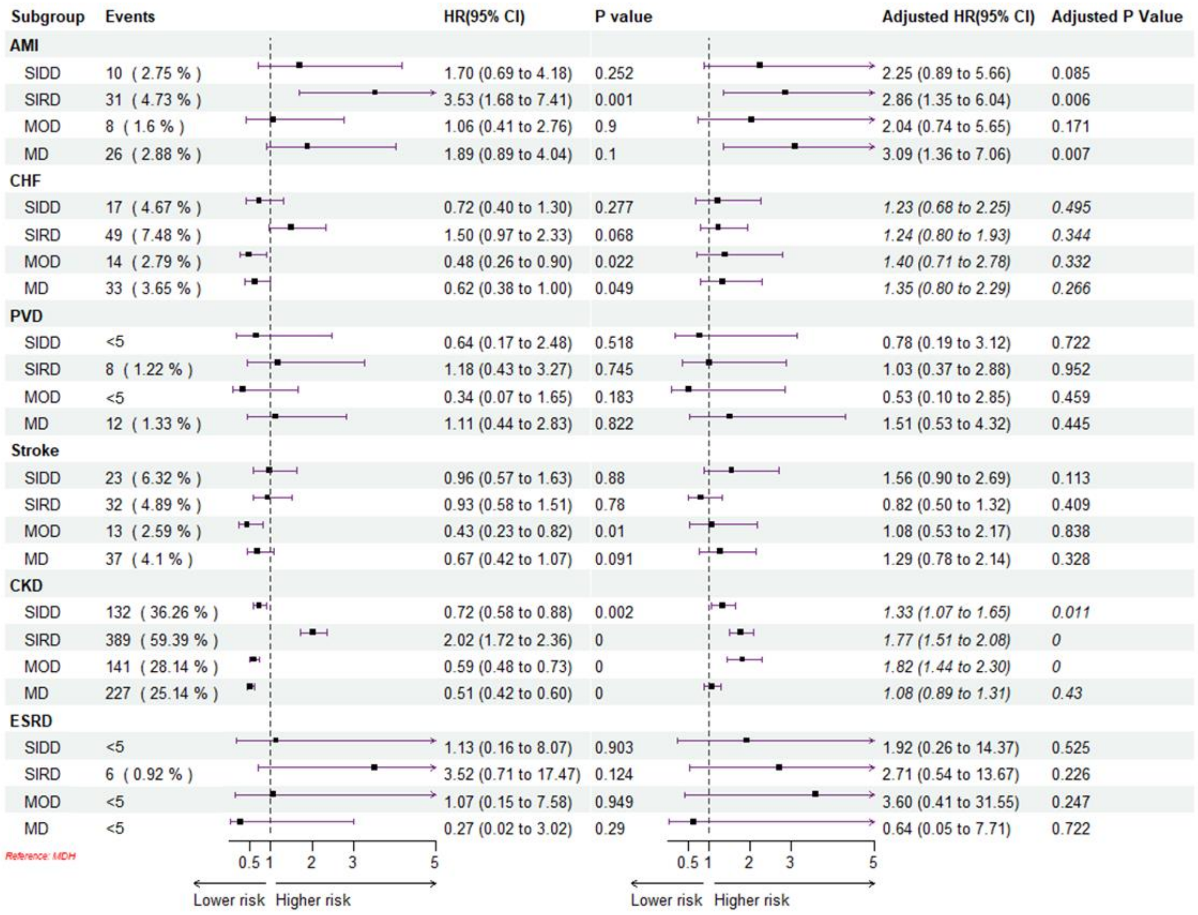

Here, SIDD, SIRD, MOD, MD and MDH refer to RHAP-SIDD, RHAP-SIRD, RHAP-MOD, RHAP-MD and RHAP-MDH, respectively.
MDH is the reference group. The adjusted HR and P value reflect the results after adjustment for age and sex. Italics indicate that the proportional hazard assumption has not been fulfilled.
DCS, Hoorn Diabetes Care System (Netherlands); SIDD, Severe Insulin Deficiency Diabetes; SIRD, Severe Insulin Resistance Diabetes; MOD, Mild Obesity-related Diabetes; MD, Mild Diabetes; MDH, Mild Diabetes with high HDL-cholesterol; AMI, Acute Myocardial Infarction; CHF, Congestive Heart Failure; PVD, Peripheral Vascular Diseases; CKD, Chronic Kidney Disease; ESRD, End Stage Renal Disease

ESM Appendix 6 Treatment patterns

ESM Table 6.1 The results of multinomial logistic regressions (relative risk ratios) for comparing treatment steps

DCS					
	**Only CVD**	**Step1**	**Step2**	**Step3**	**Other OAD**
**RHAP-SIDD**	0.40 (0.28-0.59) ***	3.57 (2.72-4.68) ***	8.18 (6.25-10.71) ***	11.80 (8.98-15.50) ***	11.56 (7.91-16.89) ***
**RHAP-SIRD**	2.67 (2.27-3.14) ***	1.99 (1.71-2.32) ***	2.10 (1.80-2.46) ***	0.92 (0.77-1.10)	2.44 (1.78-3.35) ***
**RHAP-MOD**	1.06 (0.90-1.25)	1.44 (1.24-1.68) ***	1.45 (1.24-1.69) ***	1.22 (1.03-1.43) *	4.84 (3.71-6.33) ***
**RHAP-MDH**	1.22 (1.07-1.39) **	0.89 (0.79-1.01) .	0.76 (0.67-0.86) ***	0.42 (0.37-0.49) ***	0.73 (0.53-1.01) .
**GoDARTS**					
	**Only CVD**	**Step1**	**Step2**	**Step3**	**Other OAD**
**RHAP-SIDD**	0.29 (0.26-0.33) ***	1.17 (1.05-1.3) **	2.04 (1.83-2.27) ***	3.07 (2.73-3.44) ***	2.59 (2.32-2.90) ***
**RHAP-SIRD**	1.89 (1.68-2.13) ***	1.70 (1.50-1.93) ***	2.08 (1.84-2.36) ***	1.23 (1.07-1.42) **	1.67 (1.46-1.91) ***
**RHAP-MOD**	0.64 (0.58-0.71) ***	1.35 (1.22-1.5) ***	0.93 (0.84-1.04)	1.29 (1.15-1.45) ***	2.58 (2.31-2.88) ***
**RHAP-MDH**	1.45 (1.31-1.61) ***	1.01 (0.90-1.13)	1.08 (0.96-1.21)	0.65 (0.57-0.74) ***	0.74 (0.65-0.84) ***

DCS, Hoorn Diabetes Care System (Netherlands); GoDARTS, Genetics of Diabetes Audit and Research in Tayside Scotland (Scotland); SIDD, Severe Insulin Deficiency Diabetes; SIRD, Severe Insulin Resistance Diabetes; MOD, Mild Obesity-related Diabetes; MD, Mild Diabetes; MDH, Mild Diabetes with high HDL-cholesterol

The treatment step was defined by an individual's first available observation in each diabetes duration interval. Treatment steps are defined as no common treatment (diet and exercise), only CVD treatment (Anatomical Therapeutic Chemical Classification System: C01–C10), step 1 (adding metformin [A10BA02] or repaglinid and nateglinide [A10BX]), step 2 (adding sulfonylurea [A10BB]), step 3 (adding insulin [A10A]) and other OAD (dipeptidyl peptidase-4 inhibitors [A10BH], glucagon-like peptide-1 [A10BJ], α-glucosidase inhibitors [A10BF], sodium–glucose cotransporter 2 inhibitors [A10BK], thiazolidinediones [A10BG], liraglutide [A10BX07], dapagliflozin [A10BX09])

Values are reported as relative risk ratios with 95% CI, and are adjusted for diabetes duration and sex.

MD was the reference group.

Significance stars are denoted as 0-0.001, "***", 0.001-0.01, "**", 0.01-0.05, "*", 0.05-0.1, "."

ESM Figure 6.1 Area graph of treatment steps per individual over time for each subgroup in the DCS cohort

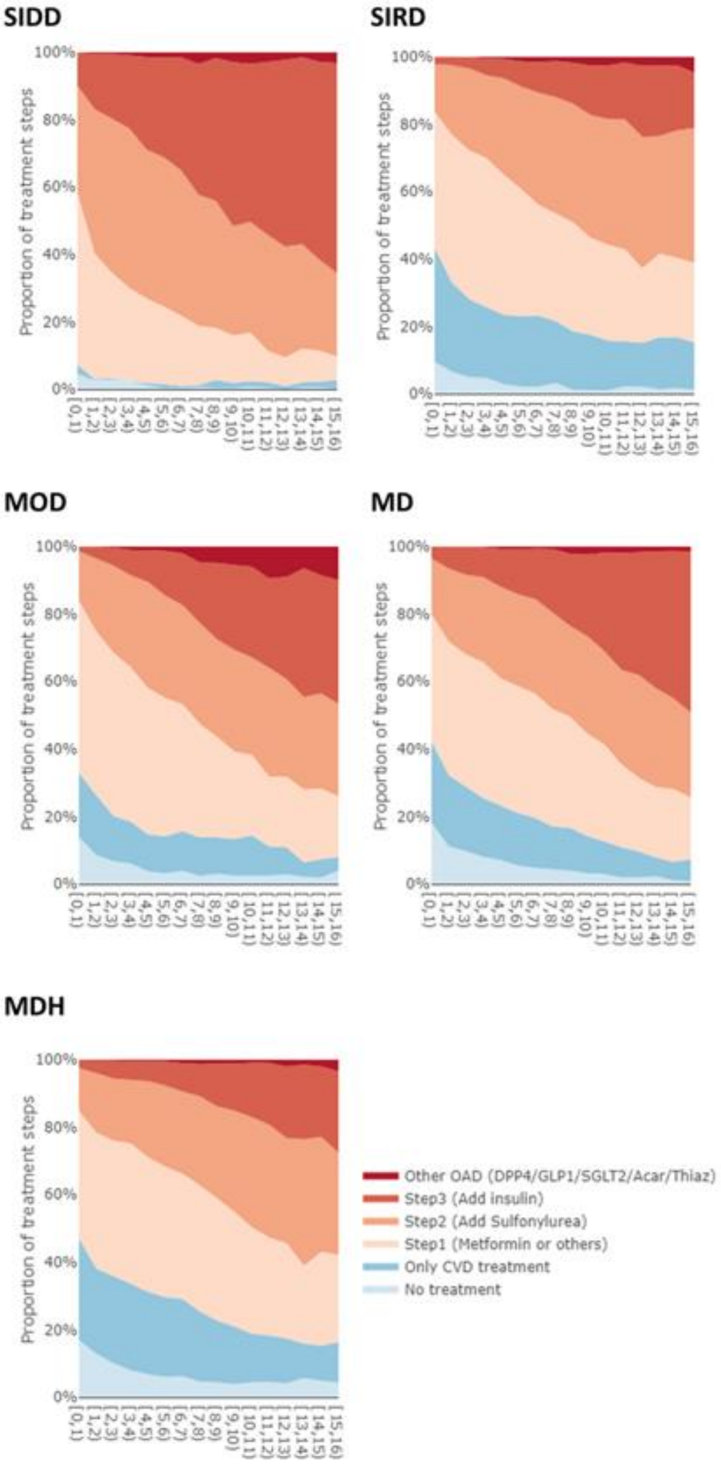

Here, SIDD, SIRD, MOD, MD and MDH refer to RHAP-SIDD, RHAP-SIRD, RHAP-MOD, RHAP-MD and RHAP-MDH, respectively.

DCS, Hoorn Diabetes Care System (Netherlands); SIDD, Severe Insulin Deficiency Diabetes; SIRD, Severe Insulin Resistance Diabetes; MOD, Mild Obesity-related Diabetes; MD, Mild Diabetes; MDH, Mild Diabetes with high HDL-cholesterol

The treatment step was defined by an individual’s first available observation in each diabetes duration interval. Treatment steps are defined as no common treatment (diet and exercise), only CVD treatment (Anatomical Therapeutic Chemical Classification System: C01–C10), step 1 (adding metformin [A10BA02] or repaglinid and nateglinide [A10BX]), step 2 (adding sulfonylurea [A10BB]), step 3 (adding insulin [A10A]) and other OAD (dipeptidyl peptidase-4 inhibitors [A10BH], glucagon-like peptide-1 [A10BJ], α-glucosidase inhibitors [A10BF], sodium–glucose cotransporter 2 inhibitors [A10BK], thiazolidinediones [A10BG], liraglutide [A10BX07], dapagliflozin [A10BX09])

ESM Appendix 7 Comparison between subgroups and clinical features to predict outcomes

ESM Figure 7.1 Comparisons between model fits with different formats of covariates to indicate subgroups

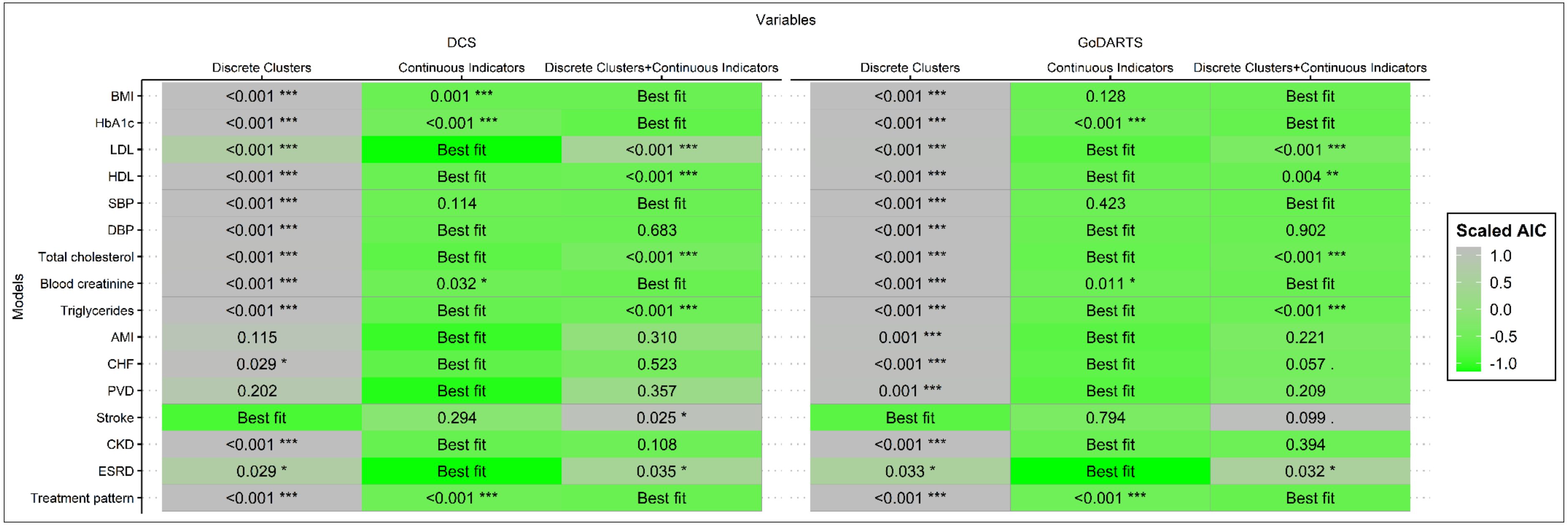

DCS, Hoorn Diabetes Care System (Netherlands); GoDARTS, Genetics of Diabetes Audit and Research in Tayside Scotland (Scotland); AMI, Acute Myocardial Infarction; CHF, Congestive Heart Failure; PVD, Peripheral Vascular Diseases; CKD, Chronic Kidney Disease; ESRD, End Stage Renal Disease; AIC, Akaike Information Criterion

Smaller (greener) AIC values indicate better goodness of fit. The P-value for the comparison of AIC differences was indicated in the text. In most cases (only except for stroke), compared to using discrete clusters, the fitting is better when clustering indicators are applied. However, in many cases (for BMI, HbA_{1c}, Systolic BP, blood creatinine, and treatment pattern), the fitting is even better when both discrete clustering indicators and discrete clusters are used in combination.

Significance stars are denoted as 0-0.001, "***", 0.001-0.01, "**", 0.01-0.05, "*", 0.05-0.1, "."

ESM Table 7.1 The result of all random intercept models of DCS for the comparison between subgroups and clinical features to predict outcomes

	BMI	HbA_{1c}	LDL-cholesterol	HDL-cholesterol	Systolic BP	Diastolic BP	Total cholesterol	Blood creatinine	Triglycerides
**Intercept**	30.518 ***	56.155 ***	3.015 ***	1.327 ***	141.349 ***	79.535 ***	5.162 ***	67.896 ***	1.799 ***
**Diabetes duration**	-0.047 ***	0.268 ***	-0.059 ***	0.003 ***	0.164 ***	-0.289 ***	-0.063 ***	0.624 ***	-0.013 ***
**Male**	-1.320 ***	0.922 ***	-0.222 ***	-0.208 ***	-2.198 ***	1.608 ***	-0.432 ***	15.847 ***	0.005
**RHAP-SIRD**	0.520 *	-9.649 ***	-0.185 ***	-0.084 ***	3.642 ***	-1.651 ***	-0.203 ***	10.763 ***	0.163 ***
**RHAP-MOD**	7.917 ***	-6.324 ***	-0.079 .	-0.110 ***	-3.122 **	2.535 ***	-0.052	-0.648	0.333 ***
**RHAP-MD**	-0.779 **	-7.736 ***	0.039	-0.069 ***	-3.196 ***	0.398	0.018	-1.264	0.108 *
**RHAP-MDH**	-2.334 ***	-10.562 ***	-0.003	0.361 ***	3.354 ***	-1.370 ***	0.180 ***	2.451 *	-0.391 ***
**AIC**	151125	265857	78318	-22907	301515	246918	88352	285532	70135
**RL**	<0.001	<0.001	<0.001	<0.001	<0.001	<0.001	<0.001	<0.001	<0.001
**Intercept**	3.298 ***	38.717 ***	3.441 ***	0.228 ***	92.020 ***	82.318 ***	5.034 ***	32.410 ***	2.988 ***
**Diabetes duration**	-0.045 ***	0.263 ***	-0.059 ***	0.003 ***	0.173 ***	-0.290 ***	-0.063 ***	0.629 ***	-0.013 ***
**Male**	-0.093	-0.017	-0.224 ***	-0.067 ***	-0.646	1.652 ***	-0.351 ***	17.195 ***	-0.121 ***
**HbA_{1c} at baseline**	0.065 **	2.682 ***	-0.011	0.003 .	0.518 **	-0.059	-0.006	0.048	0.001
**HDL-cholesterol at baseline**	0.466 ***	-1.714 ***	0.096 *	0.827 ***	1.101	0.605	0.594 ***	-0.876	-0.719 ***
**C peptide at baseline**	0.116	-1.143 ***	-0.193 ***	-0.067 ***	-0.397	0.100	-0.095 **	7.741 ***	0.400 ***
**Age at baseline**	-0.021 ***	-0.100 ***	-0.006 ***	0.001 .	0.615 ***	-0.154 ***	-0.010 ***	0.523 ***	-0.010 ***
**BMI at baseline**	0.898 ***	0.017	0.001	0.002 ***	0.236 ***	0.192 ***	0.003	-0.061	-0.001
**AIC**	146350	265193	**78297**	**-25576**	301143	**246786**	**88241**	285263	**69745**
**RL**	0.001	<0.001	**Reference**	**Reference**	0.114	**Reference**	**Reference**	0.032	**Reference**
**Intercept**	4.815 ***	28.680 ***	3.444 ***	0.249 ***	91.405 ***	82.648 ***	4.963 ***	33.040 ***	2.798 ***
**Diabetes duration**	-0.045 ***	0.264 ***	-0.059 ***	0.003 ***	0.173 ***	-0.290 ***	-0.063 ***	0.629 ***	-0.013 ***
**Male**	-0.052	-0.055	-0.212 ***	-0.070 ***	-0.623	1.742 ***	-0.340 ***	17.276 ***	-0.118 ***
**RHAP-SIRD**	-0.799 ***	5.749 ***	-0.119 .	0.016	-1.413	-1.370 *	-0.098	0.916	-0.007
**RHAP-MOD**	-0.472 *	6.004 ***	-0.117	-0.002	0.337	-0.878	-0.110	-1.215	0.026
**RHAP-MD**	-0.783 ***	5.333 ***	-0.051	-0.003	0.177	-0.517	-0.017	-0.549	0.073

**RHAP-MDH**	-1.003 ***	4.164 ***	-0.120 .	0.015	0.536	-0.833	-0.069	-1.656	0.069
**HbA_{1c} at baseline**	-0.073 *	3.554 ***	-0.025 .	0.004	0.517 .	-0.187	-0.015	-0.052	0.011
**HDL-cholesterol at baseline**	0.624 ***	-0.762	0.129 *	0.818 ***	0.405	0.613	0.611 ***	0.346	-0.738 ***
**C peptide at baseline**	0.099	-1.692 ***	-0.172 ***	-0.071 ***	0.172	0.394	-0.066 .	7.169 ***	0.427 ***
**Age at baseline**	-0.016 ***	-0.094 ***	-0.005 **	0.000	0.639 ***	-0.142 ***	-0.009 ***	0.501 ***	-0.010 ***
**BMI at baseline**	0.888 ***	-0.040	0.003	0.002 **	0.219 **	0.200 ***	0.005	-0.027	0.000
**AIC**	**146337**	**265107**	78315	-25544	**301139**	246787	88260	**285256**	69765
**RL**	**Reference**	**Reference**	<0.001	<0.001	**Reference**	0.683	<0.001	**Reference**	<0.001

DCS, Hoorn Diabetes Care System (Netherlands); SIDD, Severe Insulin Deficiency Diabetes; SIRD, Severe Insulin Resistance Diabetes; MOD, Mild Obesity-related Diabetes; MD, Mild Diabetes; MDH, Mild Diabetes with high HDL-cholesterol; AIC, Akaike Information Criterion; RL, Relative Likelihood
Significance stars are denoted as 0-0.001, "***", 0.001-0.01, "**", 0.01-0.05, "*", 0.05-0.1, "."

ESM Table 7.2 The result of all Cox models of DCS for the comparison between subgroups and clinical features to predict outcomes

	**AMI**	**CHF**	**PVD**	**Stroke**	**CKD**	**ESRD**
	Hazard ratio (95% CI)	Hazard ratio (95% CI)	Hazard ratio (95% CI)	Hazard ratio (95% CI)	Hazard ratio (95% CI)	Hazard ratio (95% CI)
**RHAP-SIDD**	2.25 (0.89 - 5.66) .	1.23 (0.68 - 2.25)	0.78 (0.19 - 3.12)	1.56 (0.9 - 2.69)	1.33 (1.07 - 1.65) *	1.92 (0.26 - 14.37)
**RHAP-SIRD**	2.86 (1.35 - 6.04) **	1.24 (0.8 - 1.93)	1.03 (0.37 - 2.88)	0.82 (0.5 - 1.32)	1.77 (1.51 - 2.08) ***	2.71 (0.54 - 13.67)
**RHAP-MOD**	2.04 (0.74 - 5.65)	1.4 (0.71 - 2.78)	0.53 (0.1 - 2.85)	1.08 (0.53 - 2.17)	1.82 (1.44 - 2.3) ***	3.6 (0.41 - 31.55)
**RHAP-MD**	3.09 (1.36 - 7.06) **	1.35 (0.8 - 2.29)	1.51 (0.53 - 4.32)	1.29 (0.78 - 2.14)	1.08 (0.89 - 1.31)	0.64 (0.05 - 7.71)
**Male**	3.36 (1.95 - 5.79) ***	1.53 (1.08 - 2.16) *	2.14 (0.97 - 4.72) .	1 (0.71 - 1.41)	0.8 (0.71 - 0.91) ***	1.35 (0.43 - 4.24)
**Age at baseline**	1.05 (1.02 - 1.08) **	1.08 (1.06 - 1.11) ***	1.03 (0.98 - 1.08)	1.07 (1.05 - 1.09) ***	1.09 (1.08 - 1.1) ***	1.11 (1.02 - 1.19) *
**AIC**	1276	2205	501	2122	16362	185
**RL**	0.115	0.029	0.202	**Reference**	0	0.029
**HDL-cholesterol at baseline**	0.27 (0.11 - 0.66) **	0.97 (0.54 - 1.72)	0.89 (0.26 - 3.04)	0.92 (0.52 - 1.64)	1.01 (0.82 - 1.23)	1.19 (0.14 - 9.77)
**HbA_{1c} at baseline**	1.02 (0.89 - 1.17)	1.1 (1 - 1.22) .	1.05 (0.85 - 1.3)	1.09 (0.98 - 1.2)	1.04 (1 - 1.08) .	1.04 (0.73 - 1.47)
**BMI at baseline**	0.99 (0.94 - 1.04)	1.02 (0.99 - 1.06)	0.92 (0.84 - 1) .	1 (0.96 - 1.04)	1.01 (1 - 1.02)	1.12 (1.02 - 1.22) *
**C peptide at baseline**	1.5 (0.96 - 2.32) .	1.26 (0.89 - 1.77)	2.13 (1.07 - 4.24) *	0.9 (0.61 - 1.32)	2.05 (1.81 - 2.32) ***	2.57 (1.01 - 6.57) *
**Age at baseline**	1.05 (1.02 - 1.07) ***	1.08 (1.06 - 1.1) ***	1.02 (0.98 - 1.06)	1.06 (1.04 - 1.08) ***	1.09 (1.09 - 1.1) ***	1.13 (1.05 - 1.21) ***
**Male**	2.73 (1.56 - 4.77) ***	1.57 (1.09 - 2.25) *	1.89 (0.84 - 4.23)	0.92 (0.65 - 1.32)	0.89 (0.79 - 1.01) .	2.23 (0.64 - 7.79)
**AIC**	**1272**	**2198**	**498**	2125	**16282**	**178**
**RL**	**Reference**	**Reference**	**Reference**	0.294	**Reference**	**Reference**
**RHAP-SIDD**	0.89 (0.23 - 3.48)	0.4 (0.15 - 1.04) .	0.22 (0.03 - 1.87)	1.21 (0.47 - 3.13)	1.16 (0.82 - 1.65)	1.74 (0.06 - 46.97)
**RHAP-SIRD**	1.21 (0.47 - 3.09)	0.89 (0.48 - 1.64)	0.5 (0.12 - 2.11)	0.73 (0.38 - 1.4)	1.21 (0.97 - 1.51) .	1 (0.12 - 7.97)
**RHAP-MOD**	0.61 (0.15 - 2.4)	0.62 (0.24 - 1.59)	0.28 (0.03 - 2.68)	0.88 (0.34 - 2.26)	1.3 (0.94 - 1.8)	0.38 (0.02 - 7.81)
**RHAP-MD**	1.75 (0.67 - 4.54)	1.2 (0.64 - 2.25)	1.32 (0.35 - 4.96)	1.2 (0.65 - 2.23)	1.11 (0.88 - 1.4)	0.52 (0.03 - 8.33)
**HDL-cholesterol at baseline**	0.34 (0.12 - 1.01) .	0.99 (0.48 - 2.02)	0.82 (0.18 - 3.78)	0.91 (0.44 - 1.87)	1.12 (0.88 - 1.44)	0.96 (0.08 - 11.9)
**HbA_{1c} at baseline**	1.1 (0.88 - 1.39)	1.28 (1.08 - 1.52) **	1.33 (0.93 - 1.9)	1.05 (0.88 - 1.26)	1.03 (0.97 - 1.1)	0.91 (0.5 - 1.64)
**BMI at baseline**	1.02 (0.96 - 1.08)	1.04 (0.99 - 1.08) .	0.95 (0.85 - 1.05)	1.01 (0.96 - 1.05)	1 (0.99 - 1.02)	1.15 (1.02 - 1.29) *
**C peptide at baseline**	1.67 (1.03 - 2.72) *	1.34 (0.91 - 1.96)	2.88 (1.31 - 6.3) **	1.06 (0.69 - 1.64)	1.95 (1.7 - 2.25) ***	2.52 (0.87 - 7.32) .
**Age at baseline**	1.05 (1.02 - 1.08) **	1.08 (1.06 - 1.11) ***	1.03 (0.98 - 1.08)	1.07 (1.05 - 1.09) ***	1.09 (1.09 - 1.1) ***	1.11 (1.03 - 1.2) **
**Male**	3.08 (1.74 - 5.47) ***	1.63 (1.12 - 2.38) **	2.08 (0.9 - 4.79) .	0.99 (0.69 - 1.44)	0.89 (0.78 - 1.01) .	2.35 (0.65 - 8.53)
**AIC**	1274	2199	500	2130	16286	184
**RL**	0.310	0.523	0.357	0.025	0.108	0.035

DCS, Hoorn Diabetes Care System (Netherlands); SIDD, Severe Insulin Deficiency Diabetes; SIRD, Severe Insulin Resistance Diabetes; MOD, Mild Obesity-related Diabetes; MD, Mild Diabetes; MDH, Mild Diabetes with high HDL-cholesterol; AMI, Acute Myocardial Infarction; CHF, Congestive Heart Failure; PVD, Peripheral Vascular Diseases; CKD, Chronic Kidney Disease; ESRD, End Stage Renal Disease; AIC, Akaike Information Criterion; RL, Relative Likelihood

Significance stars are denoted as 0-0.001, "***", 0.001-0.01, "**", 0.01-0.05, "*", 0.05-0.1, "."

ESM Table 7.3 The results of all multinomial models of DCS for the comparison between subgroups and clinical features to predict outcomes

	Only CVD	Step1	Step2	Step3	Other OAD
**(Intercept)**	0.680 *	10.889 ***	9.994 ***	3.839 ***	0.159 ***
**RHAP-SIRD**	6.622 ***	0.559 ***	0.257 ***	0.078 ***	0.211 ***
**RHAP-MOD**	2.623 ***	0.405 ***	0.177 ***	0.103 ***	0.419 ***
**RHAP-MD**	2.478 ***	0.280 ***	0.122 ***	0.085 ***	0.086 ***
**RHAP-MDH**	3.016 ***	0.249 ***	0.093 ***	0.036 ***	0.063 ***
**Diabetes duration**	1.095 ***	1.123 ***	1.223 ***	1.383 ***	1.395 ***
**Male**	0.780 ***	0.961	1.171 **	1.136 *	1.344 **
**AIC**	102697				
**RL**	<0.001				
**(Intercept)**	0.140 ***	0.001 ***	0.001 ***	0.001 ***	0.000 ***
**HDL-cholesterol at baseline**	0.804 *	0.566 ***	0.379 ***	0.159 ***	0.381 ***
**HbA_{1c} at baseline**	0.685 ***	1.968 ***	2.460 ***	2.845 ***	2.569 ***
**BMI at baseline**	1.035 ***	1.034 ***	0.992	1.028 ***	1.102 ***
**C peptide at baseline**	1.839 ***	1.543 ***	1.612 ***	0.335 ***	1.485 ***
**Age at baseline**	1.063 ***	1.048 ***	1.046 ***	1.024 ***	1.022 ***
**Diabetes duration**	1.096 ***	1.143 ***	1.250 ***	1.416 ***	1.425 ***
**Male**	0.900 .	0.950	0.988	0.765 ***	1.208 .
**AIC**	96087				
**RL**	<0.001				
**(Intercept)**	0.050 ***	0.000 ***	0.000 ***	0.000 ***	0.000 ***
**RHAP-SIRD**	0.827	3.580 ***	3.699 ***	6.995 ***	4.924 ***
**RHAP-MOD**	0.764	3.618 ***	4.933 ***	6.234 ***	5.169 ***
**RHAP-MD**	1.045	4.618 ***	4.036 ***	5.549 ***	3.396 ***
**RHAP-MDH**	0.778	4.035 ***	3.113 ***	4.032 ***	4.122 ***
**HDL-cholesterol at baseline**	0.969	0.596 ***	0.456 ***	0.203 ***	0.391 ***
**HbA_{1c} at baseline**	0.738 ***	2.313 ***	2.844 ***	3.517 ***	2.992 ***
**BMI at baseline**	1.041 ***	1.037 ***	0.978 **	1.015 .	1.085 ***
**C peptide at baseline**	1.950 ***	1.673 ***	1.569 ***	0.297 ***	1.320 *
**Age at baseline**	1.067 ***	1.051 ***	1.051 ***	1.025 ***	1.021 **
**Diabetes duration**	1.095 ***	1.145 ***	1.253 ***	1.421 ***	1.430 ***
**Male**	0.936	0.962	1.006	0.762 ***	1.208 .
**AIC**	**95817**				
**RL**	**Reference**				

DCS, Hoorn Diabetes Care System (Netherlands); SIDD, Severe Insulin Deficiency Diabetes; SIRD, Severe Insulin Resistance Diabetes; MOD, Mild Obesity-related Diabetes; MD, Mild Diabetes; MDH, Mild Diabetes with high HDL-cholesterol; AIC, Akaike Information Criterion; RL, Relative Likelihood

The treatment step was defined by an individual's first available observation in each diabetes duration interval. Treatment steps are defined as no common treatment (diet and exercise), only CVD treatment (Anatomical Therapeutic Chemical Classification System: C01–C10), step 1 (adding metformin [A10BA02] or repaglinid and nateglinide [A10BX]), step 2 (adding sulfonylurea [A10BB]), step 3 (adding insulin [A10A]) and other OAD (dipeptidyl peptidase-4 inhibitors [A10BH], glucagon-like peptide-1 [A10BJ], α-glucosidase inhibitors [A10BF], sodium–glucose cotransporter 2 inhibitors [A10BK], thiazolidinediones [A10BG], liraglutide [A10BX07], dapagliflozin [A10BX09])

Significance stars are denoted as 0-0.001, "***", 0.001-0.01, "**", 0.01-0.05, "*", 0.05-0.1, "."

ESM Table 7.4 The result of all random intercept models of GoDARTS for the comparison between subgroups and clinical features to predict outcomes

	BMI	HbA_{1c}	LDL-cholesterol	HDL-cholesterol	Systolic BP	Diastolic BP	Total cholesterol	Blood creatinine	Triglycerides
**Intercept**	31.770 ***	62.811 ***	2.660 ***	1.313 ***	142.607 ***	81.254 ***	5.090 ***	63.684 ***	2.441 ***
**Diabetes duration**	-0.066 ***	0.304 ***	-0.058 ***	-0.005 ***	-0.439 ***	-0.702 ***	-0.086 ***	1.486 ***	-0.043 ***
**Male**	-1.936 ***	-0.686 **	-0.253 ***	-0.133 ***	-1.831 ***	0.025	-0.412 ***	15.803 ***	-0.070 **
**RHAP-SIRD**	0.637 **	-9.846 ***	-0.154 ***	-0.082 ***	-0.484	-3.439 ***	-0.182 ***	14.535 ***	0.168 ***
**RHAP-MOD**	8.598 ***	-2.550 ***	0.088 **	-0.087 ***	-0.811 .	3.019 ***	0.124 ***	-6.050 ***	0.280 ***
**RHAP-MD**	-0.657 ***	-8.934 ***	0.087 ***	-0.036 ***	-1.450 ***	-0.682 **	0.025	-0.643	0.003
**RHAP-MDH**	-2.716 ***	-11.878 ***	-0.065 *	0.372 ***	-0.050	-3.166 ***	0.032	3.897 ***	-0.576 ***
**AIC**	792801	1365932	126372	-58752	1847821	1594838	352104	2819958	177847
**RL**	<0.001	<0.001	<0.001	<0.001	<0.001	<0.001	<0.001	<0.001	<0.001
**Intercept**	5.809 ***	53.158 ***	3.777 ***	0.659 ***	127.793 ***	92.285 ***	6.327 ***	0.242	4.400 ***
**Diabetes duration**	-0.065 ***	0.300 ***	-0.058 ***	-0.005 ***	-0.436 ***	-0.704 ***	-0.086 ***	1.489 ***	-0.044 ***
**Male**	0.215 ***	-0.397 .	-0.259 ***	-0.079 ***	-1.611 ***	0.616 ***	-0.392 ***	15.124 ***	-0.148 ***
**HbA_{1c} at baseline**	0.158 ***	2.304 ***	-0.020 ***	-0.002 *	0.274 ***	0.019	-0.015 ***	0.669 ***	0.003
**HDL-cholesterol at baseline**	0.071	-2.368 ***	-0.084 **	0.695 ***	-0.088	0.672 **	0.281 ***	-3.620 ***	-0.849 ***
**C peptide at baseline**	0.139 ***	-0.359 **	-0.076 ***	-0.030 ***	-0.205	-0.480 ***	-0.030 **	4.536 ***	0.164 ***
**Age at baseline**	-0.046 ***	-0.247 ***	-0.010 ***	0.000	0.139 ***	-0.262 ***	-0.019 ***	0.839 ***	-0.019 ***
**BMI at baseline**	0.849 ***	0.098 ***	-0.003 *	-0.003 ***	0.119 ***	0.125 ***	-0.007 ***	0.121 *	-0.003 .
**AIC**	783939	1364740	**126233**	**-62672**	1847748	**1593908**	**351723**	2819206	**177166**
**RL**	0.128	<0.001	**Reference**	**Reference**	0.423	**Reference**	**Reference**	0.011	**Reference**
**Intercept**	6.654 ***	49.036 ***	3.753 ***	0.707 ***	129.963 ***	93.056 ***	6.306 ***	-4.166	4.329 ***
**Diabetes duration**	-0.065 ***	0.301 ***	-0.058 ***	-0.005 ***	-0.436 ***	-0.704 ***	-0.086 ***	1.489 ***	-0.044 ***
**Male**	0.228 ***	-0.527 *	-0.258 ***	-0.079 ***	-1.706 ***	0.568 ***	-0.395 ***	15.276 ***	-0.153 ***
**RHAP-SIRD**	-0.468 **	5.045 ***	-0.019	-0.014	-1.351 .	-0.971 *	0.020	3.000 *	0.088
**RHAP-MOD**	-0.596 ***	5.352 ***	-0.012	-0.006	-0.221	-0.308	0.059	0.737	0.128 *

**RHAP-MD**	-0.554 ***	3.494 ***	0.003	-0.016	-1.255 *	-0.677 *	0.026	2.262 .	0.074
**RHAP-MDH**	-0.475 **	3.779 ***	-0.016	0.028 *	-0.896	-1.068 **	0.044	0.324	0.112 *
**HbA_{1c} at baseline**	0.083 ***	2.855 ***	-0.020 **	-0.003 .	0.119	-0.087	-0.011	0.931 ***	0.015 .
**HDL-cholesterol at baseline**	0.051	-2.418 ***	-0.075 *	0.669 ***	-0.251	0.913 **	0.269 ***	-2.432 *	-0.872 ***
**C peptide at baseline**	0.136 ***	-0.765 ***	-0.072 ***	-0.028 ***	-0.114	-0.408 ***	-0.028 *	4.178 ***	0.162 ***
**Age at baseline**	-0.047 ***	-0.246 ***	-0.010 ***	0.000	0.149 ***	-0.252 ***	-0.019 ***	0.830 ***	-0.019 ***
**BMI at baseline**	0.857 ***	0.006	-0.003	-0.003 ***	0.097 ***	0.116 ***	-0.009 ***	0.144 *	-0.006 *
**AIC**	**783934**	**1364641**	126261	-62661	**1847746**	1593909	351748	**2819197**	177185
**RL**	**Reference**	**Reference**	<0.001	0.004	**Reference**	0.902	<0.001	**Reference**	<0.001

GoDARTS, Genetics of Diabetes Audit and Research in Tayside Scotland (Scotland); SIDD, Severe Insulin Deficiency Diabetes; SIRD, Severe Insulin Resistance Diabetes; MOD, Mild Obesity-related Diabetes; MD, Mild Diabetes; MDH, Mild Diabetes with high HDL-cholesterol; AIC, Akaike Information Criterion; RL, Relative Likelihood
Significance stars are denoted as 0-0.001, "***", 0.001-0.01, "**", 0.01-0.05, "*", 0.05-0.1, "."

ESM Table 7.5 The result of all Cox models of GoDARTS for the comparison between subgroups and clinical features to predict outcomes

	**AMI**	**CHF**	**PVD**	**Stroke**	**CKD**	**ESRD**
	Hazard ratio (95% CI)	Hazard ratio (95% CI)	Hazard ratio (95% CI)	Hazard ratio (95% CI)	Hazard ratio (95% CI)	Hazard ratio (95% CI)
**RHAP-SIDD**	1.2 (0.99 - 1.44) .	1.53 (1.14 - 2.06) **	1.39 (0.55 - 3.49)	1.1 (0.84 - 1.43)	1.21 (1.08 - 1.35) ***	1.66 (0.62 - 4.43)
**RHAP-SIRD**	1.72 (1.46 - 2.02) ***	1.77 (1.36 - 2.29) ***	2.11 (0.98 - 4.56) .	1.12 (0.88 - 1.42)	1.67 (1.51 - 1.84) ***	2.36 (1.23 - 4.53) *
**RHAP-MOD**	1.38 (1.12 - 1.71) **	1.9 (1.33 - 2.73) ***	1.61 (0.53 - 4.91)	1.28 (0.92 - 1.78)	1.26 (1.1 - 1.45) ***	5.2 (1.03 - 26.23) *
**RHAP-MD**	1.22 (1.03 - 1.44) *	1.34 (1.02 - 1.77) *	1.44 (0.64 - 3.24)	1.08 (0.85 - 1.38)	1.05 (0.95 - 1.17)	1.45 (0.37 - 5.73)
**Male**	1.51 (1.36 - 1.68) ***	1.28 (1.08 - 1.52) **	2.14 (1.24 - 3.7) **	1.12 (0.95 - 1.31)	0.84 (0.78 - 0.89) ***	0 (0 - Inf)
**Age at baseline**	1.03 (1.03 - 1.04) ***	1.07 (1.06 - 1.09) ***	1.07 (1.04 - 1.11) ***	1.06 (1.05 - 1.07) ***	1.08 (1.07 - 1.08) ***	1.27 (1.21 - 1.34) ***
**AIC**	24292	8815	1028	**10136**	56541	554
**RL**	0.001	<0.001	0.001	**Reference**	<0.001	0.033
**HDL-cholesterol at baseline**	0.65 (0.54 - 0.77) ***	0.63 (0.47 - 0.85) **	0.4 (0.16 - 1) .	0.96 (0.74 - 1.23)	0.87 (0.78 - 0.97) *	0.75 (0.28 - 2)
**HbA_{1c} at baseline**	0.99 (0.97 - 1.02)	1.04 (1 - 1.09) *	0.89 (0.78 - 1.03)	1.01 (0.97 - 1.05)	1.04 (1.02 - 1.06) ***	1.07 (0.92 - 1.24)
**BMI at baseline**	1.01 (1 - 1.02)	1.04 (1.03 - 1.06) ***	0.96 (0.9 - 1.01)	1 (0.98 - 1.01)	1.01 (1 - 1.02) **	1.03 (0.98 - 1.09)
**C peptide at baseline**	1.13 (1.07 - 1.19) ***	1.11 (1.02 - 1.2) *	1.4 (1.13 - 1.74) **	1.05 (0.97 - 1.14)	1.25 (1.21 - 1.29) ***	1.52 (1.2 - 1.92) ***
**Age at baseline**	1.03 (1.03 - 1.04) ***	1.08 (1.07 - 1.09) ***	1.06 (1.03 - 1.09) ***	1.06 (1.05 - 1.07) ***	1.08 (1.08 - 1.09) ***	1.26 (1.2 - 1.32) ***
**Male**	1.45 (1.3 - 1.62) ***	1.33 (1.11 - 1.59) **	1.82 (1.05 - 3.15) *	1.11 (0.95 - 1.31)	0.82 (0.77 - 0.88) ***	0 (0 - Inf)
**AIC**	**24277**	**8779**	**1014**	10137	**56422**	**547**
**RL**	**Reference**	**Reference**	**Reference**	0.794	**Reference**	**Reference**
**RHAP-SIDD**	0.98 (0.75 - 1.28)	0.97 (0.63 - 1.48)	2.65 (0.66 - 10.56)	1.08 (0.73 - 1.59)	0.85 (0.72 - 1.01) .	0.95 (0.24 - 3.85)
**RHAP-SIRD**	1.21 (0.96 - 1.52)	1.04 (0.72 - 1.5)	0.68 (0.22 - 2.09)	1.07 (0.76 - 1.51)	1.04 (0.9 - 1.2)	0.89 (0.31 - 2.52)
**RHAP-MOD**	1 (0.76 - 1.33)	0.78 (0.49 - 1.24)	1.8 (0.43 - 7.56)	1.49 (0.97 - 2.28) .	0.92 (0.77 - 1.09)	2.43 (0.32 - 18.39)
**RHAP-MD**	0.98 (0.8 - 1.2)	1 (0.72 - 1.39)	0.99 (0.37 - 2.66)	1.13 (0.84 - 1.52)	0.98 (0.87 - 1.11)	1.14 (0.27 - 4.87)
**HDL-cholesterol at baseline**	0.66 (0.53 - 0.82) ***	0.63 (0.44 - 0.9) *	0.39 (0.13 - 1.16) .	1.03 (0.75 - 1.41)	0.87 (0.76 - 0.99) *	0.72 (0.23 - 2.23)
**HbA_{1c} at baseline**	1 (0.96 - 1.04)	1.05 (0.98 - 1.12)	0.77 (0.62 - 0.96) *	1.01 (0.95 - 1.07)	1.06 (1.04 - 1.09) ***	1.07 (0.87 - 1.32)
**BMI at baseline**	1.01 (0.99 - 1.02)	1.05 (1.03 - 1.07) ***	0.95 (0.89 - 1.01) .	0.99 (0.97 - 1)	1.01 (1 - 1.02) **	1.02 (0.96 - 1.09)
**C peptide at baseline**	1.07 (1 - 1.15) *	1.09 (0.97 - 1.22)	1.61 (1.18 - 2.2) **	1.06 (0.95 - 1.18)	1.23 (1.18 - 1.29) ***	1.59 (1.14 - 2.2) **
**Age at baseline**	1.03 (1.03 - 1.04) ***	1.08 (1.07 - 1.09) ***	1.07 (1.04 - 1.1) ***	1.06 (1.05 - 1.07) ***	1.08 (1.07 - 1.08) ***	1.26 (1.2 - 1.33) ***
**Male**	1.47 (1.32 - 1.64) ***	1.35 (1.13 - 1.62) ***	1.73 (0.99 - 3.03) .	1.09 (0.92 - 1.28)	0.83 (0.77 - 0.88) ***	0 (0 - Inf)
**AIC**	24280	8784	1017	10141	56424	554
**RL**	0.221	0.057	0.209	0.099	0.394	0.032

GoDARTS, Genetics of Diabetes Audit and Research in Tayside Scotland (Scotland); SIDD, Severe Insulin Deficiency Diabetes; SIRD, Severe Insulin Resistance Diabetes; MOD, Mild Obesity-related Diabetes; MD, Mild Diabetes; MDH, Mild Diabetes with high HDL-cholesterol; AMI, Acute Myocardial Infarction; CHF, Congestive Heart Failure; PVD, Peripheral Vascular Diseases; CKD, Chronic Kidney Disease; ESRD, End Stage Renal Disease; AIC, Akaike Information Criterion; RL, Relative Likelihood
Significance stars are denoted as 0-0.001, "***", 0.001-0.01, "**", 0.01-0.05, "*", 0.05-0.1, "."

ESM Table 7.6 The results of all multinomial models of GoDARTS for the comparison between subgroups and clinical features to predict outcomes

	Only CVD	Step1	Step2	Step3	Other OAD
**(Intercept)**	1.208 ***	1.862 ***	1.907 ***	0.635 ***	0.72 ***
**RHAP-SIRD**	6.441 ***	1.456 ***	1.02	0.401 ***	0.644 ***
**RHAP-MOD**	2.185 ***	1.156 *	0.457 ***	0.421 ***	0.996
**RHAP-MD**	3.407 ***	0.855 **	0.49 ***	0.326 ***	0.386 ***
**RHAP-MDH**	4.945 ***	0.863 *	0.528 ***	0.211 ***	0.284 ***
**Diabetes duration**	1.194 ***	1.331 ***	1.392 ***	1.554 ***	1.521 ***
**Male**	0.845 ***	0.838 ***	0.902 **	0.683 ***	0.905 *
**AIC**	246177				
**RL**	<0.001				
**(Intercept)**	0.321 ***	0.01 ***	0.023 ***	0.029 ***	0.006 ***
**HDL-cholesterol at baseline**	1.317 ***	1.231 ***	0.885 .	0.709 ***	1.277 ***
**HbA_{1c} at baseline**	0.666 ***	1.099 ***	1.261 ***	1.384 ***	1.301 ***
**BMI at baseline**	1.04 ***	1.069 ***	0.994 .	1.012 **	1.063 ***
**C peptide at baseline**	1.155 ***	1.19 ***	1.317 ***	0.903 ***	1.268 ***
**Age at baseline**	1.061 ***	1.028 ***	1.03 ***	0.995 *	0.987 ***
**Diabetes duration**	1.191 ***	1.341 ***	1.405 ***	1.564 ***	1.537 ***
**Male**	0.889 **	1.01	0.903 *	0.699 ***	1.136 **
**AIC**	233496				
**RL**	<0.001				
**(Intercept)**	0.701	0.004 ***	0.009 ***	0.016 ***	0.003 ***
**RHAP-SIRD**	0.442 ***	1.184	1.533 ***	2.351 ***	1.279 *
**RHAP-MOD**	0.418 ***	1.047	1.714 ***	1.228 *	1.443 ***
**RHAP-MD**	0.497 ***	1.421 ***	1.64 ***	1.315 **	1.295 **
**RHAP-MDH**	0.381 ***	0.99	1.116	0.918	0.745 **
**HDL-cholesterol at baseline**	1.524 ***	1.472 ***	1.088	0.899	1.752 ***
**HbA_{1c} at baseline**	0.611 ***	1.138 ***	1.348 ***	1.447 ***	1.343 ***
**BMI at baseline**	1.047 ***	1.075 ***	0.985 ***	1.011 *	1.053 ***
**C peptide at baseline**	1.182 ***	1.209 ***	1.301 ***	0.798 ***	1.243 ***
**Age at baseline**	1.063 ***	1.028 ***	1.033 ***	0.994 *	0.991 ***
**Diabetes duration**	1.189 ***	1.342 ***	1.407 ***	1.566 ***	1.539 ***
**Male**	0.903 *	1.044	0.904 *	0.72 ***	1.126 **
**AIC**	**232667**				
**RL**	**Reference**				

GoDARTS, Genetics of Diabetes Audit and Research in Tayside Scotland (Scotland); SIDD, Severe Insulin Deficiency Diabetes; SIRD, Severe Insulin Resistance Diabetes; MOD, Mild Obesity-related Diabetes; MD, Mild Diabetes; MDH, Mild Diabetes with high HDL-cholesterol; AIC, Akaike Information Criterion; RL, Relative Likelihood

The treatment step was defined by an individual’s first available observation in each diabetes duration interval. Treatment steps are defined as no common treatment (diet and exercise), only CVD treatment (Anatomical Therapeutic Chemical Classification System: C01–C10), step 1 (adding metformin [A10BA02] or repaglinid and nateglinide [A10BX]), step 2 (adding sulfonylurea [A10BB]), step 3 (adding insulin [A10A]) and other OAD (dipeptidyl peptidase-4 inhibitors [A10BH], glucagon-like peptide-1 [A10BJ], α-glucosidase inhibitors [A10BF], sodium–glucose cotransporter 2 inhibitors [A10BK], thiazolidinediones [A10BG], liraglutide [A10BX07], dapagliflozin [A10BX09])

Significance stars are denoted as 0-0.001, "***", 0.001-0.01, "**", 0.01-0.05, "*", 0.05-0.1, "."

ESM Appendix 8 Example of the added value of subgroups on top of clustering indicators

Here we compare three options by which to characterize an individual: (1) subgroup, (2) clustering indicators, and (3) both clustering indicators and subgroup.

Among these three options, our study showed that using (2) clustering indicators can perform equally well or better than using (1) subgroups in most cases (ESM Appendix 7). This is consistent with previous findings [17], but we also show that using (3) both clustering indicators and subgroups may lead to the best performance. This indicated that data-driven subgroups, reflecting different phenotypes, still offer additional benefit on top of directly using the actual risk factor levels.

For example, consider two individuals A and B at diagnosis,

A) a female with HDL-cholesterol 1 mmol/l, HbA_{1c} 8.8% (73 mmol/mol), BMI 43.8 kg/m^2, C peptide 1.1 nmol/L, and age 36.1 years,

B) a male with HDL-cholesterol 0.8 mmol/l, HbA_{1c} 7.2% (55 mmol/mol), BMI 34.7 kg/m^2, C peptide 1.9 nmol/L, and age 42.1 years.

Both A and B would be allocated into the RHAP-MOD subgroup based on their characteristics.

We can generate predictions for their clinical indicators using various information options based on our models.

(1) Subgroup

A female with RHAP-MOD would be predicted to have HbA_{1c} of 50.1 mmol/mol (95% CI 32.7 mmol/mol - 66.8 mmol/mol) or 6.7% (5.1% - 8.3%), BMI of 33.1 kg/m^2 (30.0 kg/m^2 - 36.2 kg/m^2), and Systolic BP of 109 mmHg (81.9 mmHg -137 mmHg) in 5 years.

A male with RHAP-MOD would be predicted to have HbA_{1c} of 51.3 mmol/mol (95% CI 33.4 mmol/mol - 67.4 mmol/mol) or 6.8% (5.2% - 8.3%), BMI of 31.7 kg/m^2 (28.4 kg/m^2 - 35.1 kg/m^2), and Systolic BP of 107 mmHg (81.6 mmHg -133 mmHg) in 5 years.

(2) Clustering indicators

A female with HDL-cholesterol 1 mmol/l, HbA_{1c} 8.8% (73 mmol/mol), BMI 43.8 kg/m^2, C peptide 1.1 nmol/L, and aged 36.1 years would be predicted to have HbA_{1c} of 56.1 mmol/mol (95% CI 39.3 mmol/mol - 73.1 mmol/mol) or 7.3% (5.7% - 8.8%), BMI of 42.3 kg/m^2 (38.9 kg/m^2 - 45.6 kg/m^2), and Systolic BP of 109 mmHg (82.3 mmHg -135 mmHg) in 5 years.

A male with HDL-cholesterol 0.8 mmol/l, HbA_{1c} 7.2% (55 mmol/mol), BMI 34.7 kg/m^2, C peptide 1.9 nmol/L, and aged 42.1 years would be predicted to have HbA_{1c} of 49.7 mmol/mol (95% CI 33.4 mmol/mol - 67.2 mmol/mol) or 6.7% (5.2% - 8.3%), BMI of 33.8 kg/m^2 (30.5 kg/m^2 - 37.1 kg/m^2), and Systolic BP of 109 mmHg (82.4 mmHg - 137 mmHg) in 5 years.

(3) Both clustering indicators and subgroup

A female from RHAP-MOD subgroup with HDL-cholesterol 1 mmol/l, HbA_{1c} 8.8%, BMI 43.8 kg/m^2, C peptide 1.1 nmol/L, and aged 36.1 years would be predicted to have HbA_{1c} of 57.8 mmol/mol (95% CI 42.0 mmol/mol - 74.8 mmol/mol) or 7.4% (6% - 9%), BMI of 42 kg/m^2 (38.7 kg/m^2 - 45.4 kg/m^2), and Systolic BP of 110 mmHg (82.5 mmHg - 136 mmHg) in 5 years.

A male from RHAP-MOD subgroup with HDL-cholesterol 0.8 mmol/l, HbA_{1c} 7.2%, BMI 34.7 kg/m^2, C peptide 1.9 nmol/L, and aged 42.1 years would be predicted to have HbA_{1c} of 51.4 mmol/mol (95% CI 33.8 mmol/mol - 69.0 mmol/mol) or 6.9% (5.2% - 8.5%), BMI of 34 kg/m^2 (30.6 kg/m^2 – 37.1 kg/m^2), and Systolic BP of 111 mmHg (82.9 mmHg - 137 mmHg) in 5 years.

Predictions from the combined approach, (3) Both clustering indicators and subgroup, demonstrated the best performance in our current datasets.

ESM Appendix 9 Consistency of subgroups classification

ESM Table 9.1 Subgroup redistributions' overall statistics over time

		Accuracy (95% CI)	Kappa	Average Sensitivity	Average Specificity	Specific agreement on a positive rating	Proportion staying in the cluster
DCS	De novo clustering						
	2-4 years (N=3,005)	0.7 (0.69 - 0.72)	0.62	0.67	0.92	0.66	0.7
	4-6 years (N=2,920)	0.68 (0.66 - 0.7)	0.59	0.65	0.92	0.64	0.58
	6-8 years (N=2,790)	0.67 (0.65 - 0.69)	0.58	0.64	0.92	0.64	0.51
	Clustering based on centers identified at baseline						
	2-4 years (N=3,005)	0.72 (0.7 - 0.74)	0.64	0.68	0.93	0.68	0.72
	4-6 years (N=2,920)	0.71 (0.69 - 0.73)	0.63	0.67	0.93	0.67	0.61
	6-8 years (N=2,790)	0.69 (0.67 - 0.7)	0.6	0.65	0.92	0.65	0.54
GoDARTS	De novo clustering						
	2-4 years (N=6,103)	0.64 (0.63 - 0.65)	0.54	0.64	0.91	0.63	0.63
	4-6 years (N=5,851)	0.61 (0.59 - 0.62)	0.5	0.61	0.9	0.6	0.51
	6-8 years (N=5,388)	0.59 (0.58 - 0.61)	0.49	0.6	0.9	0.59	0.43
	Clustering based on centers identified at baseline						
	2-4 years (N=6,103)	0.68 (0.67 - 0.69)	0.59	0.68	0.92	0.67	0.67
	4-6 years (N=5,851)	0.65 (0.64 - 0.66)	0.56	0.65	0.91	0.64	0.54
	6-8 years (N=5,388)	0.61 (0.6 - 0.63)	0.51	0.62	0.9	0.61	0.46

DCS, Hoorn Diabetes Care System (Netherlands); GoDARTS, Genetics of Diabetes Audit and Research in Tayside Scotland (Scotland)
The interval is closed on the left (e.g. 2-4 years indicated the interval included year 2 and year 3.99…, but not year 4. Year 4 is included in the interval 4-6 years.). If multiple measurements existed within a time block, the first measurement was adopted for clustering.
N indicated the number of individuals with available clustering indicators in each period (i.e., 2-4 years, 4-6 years, or 6-8 years).
Sensitivity indicated how many individuals belong to the particular subgroup are correctly identified as the members of that subgroup, setting the subgroup identified at diagnosis as the reference subgroup.

Specificity indicated how many individuals not belong to the particular subgroup are correctly identified as not the members of that subgroup, setting the subgroup identified at diagnosis as the reference subgroup.
Specific agreement on a positive rating indicated the answer that suppose one individual belongs to a particular subgroup at diagnosis, what is the probability that he also belongs to that subgroup in another period.
Proportion staying in the cluster indicated the proportion of individuals who never move into any other cluster since the diagnosis of diabetes.

ESM Table 9.2 Scaled clustering centers

**DCS**											
		**Female**					**Male**				
**Diabetes duration**	Subgroup	HDL-cholesterol	HbA_{1c}	BMI	C-peptide	Age at diagnosis	HDL-cholesterol	HbA_{1c}	BMI	C-peptide	Age at diagnosis
**2-4 years**	RHAP-SIDD	-0.48	2.07	-0.1	-0.56	-0.54	-0.4	2.21	0.16	-0.46	-0.64
	RHAP-SIRD	-0.42	-0.2	0.24	1.37	0.66	-0.34	-0.15	-0.16	0.52	0.89
	RHAP-MOD	-0.43	0.01	1.33	0.28	-0.94	-0.48	-0.09	1.42	0.93	-0.57
	RHAP-MD	-0.31	-0.33	-0.55	-0.43	-0.11	-0.25	-0.26	-0.31	-0.5	-0.64
	RHAP-MDH	1.25	-0.27	-0.47	-0.51	0.6	1.37	-0.41	-0.68	-0.56	0.52
**4-6 years**	RHAP-SIDD	-0.59	1.76	0.1	-0.39	-0.59	-0.39	2.52	0.1	-0.54	-0.7
	RHAP-SIRD	-0.47	-0.14	0.01	1.4	0.83	-0.39	-0.23	-0.26	0.41	0.86
	RHAP-MOD	-0.3	-0.13	1.38	0.47	-0.69	-0.45	0.07	1.44	0.94	-0.46
	RHAP-MD	-0.26	-0.4	-0.53	-0.44	-0.18	-0.2	-0.11	-0.21	-0.48	-0.74
	RHAP-MDH	1.32	-0.23	-0.49	-0.53	0.62	1.39	-0.49	-0.68	-0.53	0.58
**6-8 years**	RHAP-SIDD	-0.44	1.77	0.05	-0.43	-0.54	-0.35	1.88	0.24	-0.5	-0.76
	RHAP-SIRD	-0.53	-0.12	0.09	1.35	0.67	-0.4	-0.17	-0.16	0.59	0.86
	RHAP-MOD	-0.29	-0.1	1.46	0.36	-0.77	-0.58	0.01	1.66	1.01	-0.5
	RHAP-MD	-0.29	-0.37	-0.56	-0.42	-0.07	-0.24	-0.33	-0.26	-0.4	-0.65
	RHAP-MDH	1.37	-0.33	-0.45	-0.5	0.51	1.33	-0.44	-0.63	-0.5	0.59
**GoDARTS**											
		**Female**					**Male**				
**Diabetes duration**	Subgroup	HDL-cholesterol	HbA_{1c}	BMI	C-peptide	Age at diagnosis	HDL-cholesterol	HbA_{1c}	BMI	C-peptide	Age at diagnosis
**2-4 years**	RHAP-SIDD	-0.45	2	0.16	-0.45	-0.61	-0.45	1.87	0.17	-0.33	-0.68
	RHAP-SIRD	-0.33	-0.19	-0.12	1.4	0.61	-0.3	-0.26	0.06	1.51	0.64
	RHAP-MOD	-0.38	-0.07	1.32	0.08	-0.97	-0.27	-0.18	1.06	-0.09	-0.92
	RHAP-MD	-0.33	-0.34	-0.39	-0.47	-0.07	-0.28	-0.31	-0.48	-0.4	0.32
	RHAP-MDH	1.29	-0.36	-0.62	-0.41	0.74	1.65	-0.4	-0.65	-0.42	0.44
**4-6 years**	RHAP-SIDD	-0.47	2	0.3	-0.29	-0.7	-0.46	1.82	0.11	-0.35	-0.68
	RHAP-SIRD	-0.42	-0.23	-0.14	1.41	0.59	-0.27	-0.3	0.01	1.6	0.62
	RHAP-MOD	-0.25	-0.15	1.29	-0.02	-0.89	-0.27	-0.1	1.02	-0.11	-0.84
	RHAP-MD	-0.27	-0.28	-0.44	-0.47	0	-0.25	-0.34	-0.51	-0.34	0.44
	RHAP-MDH	1.38	-0.48	-0.66	-0.4	0.77	1.76	-0.45	-0.62	-0.46	0.31
**6-8 years**	RHAP-SIDD	-0.52	2.05	0.39	-0.18	-0.83	-0.39	1.52	0.03	-0.36	-0.83

	RHAP-SIRD	-0.43	-0.25	-0.2	1.27	0.61	-0.31	-0.28	-0.04	1.61	0.66
	RHAP-MOD	-0.17	-0.15	1.48	-0.03	-0.85	-0.24	-0.15	1.27	-0.01	-0.77
	RHAP-MD	-0.3	-0.22	-0.32	-0.5	-0.23	-0.33	-0.37	-0.47	-0.32	0.45
	RHAP-MDH	1.2	-0.4	-0.67	-0.42	0.77	1.61	-0.45	-0.6	-0.52	0.31

DCS, Hoorn Diabetes Care System (Netherlands); GoDARTS, Genetics of Diabetes Audit and Research in Tayside Scotland (Scotland); SIDD, Severe Insulin Deficiency Diabetes; SIRD, Severe Insulin Resistance Diabetes; MOD, Mild Obesity-related Diabetes; MD, Mild Diabetes; MDH, Mild Diabetes with high HDL-cholesterol

ESM Table 9.3 Summary of stability performance for subgroups, derived from the confusion matrix

Diabetes Duration	De novo clustering						Clustering based on centers identified at baseline				
	**DCS**										
		RHAP-SIDD	RHAP-SIRD	RHAP-MOD	RHAP-MD	RHAP-MDH	RHAP-SIDD	RHAP-SIRD	RHAP-MOD	RHAP-MD	RHAP-MDH
**2-4 years**	Sensitivity	0.22	0.85	0.85	0.69	0.73	0.23	0.85	0.83	0.74	0.75
	Specificity	0.92	0.95	0.95	0.88	0.92	0.92	0.95	0.97	0.89	0.92
	Specific agreement on a positive rating	0.24	0.83	0.81	0.70	0.72	0.26	0.84	0.83	0.73	0.73
	Proportion staying in the cluster	0.21	0.84	0.85	0.70	0.74	0.23	0.84	0.83	0.74	0.76
**4-6 years**	Sensitivity	0.24	0.80	0.79	0.71	0.69	0.24	0.84	0.83	0.75	0.70
	Specificity	0.93	0.94	0.94	0.87	0.92	0.93	0.95	0.96	0.87	0.92
	Specific agreement on a positive rating	0.27	0.79	0.75	0.71	0.70	0.27	0.83	0.82	0.73	0.70
	Proportion staying in the cluster	0.11	0.73	0.73	0.57	0.61	0.12	0.77	0.74	0.63	0.63
**6-8 years**	Sensitivity	0.29	0.82	0.73	0.68	0.69	0.26	0.83	0.76	0.70	0.72
	Specificity	0.90	0.94	0.97	0.87	0.91	0.92	0.94	0.96	0.88	0.91
	Specific agreement on a positive rating	0.29	0.80	0.77	0.69	0.67	0.27	0.81	0.78	0.70	0.69
	Proportion staying in the cluster	0.10	0.66	0.63	0.48	0.54	0.09	0.70	0.65	0.53	0.57
	**GoDARTS**										
		RHAP-SIDD	RHAP-SIRD	RHAP-MOD	RHAP-MD	RHAP-MDH	RHAP-SIDD	RHAP-SIRD	RHAP-MOD	RHAP-MD	RHAP-MDH
**2-4 years**	Sensitivity	0.25	0.86	0.74	0.67	0.67	0.32	0.88	0.76	0.70	0.73
	Specificity	0.91	0.96	0.92	0.83	0.92	0.90	0.96	0.95	0.87	0.92
	Specific agreement on a positive rating	0.30	0.84	0.70	0.64	0.67	0.36	0.85	0.76	0.69	0.69
	Proportion staying in the cluster	0.25	0.86	0.73	0.66	0.67	0.33	0.89	0.76	0.69	0.72
**4-6 years**	Sensitivity	0.22	0.83	0.73	0.65	0.60	0.26	0.87	0.74	0.67	0.72
	Specificity	0.89	0.97	0.90	0.81	0.93	0.89	0.96	0.94	0.85	0.91
	Specific agreement on a positive rating	0.26	0.83	0.68	0.60	0.62	0.30	0.84	0.74	0.65	0.68
	Proportion staying in the cluster	0.11	0.79	0.61	0.54	0.51	0.14	0.81	0.64	0.55	0.62
**6-8 years**	Sensitivity	0.22	0.86	0.63	0.64	0.63	0.24	0.87	0.68	0.63	0.67
	Specificity	0.88	0.96	0.93	0.82	0.90	0.87	0.95	0.94	0.84	0.90
	Specific agreement on a positive rating	0.25	0.83	0.65	0.61	0.60	0.27	0.83	0.70	0.62	0.63
	Proportion staying in the cluster	0.06	0.75	0.47	0.45	0.45	0.08	0.77	0.53	0.43	0.54

DCS, Hoorn Diabetes Care System (Netherlands); GoDARTS, Genetics of Diabetes Audit and Research in Tayside Scotland (Scotland); SIDD, Severe Insulin Deficiency Diabetes; SIRD, Severe Insulin Resistance Diabetes; MOD, Mild Obesity-related Diabetes; MD, Mild Diabetes; MDH, Mild Diabetes with high HDL-cholesterol

Sensitivity indicated how many individuals belong to the particular subgroup are correctly identified as the members of that subgroup, setting the subgroup identified at diagnosis as the reference subgroup.
Specificity indicated how many individuals not belong to the particular subgroup are correctly identified as not the members of that subgroup, setting the subgroup identified at diagnosis as the reference subgroup.
Specific agreement on a positive rating indicated the answer that suppose one individual belongs to a particular subgroup at diagnosis, what is the probability that he also belongs to that subgroup in another period.
Proportion staying in the cluster indicated the proportion of individuals who never move into any other cluster since the diagnosis of diabetes.

ESM Figure 9.1 Characteristics for subgroups considering moving over time in GoDARTS

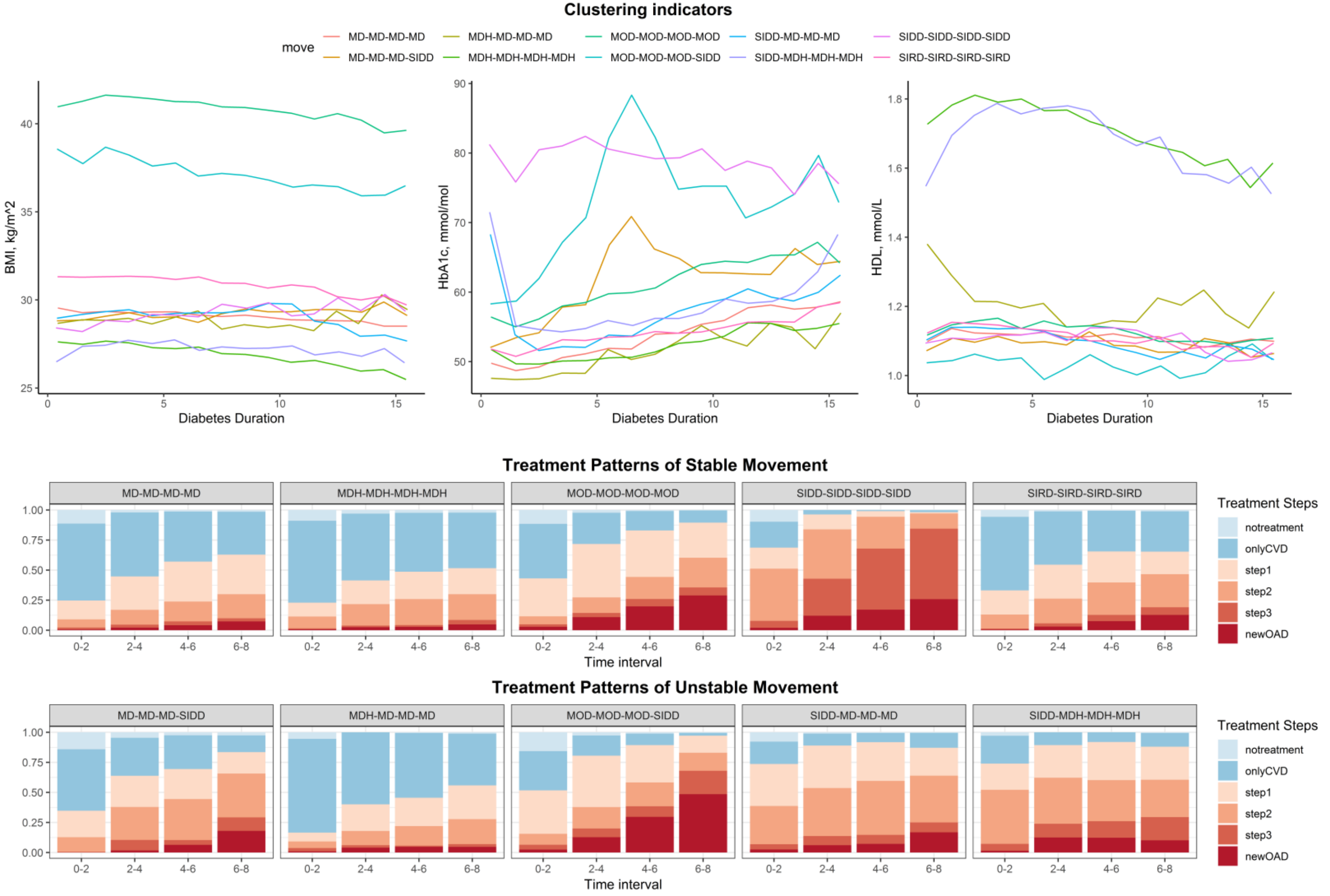

Here, SIDD, SIRD, MOD, MD and MDH refer to RHAP-SIDD, RHAP-SIRD, RHAP-MOD, RHAP-MD and RHAP-MDH, respectively.
GoDARTS, Genetics of Diabetes Audit and Research in Tayside Scotland (Scotland); SIDD, Severe Insulin Deficiency Diabetes; SIRD, Severe Insulin Resistance Diabetes; MOD, Mild Obesity-related Diabetes; MD, Mild Diabetes; MDH, Mild Diabetes with high HDL-cholesterol

ESM Figure 9.2 Subgroups' redistribution using the center-based reallocation method over time and characteristics of common trajectories in the DCS cohort (N=2,756)

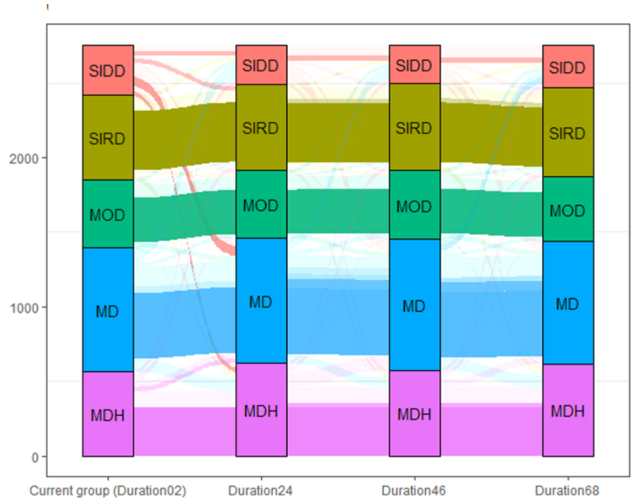

Transparency (Movement)	Proportion of baseline subgroups	Proportion of study population
SIRD-SIRD-SIRD-SIRD	70.32%	14.44%
MOD-MOD-MOD-MOD	64.85%	10.78%
MDH-MDH-MDH-MDH	56.91%	11.65%
MD-MD-MD-MD	52.89%	15.93%
SIDD-MD-MD-MD	19.82%	2.43%
SIDD-MDH-MDH-MDH	9.17%	1.12%
SIDD-SIDD-SIDD-SIDD	8.88%	1.09%
SIDD-SIRD-SIRD-SIRD	8.58%	1.05%
MDH-MD-MD-MD	5.50%	1.12%
MD-MD-MD-SIDD	5.30%	1.60%
All other movements	Each ≤ 5%	38.79% (Each ≤1.40%)

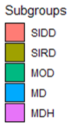

Note:
The transparency of the moving trajectories represents the frequency (indicated by proportion of baseline subgroups) of movement:
Darker trajectories indicate higher frequency, while more transparent trajectories signify lower frequency.

Here, SIDD, SIRD, MOD, MD and MDH refer to RHAP-SIDD, RHAP-SIRD, RHAP-MOD, RHAP-MD and RHAP-MDH, respectively.
DCS, Hoorn Diabetes Care System (Netherlands); SIDD, Severe Insulin Deficiency Diabetes; SIRD, Severe Insulin Resistance Diabetes; MOD, Mild Obesity-related Diabetes; MD, Mild Diabetes; MDH, Mild Diabetes with high HDL-cholesterol
The figure shows the subgroups identified based on clinical characteristics within the first 2, 2–4, 4–6 and 6–8 years of diagnosis of type 2 diabetes, represented as Duration02, Duration24, Duration46 and Duration68, respectively, along with the top ten most frequent moving trajectories using the centre-based reallocation approach. Only individuals with information available for all four periods were included in the redistribution graph.

ESM Figure 9.3 Characteristics for subgroups considering moving over time in DCS

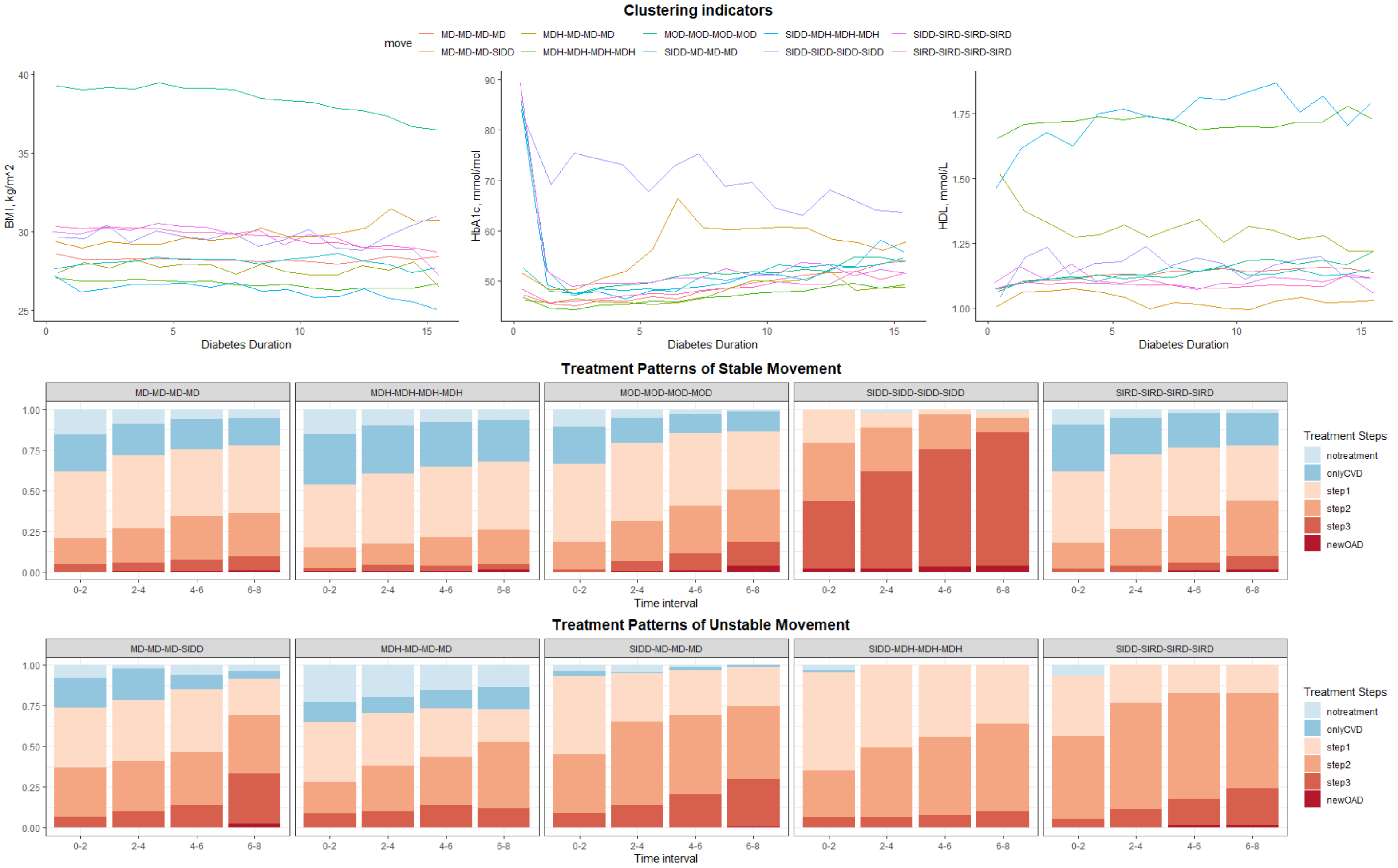

Here, SIDD, SIRD, MOD, MD and MDH refer to RHAP-SIDD, RHAP-SIRD, RHAP-MOD, RHAP-MD and RHAP-MDH, respectively.
DCS, Hoorn Diabetes Care System (Netherlands); SIDD, Severe Insulin Deficiency Diabetes; SIRD, Severe Insulin Resistance Diabetes; MOD, Mild Obesity-related Diabetes; MD, Mild Diabetes; MDH, Mild Diabetes with high HDL-cholesterol

ESM Figure 9.4 Cox regression analysis comparing event rates between mild and severe subgroups in GoDARTS

Movement	Events	HR(95% CI)	P value	Adjusted HR(95% CI)	Adjusted P Value
AMI					
Mild - Mild	601 (22.19 %)	0.62 (0.55 to 0.71)	0	0.45 (0.26 to 0.81)	0
Mild - Severe	144 (31.72 %)	0.93 (0.77 to 1.13)	0.475	1.15 (0.54 to 2.42)	0.498
Severe - Mild	141 (22.96 %)	0.62 (0.51 to 0.75)	0	0.70 (0.31 to 1.56)	0
CHF					
Mild - Mild	208 (7.68 %)	0.59 (0.48 to 0.74)	0	0.75 (0.49 to 1.16)	0.002
Mild - Severe	52 (11.45 %)	0.83 (0.60 to 1.14)	0.249	2.29 (1.35 to 3.88)	0.431
Severe - Mild	61 (9.93 %)	0.74 (0.55 to 1.00)	0.052	0.86 (0.44 to 1.69)	0.603
PVD					
Mild - Mild	25 (0.92 %)	0.56 (0.31 to 1.03)	0.064	0.63 (0.25 to 1.57)	0.176
Mild - Severe	<5	0.25 (0.06 to 1.07)	0.062	0.93 (0.24 to 3.58)	0.13
Severe - Mild	6 (0.98 %)	0.58 (0.23 to 1.46)	0.247	0.48 (0.10 to 2.32)	0.45
Stroke					
Mild - Mild	276 (10.19 %)	0.75 (0.62 to 0.92)	0.005	1.08 (0.69 to 1.68)	0.273
Mild - Severe	51 (11.23 %)	0.79 (0.57 to 1.08)	0.144	1.57 (0.82 to 3.02)	0.735
Severe - Mild	61 (9.93 %)	0.70 (0.52 to 0.95)	0.021	0.80 (0.38 to 1.69)	0.332
CKD					
Mild - Mild	1528 (56.43 %)	0.56 (0.51 to 0.61)	0	0.62 (0.54 to 0.71)	0
Mild - Severe	262 (57.71 %)	0.56 (0.48 to 0.64)	0	0.62 (0.48 to 0.81)	0.001
Severe - Mild	363 (59.12 %)	0.60 (0.53 to 0.67)	0	0.64 (0.51 to 0.80)	0
ESRD					
Mild - Mild	20 (0.74 %)	0.39 (0.21 to 0.72)	0.003	0.58 (0.16 to 2.08)	0.01
Mild - Severe	<5	0.10 (0.01 to 0.76)	0.026	0.00 (0.00 to Inf)	0.217
Severe - Mild	<5	0.16 (0.04 to 0.70)	0.015	0.58 (0.07 to 5.09)	0.116

Reference: Severe - Severe

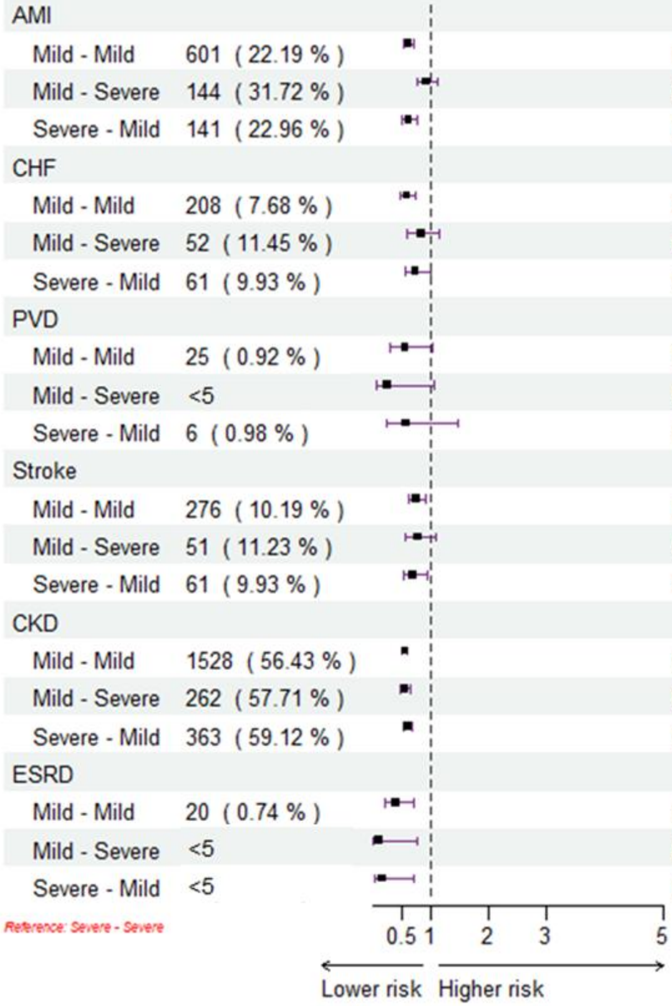

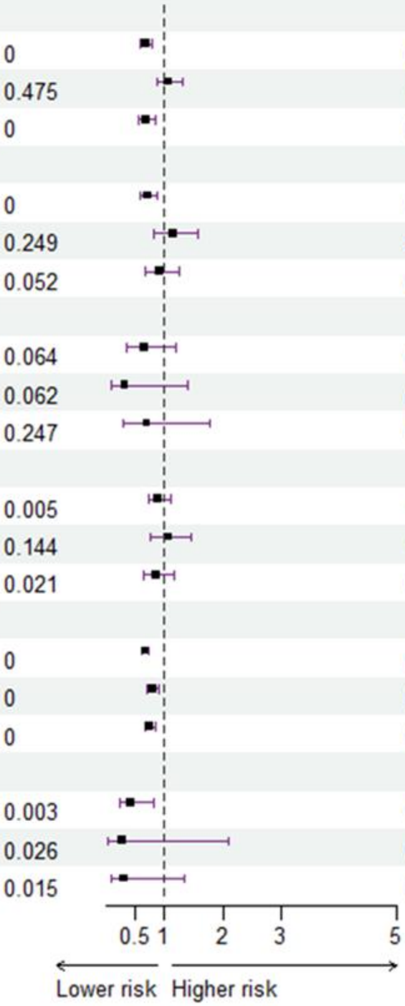

GoDARTS, Genetics of Diabetes Audit and Research in Tayside Scotland (Scotland); AMI, Acute Myocardial Infarction; CHF, Congestive Heart Failure; PVD, Peripheral Vascular Diseases; CKD, Chronic Kidney Disease; ESRD, End Stage Renal Disease
The adjusted HR and P value reflect the results after adjustment for age and sex.

ESM Figure 9.5 Cox regression analysis comparing event rates between mild and severe subgroups in DCS

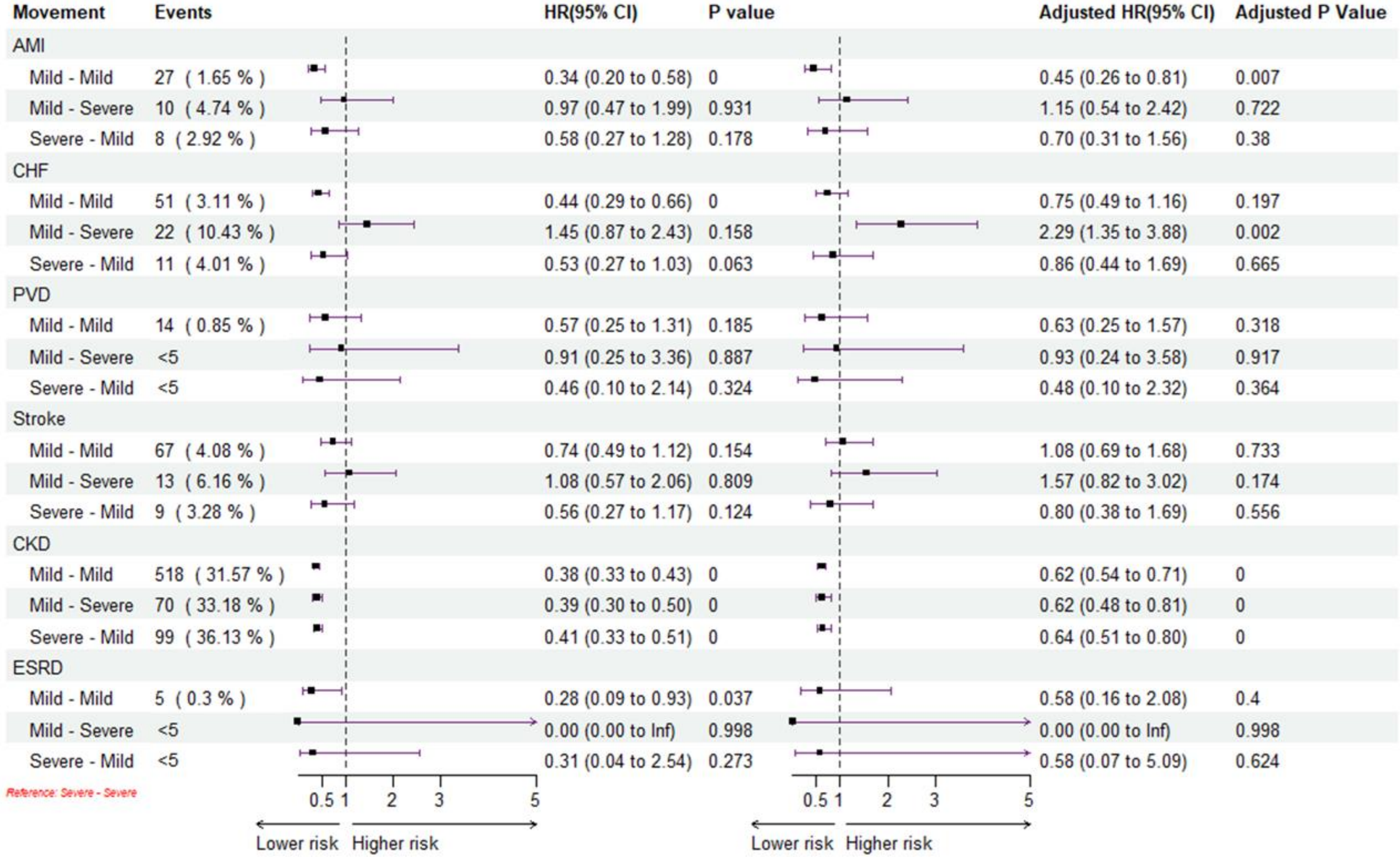

Movement	Events	HR(95% CI)	P value	Adjusted HR(95% CI)	Adjusted P Value
AMI					
Mild - Mild	27 (1.65 %)	0.34 (0.20 to 0.58)	0	0.45 (0.26 to 0.81)	0.007
Mild - Severe	10 (4.74 %)	0.97 (0.47 to 1.99)	0.931	1.15 (0.54 to 2.42)	0.722
Severe - Mild	8 (2.92 %)	0.58 (0.27 to 1.28)	0.178	0.70 (0.31 to 1.56)	0.38
CHF					
Mild - Mild	51 (3.11 %)	0.44 (0.29 to 0.66)	0	0.75 (0.49 to 1.16)	0.197
Mild - Severe	22 (10.43 %)	1.45 (0.87 to 2.43)	0.158	2.29 (1.35 to 3.88)	0.002
Severe - Mild	11 (4.01 %)	0.53 (0.27 to 1.03)	0.063	0.86 (0.44 to 1.69)	0.665
PVD					
Mild - Mild	14 (0.85 %)	0.57 (0.25 to 1.31)	0.185	0.63 (0.25 to 1.57)	0.318
Mild - Severe	<5	0.91 (0.25 to 3.36)	0.887	0.93 (0.24 to 3.58)	0.917
Severe - Mild	<5	0.46 (0.10 to 2.14)	0.324	0.48 (0.10 to 2.32)	0.364
Stroke					
Mild - Mild	67 (4.08 %)	0.74 (0.49 to 1.12)	0.154	1.08 (0.69 to 1.68)	0.733
Mild - Severe	13 (6.16 %)	1.08 (0.57 to 2.06)	0.809	1.57 (0.82 to 3.02)	0.174
Severe - Mild	9 (3.28 %)	0.56 (0.27 to 1.17)	0.124	0.80 (0.38 to 1.69)	0.556
CKD					
Mild - Mild	518 (31.57 %)	0.38 (0.33 to 0.43)	0	0.62 (0.54 to 0.71)	0
Mild - Severe	70 (33.18 %)	0.39 (0.30 to 0.50)	0	0.62 (0.48 to 0.81)	0
Severe - Mild	99 (36.13 %)	0.41 (0.33 to 0.51)	0	0.64 (0.51 to 0.80)	0
ESRD					
Mild - Mild	5 (0.3 %)	0.28 (0.09 to 0.93)	0.037	0.58 (0.16 to 2.08)	0.4
Mild - Severe	<5	0.00 (0.00 to Inf)	0.998	0.00 (0.00 to Inf)	0.998
Severe - Mild	<5	0.31 (0.04 to 2.54)	0.273	0.58 (0.07 to 5.09)	0.624

DCS, Hoorn Diabetes Care System (Netherlands); AMI, Acute Myocardial Infarction; CHF, Congestive Heart Failure; PVD, Peripheral Vascular Diseases; CKD, Chronic Kidney Disease; ESRD, End Stage Renal Disease
The adjusted HR and P value reflect the results after adjustment for age and sex.

ESM Figure 9.6 Cox regression analysis comparing event rates between mild and severe subgroups with known baseline subgroups in GoDARTS in 2 years

Movement	Events	HR(95% CI)	P value	Adjusted HR(95% CI)	Adjusted P Value
BSL:Severe Subgroups					
AMI	377 (32.98 %)	1.62 (1.34 to 1.64)	0	1.42 (0.17 to 1.73)	0.001
CHF	138 (12.07 %)	1.35 (1.00 to 1.82)	0.052	1.04 (0.77 to 1.42)	0.783
PVD	18 (1.57 %)	1.65 (0.66 to 4.16)	0.288	1.35 (0.52 to 3.47)	0.538
Stroke	148 (12.95 %)	1.41 (1.05 to 1.91)	0.023	1.13 (0.83 to 1.54)	0.424
CKD	863 (75.5 %)	1.67 (1.48 to 1.89)	0	1.33 (1.17 to 1.50)	0
ESRD	20 (1.75 %)	6.1 (1.43 to 26.12)	0.015	3.04 (0.70 to 13.18)	0.136
BSL:Mild Subgroups					
AMI	144 (31.72 %)	1.50 (1.25 to 1.80)	0	1.56 (1.30 to 1.87)	0
CHF	52 (11.45 %)	1.38 (1.02 to 1.88)	0.036	1.55 (1.14 to 2.11)	0.005
PVD	<5	0.43 (0.10 to 1.81)	0.249	0.48 (0.11 to 2.02)	0.314
Stroke	51 (11.23 %)	1.06 (0.78 to 1.42)	0.721	1.18 (0.88 to 1.60)	0.271
CKD	262 (57.71 %)	1.00 (0.87 to 1.14)	0.968	1.18 (1.03 to 1.35)	0.015
ESRD	<5	0.27 (0.04 to 2.00)	0.199	0.67 (0.08 to 5.26)	0.701

Reference: Move towards/stay in Mild subgroups in 2 years

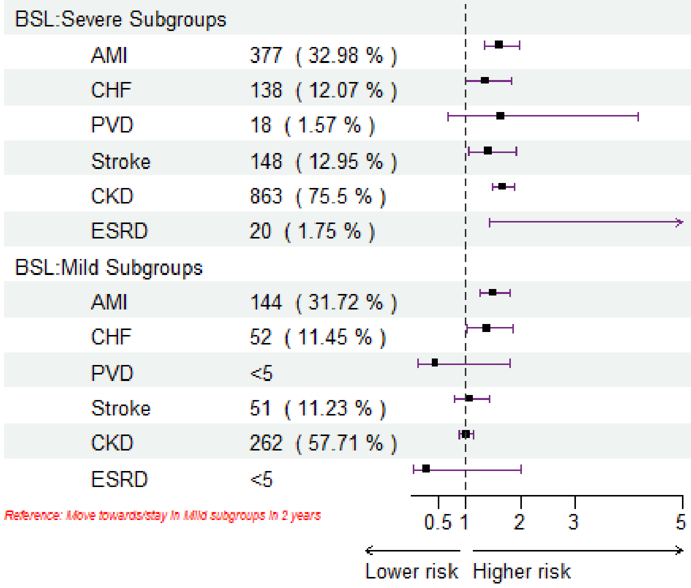

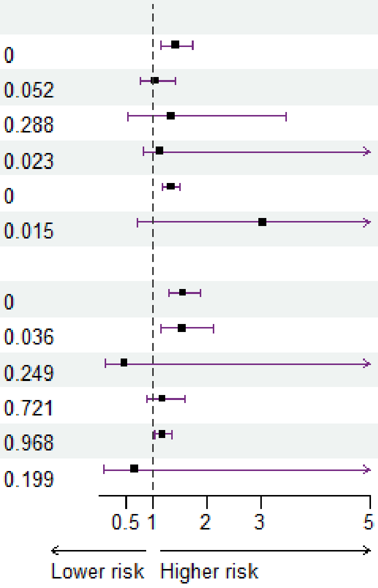

GoDARTS, Genetics of Diabetes Audit and Research in Tayside Scotland (Scotland); BSL, Baseline; AMI, Acute Myocardial Infarction; CHF, Congestive Heart Failure; PVD, Peripheral Vascular Diseases; CKD, Chronic Kidney Disease; ESRD, End Stage Renal Disease
The adjusted HR and P value reflect the results after adjustment for age and sex.

ESM Figure 9.7 Cox regression analysis comparing event rates between mild and severe subgroups with known baseline subgroups in DCS in 2 years

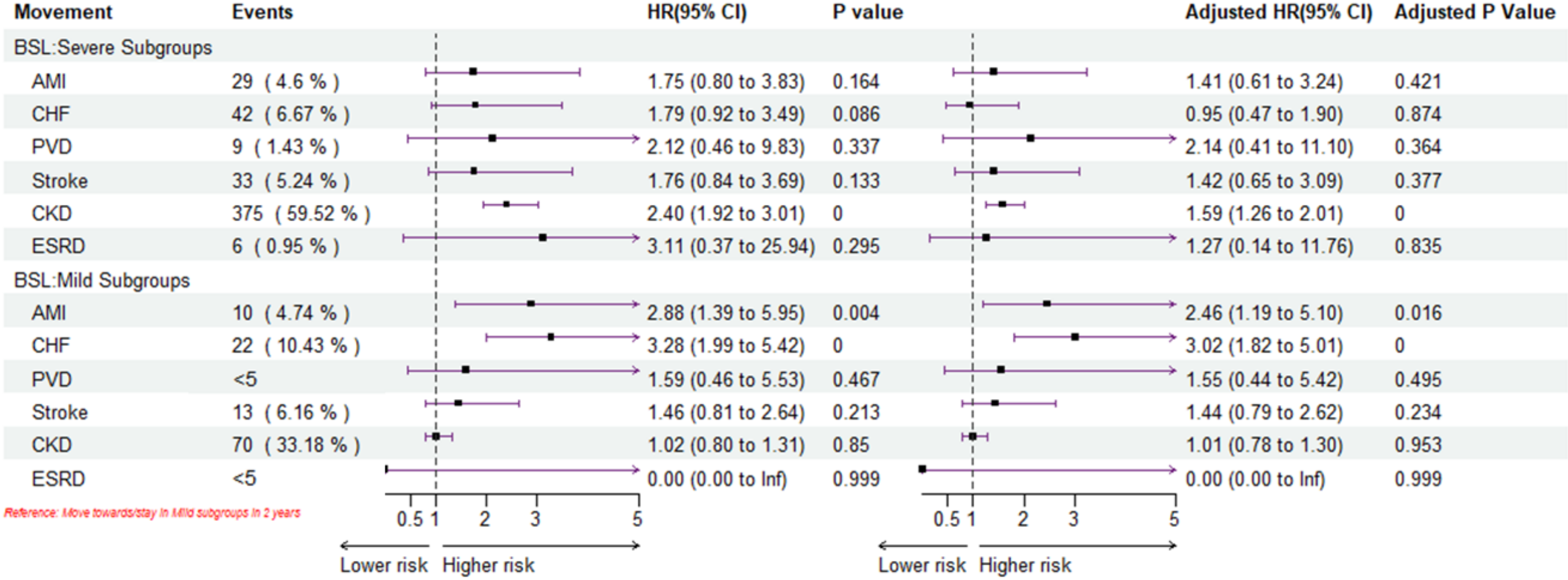

Movement	Events	HR(95% CI)	P value	Adjusted HR(95% CI)	Adjusted P Value
BSL:Severe Subgroups					
AMI	29 (4.6 %)	1.75 (0.80 to 3.83)	0.164	1.41 (0.61 to 3.24)	0.421
CHF	42 (6.67 %)	1.79 (0.92 to 3.49)	0.086	0.95 (0.47 to 1.90)	0.874
PVD	9 (1.43 %)	2.12 (0.46 to 9.83)	0.337	2.14 (0.41 to 11.10)	0.364
Stroke	33 (5.24 %)	1.76 (0.84 to 3.69)	0.133	1.42 (0.65 to 3.09)	0.377
CKD	375 (59.52 %)	2.40 (1.92 to 3.01)	0	1.59 (1.26 to 2.01)	0
ESRD	6 (0.95 %)	3.11 (0.37 to 25.94)	0.295	1.27 (0.14 to 11.76)	0.835
BSL:Mild Subgroups					
AMI	10 (4.74 %)	2.88 (1.39 to 5.95)	0.004	2.46 (1.19 to 5.10)	0.016
CHF	22 (10.43 %)	3.28 (1.99 to 5.42)	0	3.02 (1.82 to 5.01)	0
PVD	<5	1.59 (0.46 to 5.53)	0.467	1.55 (0.44 to 5.42)	0.495
Stroke	13 (6.16 %)	1.46 (0.81 to 2.64)	0.213	1.44 (0.79 to 2.62)	0.234
CKD	70 (33.18 %)	1.02 (0.80 to 1.31)	0.85	1.01 (0.78 to 1.30)	0.953
ESRD	<5	0.00 (0.00 to Inf)	0.999	0.00 (0.00 to Inf)	0.999

DCS, Hoorn Diabetes Care System (Netherlands); BSL, Baseline; AMI, Acute Myocardial Infarction; CHF, Congestive Heart Failure; PVD, Peripheral Vascular Diseases; CKD, Chronic Kidney Disease; ESRD, End Stage Renal Disease

The adjusted HR and P value reflect the results after adjustment for age and sex.

ESM Appendix 10 The number of observed individuals over time

ESM Figure 10.1 The number of observed individuals over time in DCS

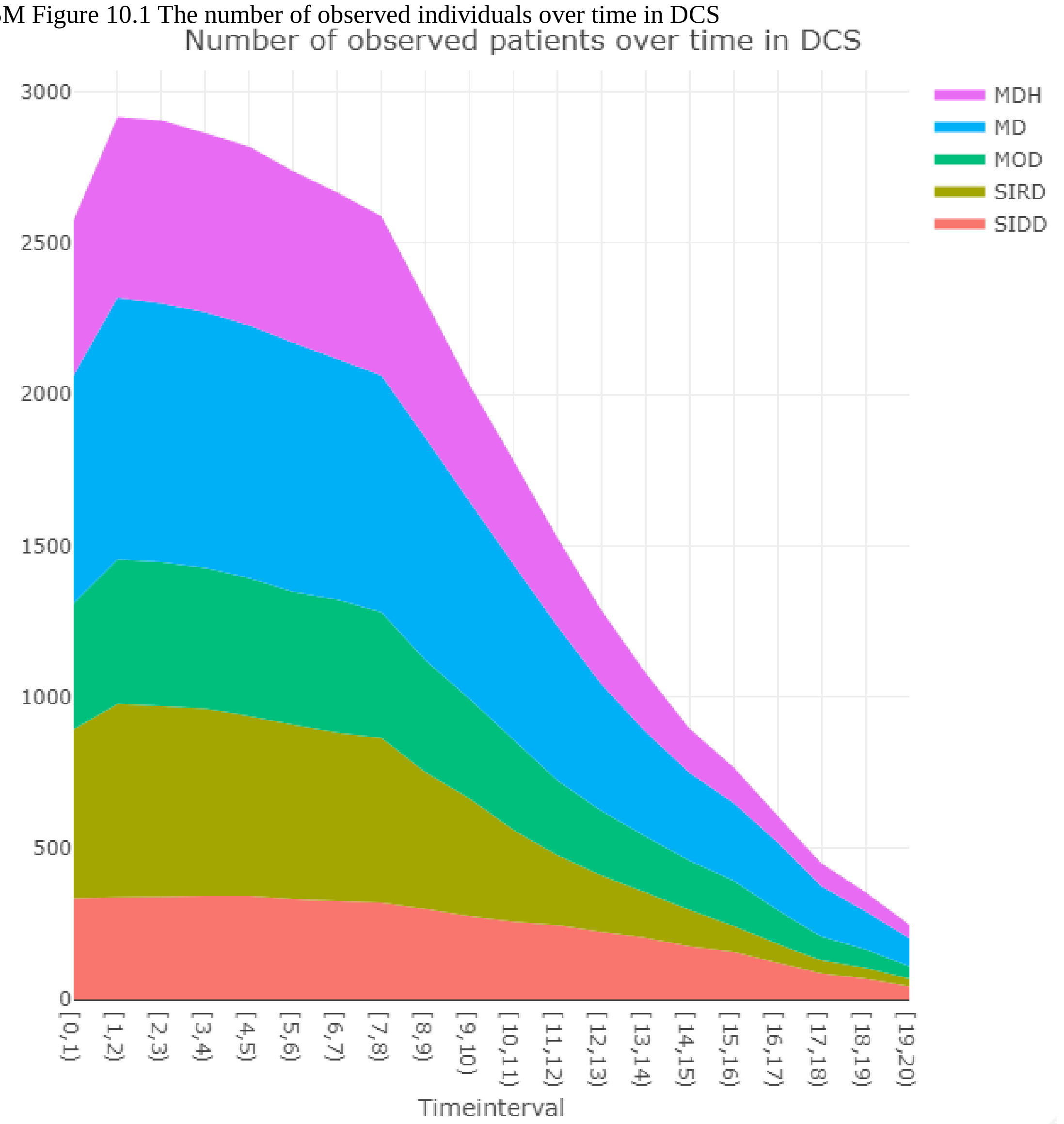

Here, SIDD, SIRD, MOD, MD and MDH refer to RHAP-SIDD, RHAP-SIRD, RHAP-MOD, RHAP-MD and RHAP-MDH, respectively.

DCS, Hoorn Diabetes Care System (Netherlands); SIDD, Severe Insulin Deficiency Diabetes; SIRD, Severe Insulin Resistance Diabetes; MOD, Mild Obesity-related Diabetes; MD, Mild Diabetes; MDH, Mild Diabetes with high HDL-cholesterol

ESM Figure 10.2 The number of observed individuals over time in GoDARTS

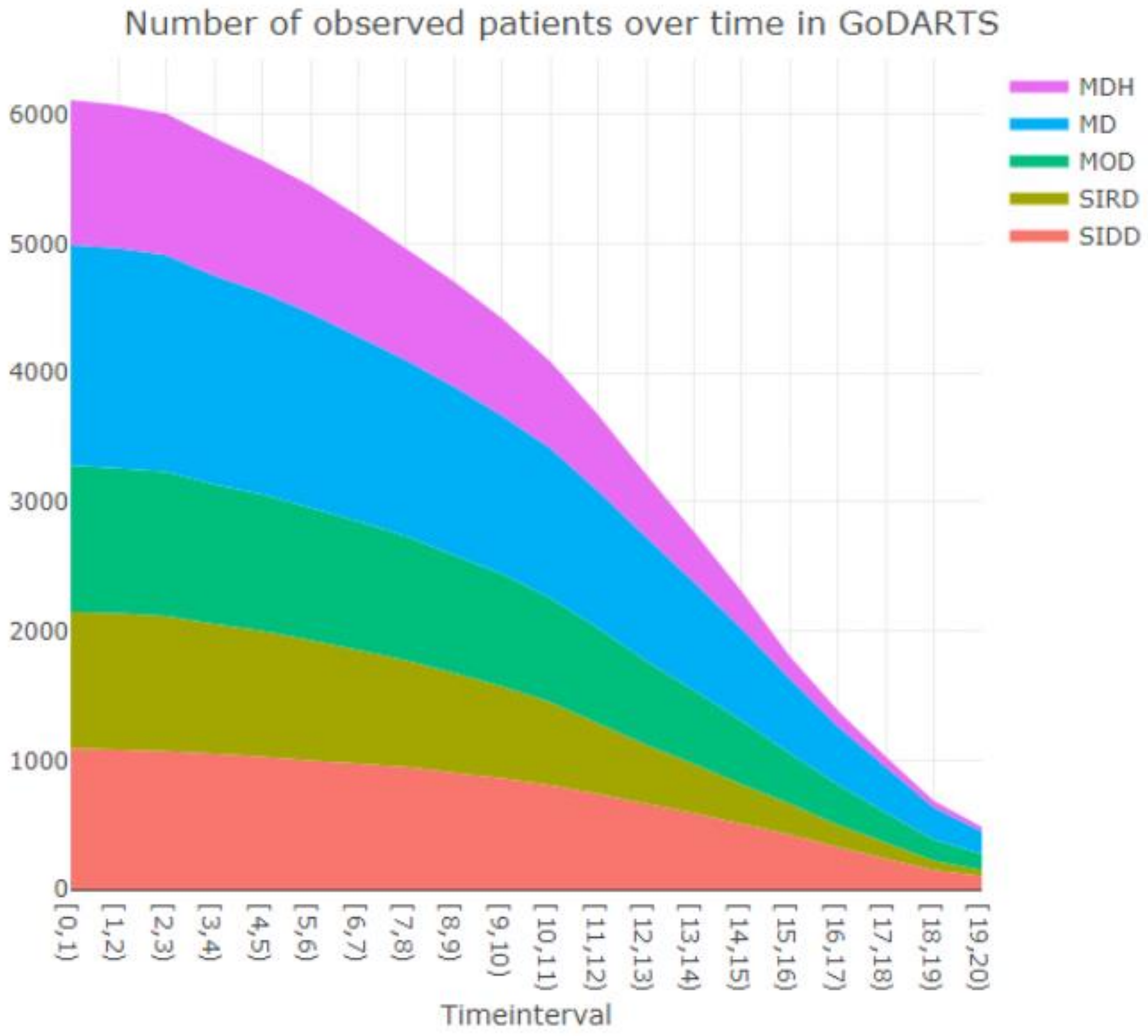

Here, SIDD, SIRD, MOD, MD and MDH refer to RHAP-SIDD, RHAP-SIRD, RHAP-MOD, RHAP-MD and RHAP-MDH, respectively.

GoDARTS, Genetics of Diabetes Audit and Research in Tayside Scotland (Scotland); SIDD, Severe Insulin Deficiency Diabetes; SIRD, Severe Insulin Resistance Diabetes; MOD, Mild Obesity-related Diabetes; MD, Mild Diabetes; MDH, Mild Diabetes with high HDL-cholesterol

ESM Appendix 11 Consistency of subgroups classification compared to Ahlqvist et al.'s subgroups [3]

ESM Figure 11.1 Subgroups' consistency compared to Ahlqvist et al.'s subgroups [3] in DCS

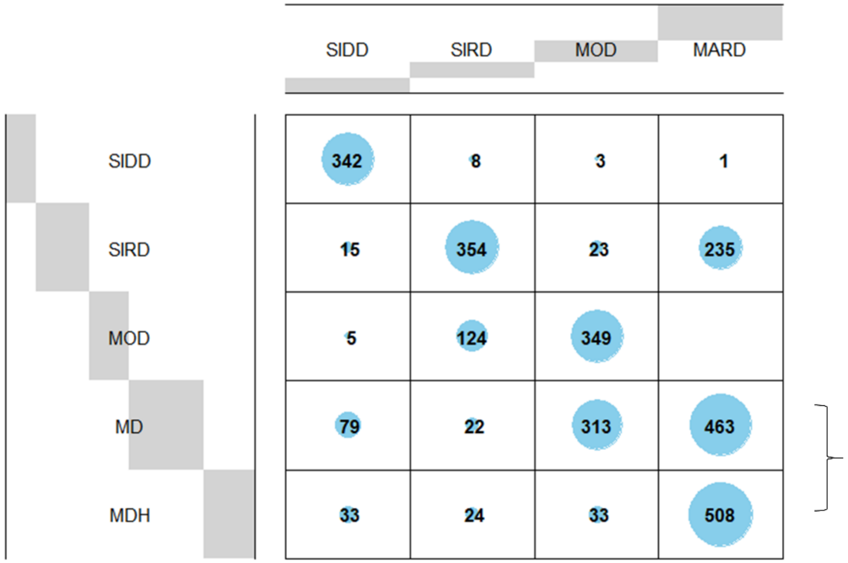

Sensitivity	Specificity
0.72 (0.68,0.76)	1 (0.99,1.00)
0.67 (0.62,0.71)	0.89 (0.87,0.90)
0.48 (0.45,0.52)	0.94 (0.93,0.95)
0.8 (0.78,0.83)	0.71 (0.69,0.73)

Here, in the vertical line, SIDD, SIRD, MOD, MD and MDH refer to RHAP-SIDD, RHAP-SIRD, RHAP-MOD, RHAP-MD and RHAP-MDH, respectively.
DCS, Hoorn Diabetes Care System (Netherlands); SIDD, Severe Insulin Deficiency Diabetes; SIRD, Severe Insulin Resistance Diabetes; MOD, Mild Obesity-related Diabetes; MD, Mild Diabetes; MDH, Mild Diabetes with high HDL-cholesterol; MARD, Mild Age-Related Diabetes
To analyze the difference caused by different data-driven clustering approaches, individuals in DCS were also assigned to one of four subgroups following Ahlqvist et al. [3], including SIDD, SIRD, MOD, and MARD based on sex-specific k-means clustering by five scaled clustering indicators including age at diagnosis, BMI, HbA_{1c}, and HOMA estimates [18] of β-cell function and insulin resistance by C-peptide and fasting glucose. Agreement between clusters was assessed based on the specificity and sensitivity.

References

[1] Slieker RC, Donnelly LA, Fitipaldi H, et al. (2021) Replication and cross-validation of type 2 diabetes subtypes based on clinical variables: an IMI-RHAPSODY study. Diabetologia 64(9): 1982-1989. 10.1007/s00125-021-05490-8

[2] Jones AG, Lonergan M, Rodgers LR, et al. (2015) Studies of diabetes treatment stratification should correct for baseline HbA1c: a MASTERMIND study. Diabetic Medicine 32: 94-94

[3] Ahlqvist E, Storm P, Karajamaki A, et al. (2018) Novel subgroups of adult-onset diabetes and their association with outcomes: a data-driven cluster analysis of six variables. Lancet Diabetes & Endocrinology 6(5): 361-369. 10.1016/S2213-8587(18)30051-2

[4] van der Heijden AA, Rauh SP, Dekker JM, et al. (2017) The Hoorn Diabetes Care System (DCS) cohort. A prospective cohort of persons with type 2 diabetes treated in primary care in the Netherlands. BMJ Open 7(5): e015599. 10.1136/bmjopen-2016-015599

[5] Hebert HL, Shepherd B, Milburn K, et al. (2018) Cohort Profile: Genetics of Diabetes Audit and Research in Tayside Scotland (GoDARTS). Int J Epidemiol 47(2): 380-+. 10.1093/ije/dyx140

[6] Rodriguez RM, Luis-Lima S, Fernandez JM, et al. (2022) Estimated GFR in autosomal dominant polycystic kidney disease: errors of an unpredictable method. J Nephrol 35(8): 2109-2118. 10.1007/s40620-022-01286-0

[7] Levey AS, Stevens LA, Schmid CH, et al. (2009) A New Equation to Estimate Glomerular Filtration Rate. Annals of Internal Medicine 150(9): 604-612. 10.7326/0003-4819-150-9-200905050-00006

[8] Sievert. C (2020) Interactive Web-Based Data Visualization with R, plotly, and shiny.

[9] Wickham. H (2016) ggplot2: Elegant Graphics for Data Analysis.

[10] Patil I (2021) Visualizations with statistical details: The 'ggstatsplot' approach. Journal of Open Source Software. 10.21105/joss.03167

[11] Team RC (2021) R: A language and environment for statistical computing.

[12] Kuhn M (2021) caret: Classification and Regression Training.

[13] Douglas Bates MM, Ben Bolker, Steve Walker (2015) Fitting Linear Mixed-Effects Models Using lme4. J Stat Softw 67(1). 10.18637/jss.v067.i01.

[14] Alboukadel Kassambara MKaPB (2021) survminer: Drawing Survival Curves using 'ggplot2'.

[15] T T (2021) A Package for Survival Analysis in R.

[16] Yee TW (2015) Vector Generalized Linear and Additive Models: With an Implementation in R.

[17] Dennis JM, Shields BM, Henley WE, Jones AG, Hattersley AT (2019) Disease progression and treatment response in data-driven subgroups of type 2 diabetes compared with models based on simple clinical features: an analysis using clinical trial data. Lancet Diabetes & Endocrinology 7(6): 442-451. 10.1016/S2213-8587(19)30087-7

[18] Holman R, Hines G, Kennedy I, Stevens R, Matthews D, Levy J (2004) A calculator for HOMA. Diabetologia 47: A222-A222
